# Real-time location of acupuncture points based on anatomical landmarks and pose estimation models

**DOI:** 10.3389/fnbot.2024.1484038

**Published:** 2024-11-08

**Authors:** Hadi Sedigh Malekroodi, Seon-Deok Seo, Jinseong Choi, Chang-Soo Na, Byeong-il Lee, Myunggi Yi

**Affiliations:** ^1^Industry 4.0 Convergence Bionics Engineering, Pukyong National University, Busan, Republic of Korea; ^2^Major of Biomedical Engineering, Division of Smart Healthcare, Pukyong National University, Busan, Republic of Korea; ^3^College of Korean Medicine, Dongshin University, Naju, Republic of Korea; ^4^Major of Human Bioconvergence, Division of Smart Healthcare, Pukyong National University, Busan, Republic of Korea; ^5^Digital of Healthcare Research Center, Institute of Information Technology and Convergence, Pukyong National University, Busan, Republic of Korea

**Keywords:** deep learning, acupuncture, traditional medicine, computer vision, pose estimation

## Abstract

**Introduction:**

Precise identification of acupuncture points (acupoints) is essential for effective treatment, but manual location by untrained individuals can often lack accuracy and consistency. This study proposes two approaches that use artificial intelligence (AI) specifically computer vision to automatically and accurately identify acupoints on the face and hand in real-time, enhancing both precision and accessibility in acupuncture practices.

**Methods:**

The first approach applies a real-time landmark detection system to locate 38 specific acupoints on the face and hand by translating anatomical landmarks from image data into acupoint coordinates. The second approach uses a convolutional neural network (CNN) specifically optimized for pose estimation to detect five key acupoints on the arm and hand (LI11, LI10, TE5, TE3, LI4), drawing on constrained medical imaging data for training. To validate these methods, we compared the predicted acupoint locations with those annotated by experts.

**Results:**

Both approaches demonstrated high accuracy, with mean localization errors of less than 5 mm when compared to expert annotations. The landmark detection system successfully mapped multiple acupoints across the face and hand even in complex imaging scenarios. The data-driven approach accurately detected five arm and hand acupoints with a mean Average Precision (mAP) of 0.99 at OKS 50%.

**Discussion:**

These AI-driven methods establish a solid foundation for the automated localization of acupoints, enhancing both self-guided and professional acupuncture practices. By enabling precise, real-time localization of acupoints, these technologies could improve the accuracy of treatments, facilitate self-training, and increase the accessibility of acupuncture. Future developments could expand these models to include additional acupoints and incorporate them into intuitive applications for broader use.

## 1 Introduction

Acupuncture is an ancient medical technique is a practice with roots extending back thousands of years. It involves the precise insertion of thin, sterile needles into specific points on the body known as acupoints (Formenti et al., 2023; Hou et al., 2015). These acupoints lie along meridians, or pathways, that are believed to facilitate the flow of vital energy, known as qi or chi, throughout the body. By stimulating these acupoints, acupuncture aims to balance qi flow and promote healing. These points are not visible to the naked eye but are identified based on anatomical landmarks, palpation (feeling for subtle depressions or sensitivities), and traditional knowledge passed down through generations of practitioners (Tegiacchi, 2021).

In traditional medicine, acupuncture is used to treat various conditions including chronic pain, nausea, allergies, anxiety, depression, infertility, and more (Formenti et al., 2023; Yang et al., 2011). It is thought to work by releasing natural painkillers called endorphins, regulating blood flow, stimulating nerves and connective tissue, altering brain chemistry, and affecting hormone release (Wang et al., 2022; Vanderploeg and Yi, 2009). There are hundreds of acupoints located throughout the body, each associated with specific meridians and therapeutic effects (Zhang B. et al., 2022). For example, acupoint LI4 (Hegu), located between the thumb and index finger, is commonly used to relieve headaches and toothaches (Lu and Lu, 2008). Some practitioners even suggest its potential benefits for managing symptoms associated with Parkinson’s disease (Park et al., 2023). Once dismissed by Western medicine, acupuncture has gained more mainstream acceptance in recent decades. In 1997, the National Institutes of Health found acupuncture to be effective for nausea and other conditions. Since then, clinical trials have demonstrated its efficacy for various health issues (Mayer, 2000; Mao and Khanna, 2012). Today, acupuncture is practiced worldwide including in Western countries. It is one of the most widely used forms of alternative and complementary medicine (Yang et al., 2011; Wang et al., 2022).

Traditionally, acupuncturists locate acupuncture points by feeling for specific landmarks on the body, such as bony protrusions or muscle lines. However, manual identification depends heavily on the experience of the acupuncturist and can suffer from inaccuracy, and can be time-consuming. Technology may be able to improve acupoint localization. Artificial intelligence (AI) can be used to revolutionize the practice of acupuncture. One of the most promising applications of AI in acupuncture is the use of computer vision to locate acupuncture points (Wang et al., 2022; Sun et al., 2020; Zhang M. et al., 2022). Computer vision techniques like pose estimation provide an attractive solution by automating acupoint localization in a standardized way. Pose estimation is an important computer vision task that involves detecting key points on the human body and understanding their positions and orientations. It has a wide range of applications such as human-computer interaction, augmented reality, action recognition, and motion capture (Sulong and Randles, 2023).

Recent studies have increasingly focused on leveraging computer vision techniques to automate the identification and localization of acupoints, recognizing the complexity of acupoint anatomy and the subtlety of acupoint landmarks. Deep learning approaches, particularly convolutional neural networks (CNNs), have emerged as promising tools for acupoint recognition due to their powerful feature extraction capabilities. Researchers have explored various architectures, including U-Net, cascaded networks, and improved high-resolution networks (HRNet), to enhance detection accuracy (Sun et al., 2020; Sun et al., 2022; Chan et al., 2021; Li et al., 2024; Yuan et al., 2024). In a recent study, Liu et al. (2023) introduced an improved Keypoint RCNN network was designed for back acupoint localization. By incorporating a posterior median line positioning method, the accuracy improved to 90.12%. Another significant development is the integration of anatomical measurements and proportional bone measurement methods with deep learning models to improve acupoint localization (Zhang M. et al., 2022; Chan et al., 2021). This approach combines traditional acupuncture knowledge with modern computer vision techniques.

Researchers have also explored the application of augmented reality (AR) and mixed reality (MR) technologies to visualize and localize acupoints in real-time, with systems like FaceAtlasAR and HoloLens 2-based applications showing promise. These technologies offer real-time tracking and visualization capabilities, potentially improving the practical application of automated acupoint detection systems in clinical settings (Zhang M. et al., 2022; Chen et al., 2021; Chen et al., 2017). For instance, Yang et al. (2021) developed tools like the SMART Table, which integrates 3D and AR technologies to improve acupoint education, training, and evaluation. This interactive system is designed to support both educational purposes and clinical competency assessments, showing promise in enhancing skills related to acupuncture and musculoskeletal treatments. Despite the limited number of studies in this area, several limitations persist in the current research despite recent advancements. These issues include limited datasets and accuracy problems in certain body areas. Many studies focus on a small number of acupoints or specific body regions (Sun et al., 2020; Chan et al., 2021), which restricts the applicability of their methods to comprehensive acupoint recognition.

The primary objective of this study is to develop a real-time acupuncture point detection system using state-of-the-art pose estimation models. While previous works like Sun et al. (2022) have shown promising results, our approach offers several key innovations. We explore and compare two distinct computer vision techniques: utilizing a real-time landmark detection framework to map acupoint locations based on classical proportional measurement methods, and fine-tuning a state-of-the-art pose estimation model on a custom dataset to directly predict acupoint coordinates. Our system is designed to detect a comprehensive set of acupoints, not limited small number as in previous studies. Furthermore, we develop an integrated application that enables real-time visualization of predicted acupuncture points on a webcam feed, showcasing their potential for assistive technologies in acupuncture treatment. Through this research, we aim to address several key questions: How does the accuracy of acupoint localization using a landmark-based approach compare to that of a deep learning-based pose estimation model? To what extent can these computer vision techniques be applied in real-time for practical acupuncture assistance? What are the limitations and potential improvements for each approach in the context of acupoint localization? By addressing these questions, our work aims to bridge the gap between traditional manual methods and automated computer-assisted approaches, providing acupuncturists with efficient tool to enhance their practice. This research has the potential to significantly impact acupuncture practice by improving accuracy and consistency in acupoint localization, providing a more comprehensive detection system, and offering real-time assistance to practitioners.

## 2 Materials and methods

### 2.1 Landmark detection and proportional mapping approach

**Figure 1:**
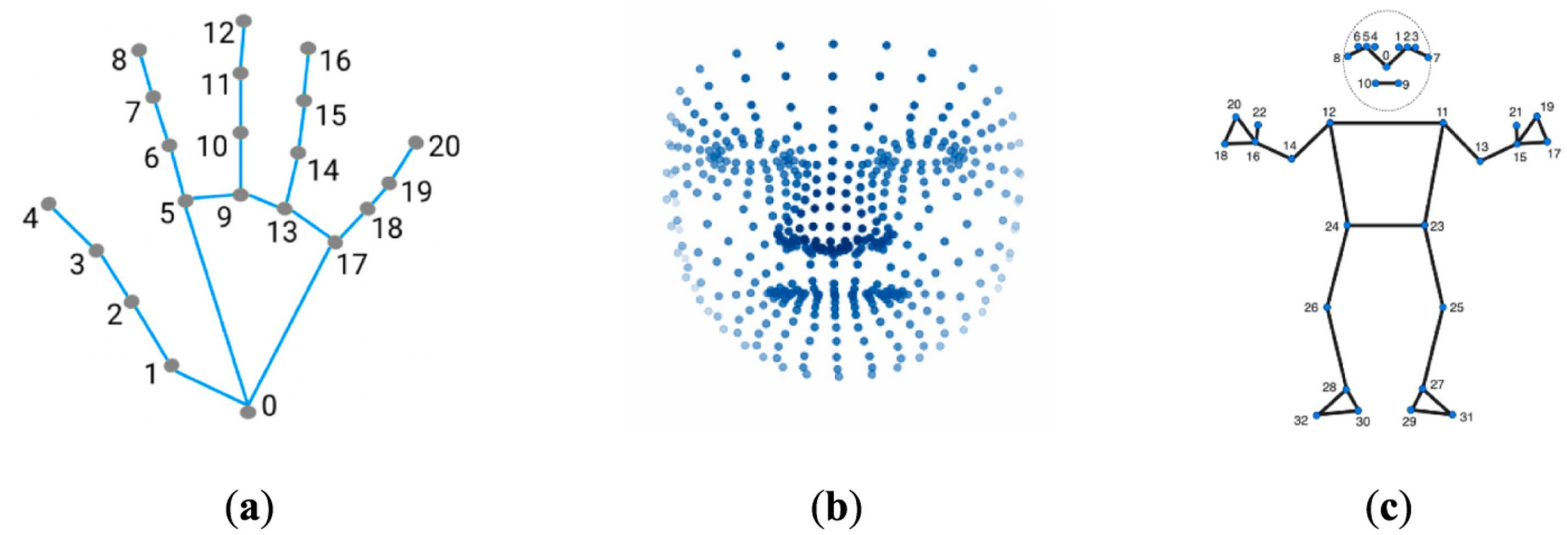
Keypoint localization examples using MediaPipe framework. **(a)** The MediaPipe Hand solution localizing 21 hand-knuckle coordinates within detected hand regions. **(b)** The MediaPipe Facemesh solution localizing 468 facial landmarks. **(c)** The MediaPipe Pose solution localizing 33 body landmarks. The figure demonstrates the capabilities of MediaPipe for anatomical keypoint localization across hands, faces, and bodies through the use of machine learning models tailored to each area.

The MediaPipe framework (Lugaresi et al., 2019), developed by Google, has garnered considerable attention in the computer vision community due to its versatility and efficiency in building real-time applications. Initially designed for hand and face tracking, MediaPipe has expanded its capabilities to cater to a wide range of pose estimation and human body tracking tasks (Figure 1). The framework’s ability to leverage deep learning models, coupled with its lightweight design, makes it an attractive choice for developing applications that require real-time performance on resource-constrained devices (Lugaresi et al., 2019). This attribute was the primary motivation for employing this framework in the present study. However, an important limitation is that MediaPipe does not provide access to the model architectures and parameters. So users cannot train the models from scratch on their own datasets. In the context of acupuncture point detection, by harnessing the framework’s capabilities, it becomes possible to develop a real-time system that can efficiently identify acupuncture points on the human body, thereby enhancing the precision and effectiveness of acupuncture treatments. In this approach we landmark coordinate data generated by the MediaPipe framework to calculate proportional acupoint locations based on formulas guided by traditional acupuncture literature (World Health Organization, 2008; Focks, 2008; National University of Korean Medicine, Graduate School of Korean Medicine, Meridian and Acupoint Studies Textbook Compilation Committee, 2020).

#### 2.1.1 Acupoint selection

A total of 38 acupoints were selected for localization including 18 acupoints on the hands and 20 acupoints on the face (Table 1). These acupoints were selected based on their common usage in clinical practice for a variety of conditions. The Supplementary Table S1, provides a summary of these acupoints included in the study, along with anatomical locations and key clinical usages.

**Table 1:** Acupuncture points selected for detection utilizing landmark detection framework.

Acupoint	Full name	Meridian	Acupoint	Full name	Meridian
Face^a^			Hand^a^		
CV-24	Chengjiang	Conception vessel	HT-7	Shenmen	Heart
BL-1	Jingming	Bladder	HT-8	Shaofu	Heart
BL-2	Cuanzhu	Bladder	HT-9	Shaochong	Heart
GB-1	Tongziliao	Gallbladder	LI-1	Shangyang	Large intestine
GB-2	Tinghui	Gallbladder	LI-2	Erjian	Large intestine
GB-14	Yangbai	Gallbladder	LI-3	Sanjian	Large intestine
GV-25	Suliao	Governing vessel	LI-4	Hegu	Large intestine
GV-26	Shuigou	Governing vessel	LU-11	Shaoshang	Lung
GV-27	Duiduan	Governing vessel	LU-9	Taiyuan	Lung
LI-19	Kouheliao	Large intestine	PC-9	Zhongchong	Pericardium
LI-20	Yingxiang	Large intestine	SI-1	Shaoze	Small intestine
SI-18	Quanliao	Small intestine	SI-2	Qiangu	Small intestine
ST-1	Chengqi	Stomach	SI-3	Houxi	Small intestine
ST-2	Sibai	Stomach	SI-4	Wangu	Small intestine
ST-3	Juliao	Stomach	TE-1	Guanchong	Triple energizer
ST-4	Dicang	Stomach	TE-2	Yemen	Triple energizer
ST-5	Daying	Stomach	TE-3	Zhongzhu	Triple energizer
ST-6	Jiache	Stomach	TE-4	Yangchi	Triple energizer
ST-7	Xiaguan	Stomach			
TE-23	Sizhukong	Triple energizer			

Provides the standard name and abbreviation for each acupuncture point.

a More detail provided in the Supplementary Table S1.

#### 2.1.2 Method

In order to identify the locations of over 38 acupoints, we utilized a combination of published literature regarding acupoint locations (World Health Organization, 2008; Focks, 2008), principles of oriental medicine, and the MediaPipe framework (v0.10.1).

**Figure 2:**
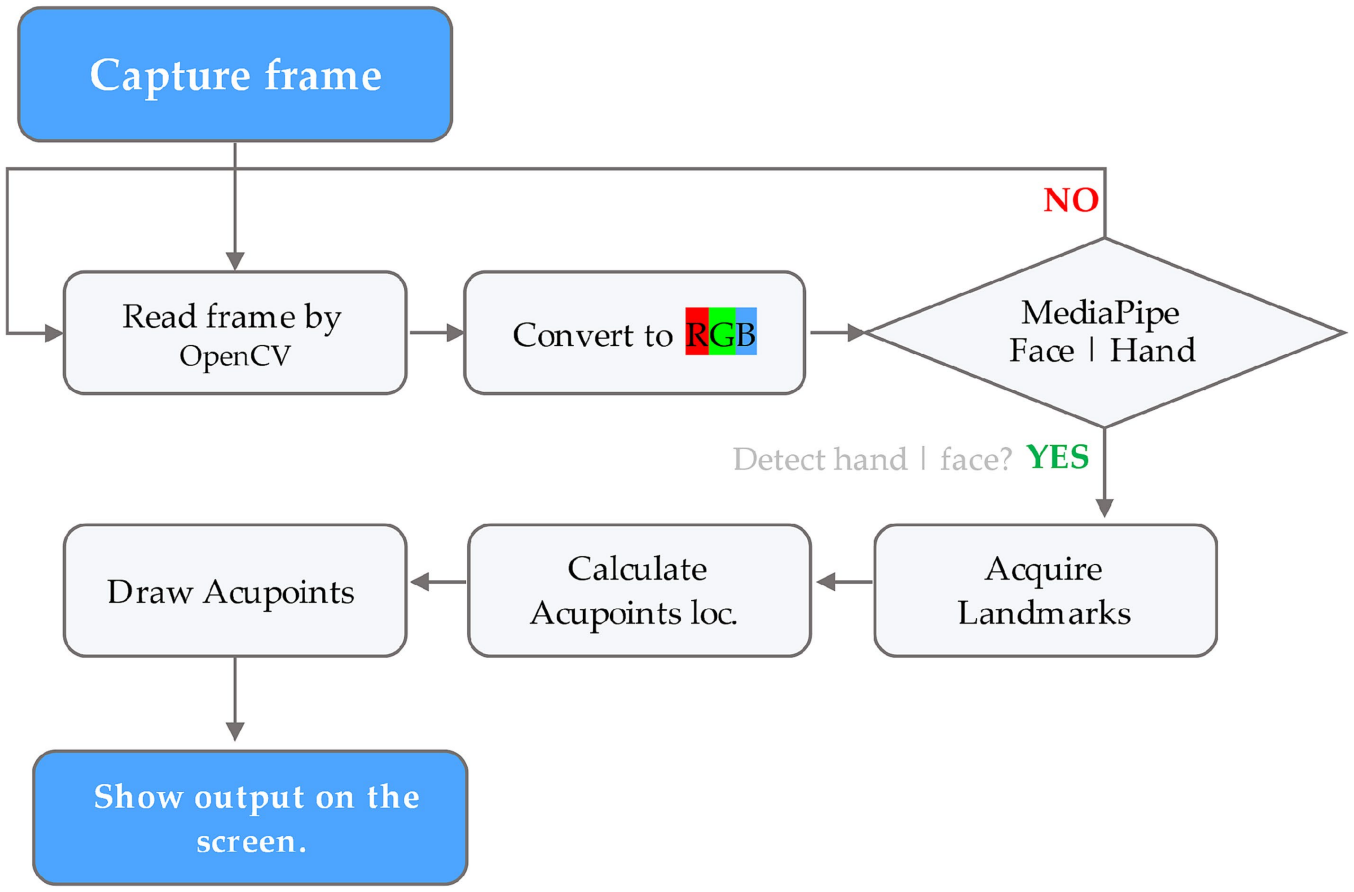
Flowchart illustrating the acupoint detection pipeline using the MediaPipe framework.

The process involved first compiling a list of acupoint locations on the hands and face by referencing established acupuncture literature and standards (Supplementary Table S1). Then, each frame of the input video was captured through OpenCV computer vision library (v4.7.0.72) and converted to RGB format. The RGB frames were input into MediaPipe Face Mesh and Hand pipelines to acquire facial and hand landmark coordinates. These 468 facial and 21 hand landmarks per hand were used to mathematically estimate locations of key acupoints based on anatomical proportionality. Small dots were drawn on the original frames at the calculated acupoint locations using OpenCV drawing functions, representing acupoints. Finally, the output frame with overlayed acupoint dots was displayed to the user in real-time via OpenCV, allowing viewing of the acupoint tracking in the live video stream (Figure 2).

**Figure 3:**
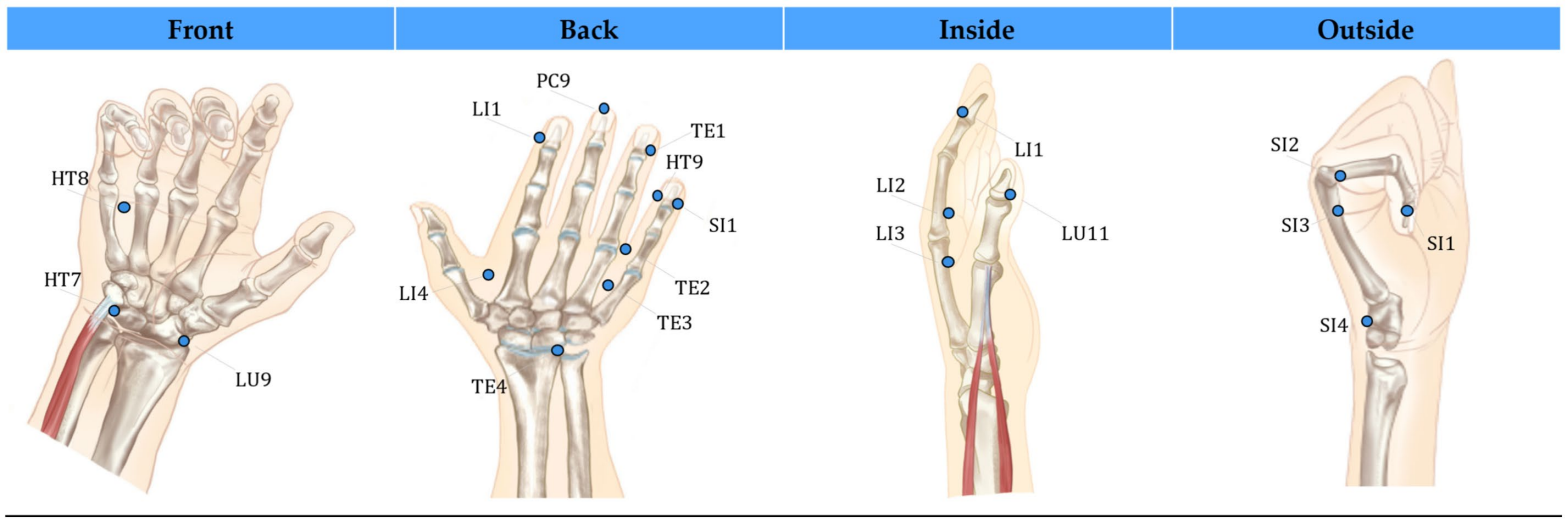
Visualization of hand acupuncture points organized by four postures. Segmenting points by posture enables clear visualization and access across hand surfaces (Images of hand from the source National University of Korean Medicine, Graduate School of Korean Medicine, Meridian and Acupoint Studies Textbook Compilation Committee, 2020).

**Figure 4:**
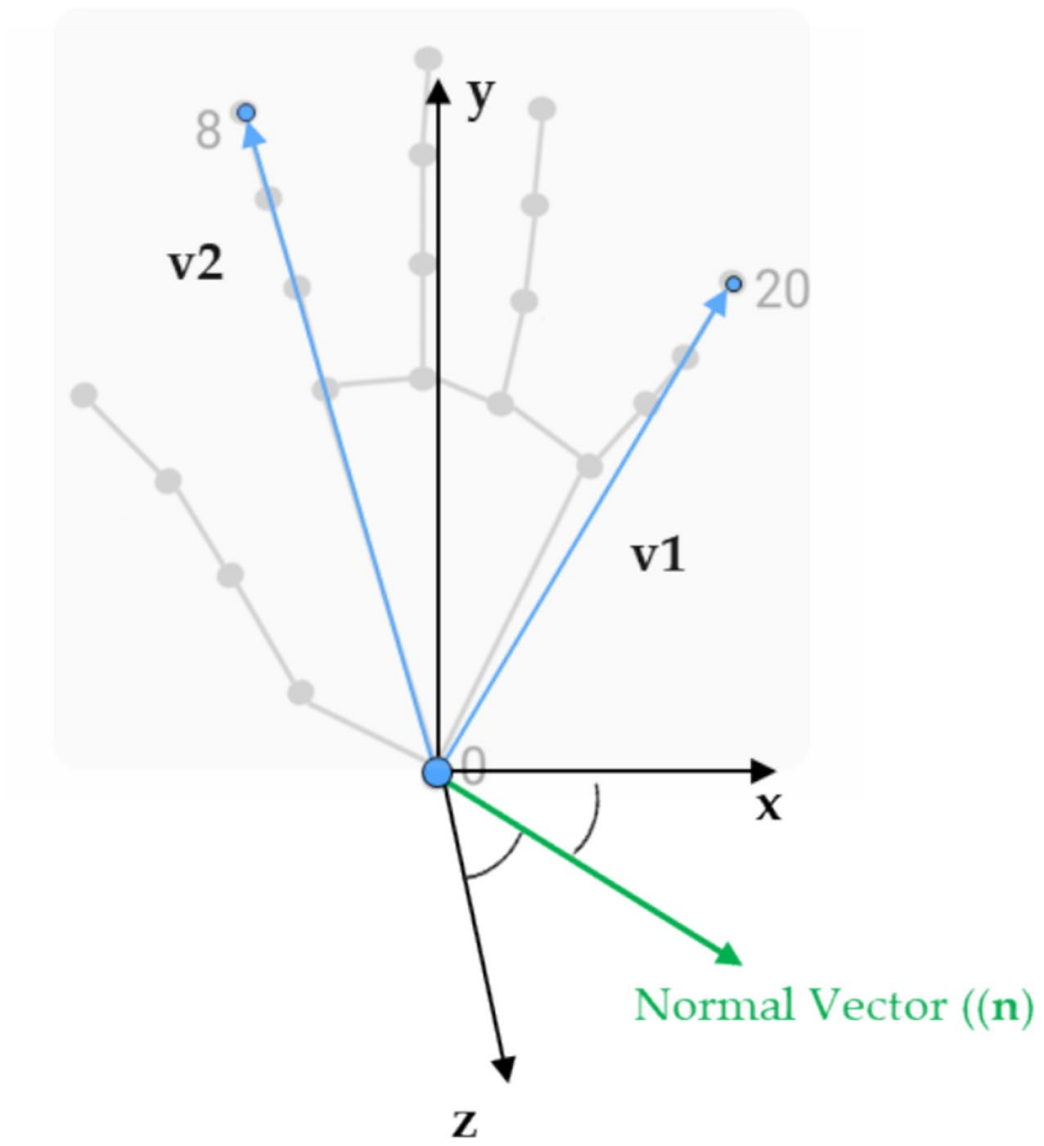
The 3D landmarks we used and the specific ones selected to calculate the palm normal and the angle that determined hand postures.

In more detail, the landmark detection framework provides the *X*, *Y*, and *Z* coordinates for each of the estimated anatomical landmarks. These 3D landmark points were used to mathematically calculate the locations of associated acupuncture points. Although MediaPipe predicts 21 hand landmarks (Figure 1a), accuracy constraints were encountered in projecting acupoints across different hand postures based on the literature guidelines. To overcome this, the hand postures were divided into four categories—front, inside, outside, and back views (Figure 3). To determine which posture the hand was in, three specific landmarks on the palm plane were selected (as shown in Figure 4), with one landmark serving as the reference point. Vectors were calculated from this reference point to the other two landmarks. Taking the cross product of these two vectors produced the palm’s 3D orientation vector. The angle between this palm vector and the global *Z*-axis was computed using the dot product. This angle measurement enabled classifying the hand into one of the four posture categories based on how much it diverged from the *Z*-axis orientation.

A similar methodology was utilized to model the face. The facial region was divided into three key postures—center, left, and right—in order to account for horizontal head rotation. Each of these three poses had a specific set of facial landmarks that were visible and could be detected. The proportional distances and angles between these landmarks (calculated using Equations 1 and 2) are then used to mathematically derive the predicted locations of associated acupoints.

**Figure 5:**
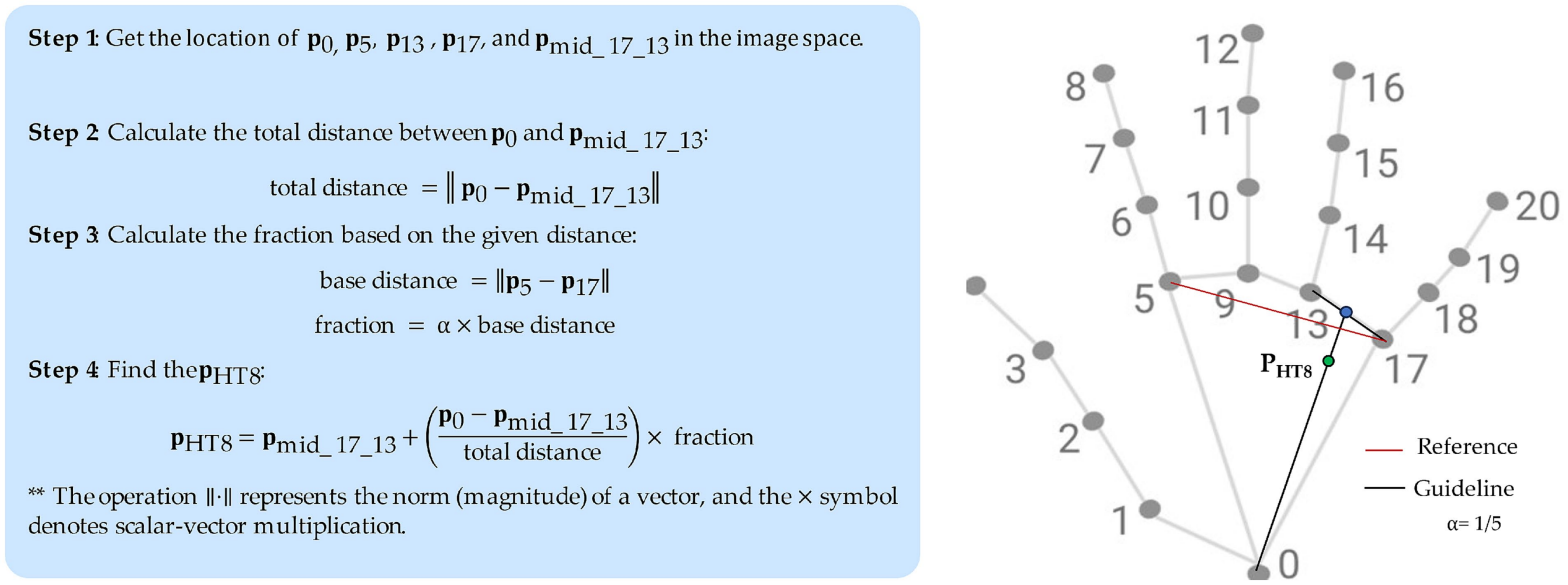
Example approach to localize an acupoint (HT8).

For example, the coordinates of the HT8 (Shaofu) acupoint, which is located on the palm of the hand, in the depression between the fourth and fifth metacarpal bones, proximal to the fifth metacarpophalangeal joint, is calculated in relation to the distance of landmarks 5 and 17 as shown in Figure 5. The Euclidean distance between these skeletal landmarks is first computed (base_distance). Next, based on a proportional measurement, the distance from HT8 to the point between landmarks 13 and 17 is calculated as 1/5 of that length (base_distance) toward landmark 0. Finally, we project the HT8 coordinates at the proper location along the palm.

(1) $$\begin{array}{c} d=∥\vec{\mathrm{Pi}}-\vec{Pj}∥=\sqrt{{\left(x_{i}-x_{j}\right)}^{2}+{\left(y_{i}-y_{j}\right)}^{2}} \end{array}$$

(2) $$\theta =\cos^{-1}\left(\frac{\vec{\mathrm{Pi}}\cdot \vec{Pj}}{\vec{∥\mathrm{Pi}}∥\cdot \vec{∥Pj∥}}\right)$$

Note that for facial acupuncture points, there are a greater number of anatomical landmarks (468) that can be used as reference points, which makes estimating the acupoint locations on the face easier compared to hand region with fewer identifiable landmarks. In addtion, there is no anatomical landmarks that can be reliably used as reference points for locating acupoints like LI11, LI10, and TE5 that located on forearm. Thus, this work focuses on acupoint localization for the hand given the greater challenges in accurately identifying forearm acupoints without established anatomical landmark provided by MediaPipe hand or pose estimation model.

In essence, classical acupuncture proportional methods are translated into computational geometric transformations in order to map key reference points on the body to known acupoint locations based on their relative positions. Further optimization of these formulaic projection techniques could enhance precision.

### 2.2 Data-driven pose estimation approach

**Figure 6:**
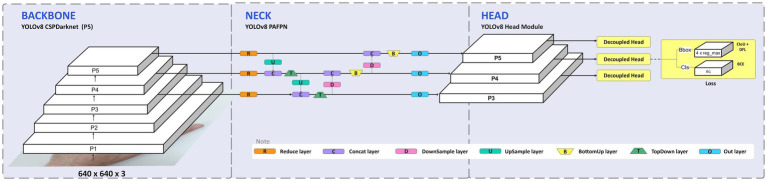
YOLOv8 architecture. The head can be decoupled to process objectness, classification, and regression tasks independently (Jocher et al., 2023; MMYOLO Contributors, 2023).

In addition to the proportional mapping approach, a data-driven deep learning model based on YOLOv8-pose was also developed to provide a comparative solution. Ultralytics released a version of the YOLO object detection model, providing state-of-the-art accuracy and speed for detection tasks. This version of YOLO has the same overall architecture (Figure 6) as previous versions, but it includes many enhancements compared to earlier iterations. It uses a new neural network design that combines feature pyramid network (FPN) and path aggregation network (PAN) architectures (Jocher et al., 2023). YOLO models are generally known for their computational efficiency and real-time performance, which aligns with the study’s goal of developing a real-time acupuncture point detection system.

YOLOv8 comes in 5 sizes and expands the capabilities beyond just detection to also include segmentation, pose estimation, tracking and classification. This new comprehensive computer vision system aims to provide an all-in-one solution for real-world applications (Terven et al., 2023). The YOLOv8 architecture leverages a convolutional neural network (Terven et al., 2023) to spatially localize and predict keypoints within the images. However, an official paper has yet to be released. We implement the code from the Ultralytics repository (Jocher et al., 2023).

#### 2.2.1 Dataset collection and preprocessing

**Figure 7:**
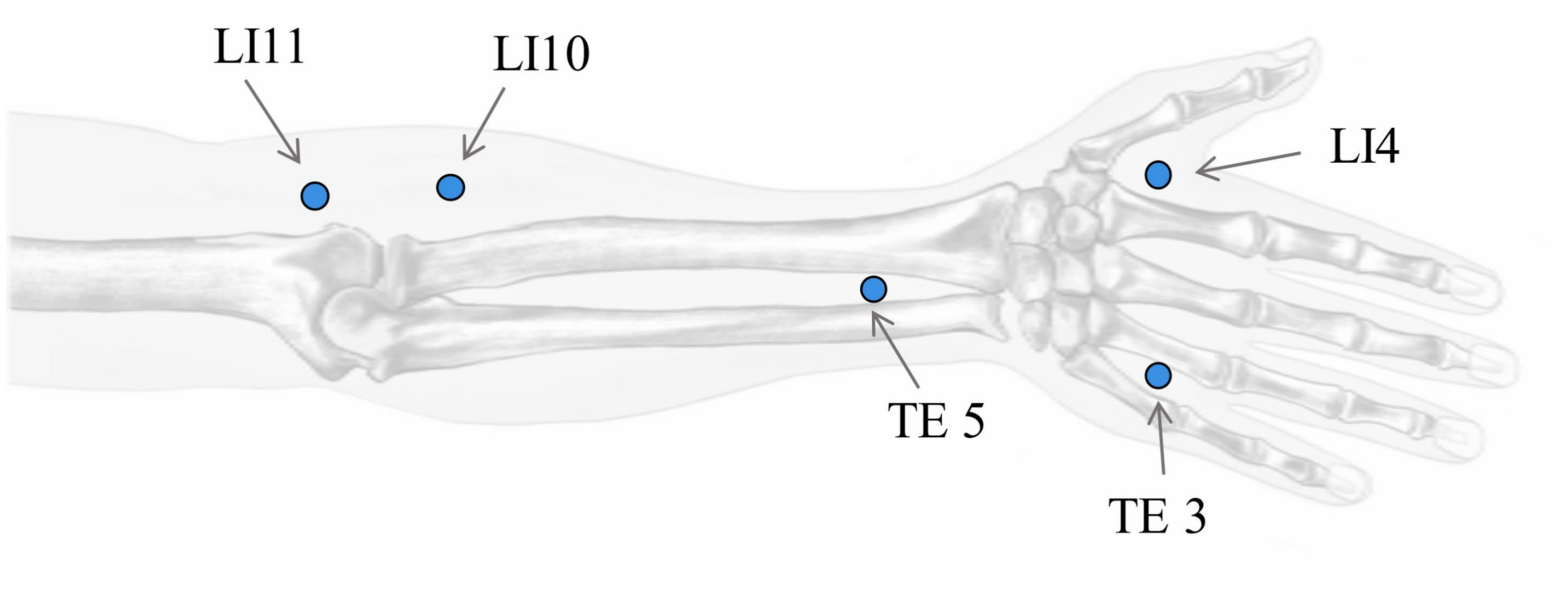
Location of acupoints on the hand. Shown are LI4 (Hegu), TE3 (Zhongzhu), TE5 (Waiguan), LI10 (Shousanli), and LI11 (Quchi). (Hand image reproduce from source National University of Korean Medicine, Graduate School of Korean Medicine, Meridian and Acupoint Studies Textbook Compilation Committee, 2020).

To train a real-time acupoint detection model, we collected a dataset comprising 5,997 acupoint-annotated images of arms at a resolution of 1,488 × 837 pixels. These images were sourced from 194 participants (49 male, 45 female, age range 19–68 years) at Pukyong National University and Dongshin University in South Korea, captured in a controlled laboratory environment with a white background. The dataset contains annotations marking five common acupoints on arm and hand—LI4 (Hegu), TE3 (Zhongzhu), TE5 (Waiguan), LI10 (Shousanli) and LI11 (Quchi)—localized according to the standard acupuncture point locations in the Western Pacific Region defined by the World Health Organization (Sulong and Randles, 2023) and verified by experts in oriental medicine (Figure 7). The annotations include bounding boxes around each arm and keypoint locations for the acupoints. The annotations were done using the COCO Annotator tool (Stefanics and Fox, 2022).

The data was then split into a training set of 5,392 images and a validation set of 605 images. A limitation of this dataset is that the arm poses and sizes are relatively uniform, lacking diversity. To help mitigate this, data augmentation techniques like rotation, scaling, and cropping applied on-the-fly to the training images to increase the diversity of the training data. Supplementary Figure S1 provides example input images from the dataset used by the model.

A limitation of this dataset is that the arm poses and sizes are relatively uniform, which may restrict the model’s ability to generalize to real-world scenarios with greater variability. To mitigate this, we employed data augmentation techniques during training. These techniques included rotation, scaling, and cropping, which were applied on-the-fly to the training images. This process introduced artificial variations in arm poses and sizes, enhancing the model’s exposure to a wider range of potential inputs. To minimize the impact of potential similarity between images from the same participant, we split the dataset into training and validation sets based on participants. The data was then split into a training set of 5,392 images and a validation set of 605 images. While these measures were taken to enhance the dataset’s diversity and mitigate potential biases, it is important to acknowledge that the validation process may still be limited by the relatively controlled nature of the data. Further evaluation on a more diverse dataset with a wider range of arm poses and sizes would be beneficial for a comprehensive assessment of the model’s generalizability. Supplementary Figure S1 provides example input images from the dataset used by the model.

#### 2.2.2 Model training and evaluation metrics

We decide to implement transfer learning and initialize our models with pre-trained weights from YOLOv8l-pose (large), which was pre-trained on human pose estimation using the COCO dataset. Evaluated on COCO Keypoints validation 2017 dataset, YOLOv8l-pose achieved an mAP_50–95_ of 67.6% and mAP_50_ of 90.0% with an image size of 640 pixels (Jocher et al., 2023). We then begin fine-tuning this base model on our custom dataset of acupoints on arm and hand images that as mentioned was split into a 90% training set and 10% validation set to adapt the model to specifically identify acupoints on hands. This transfer learning approach allows us to leverage the representations learned by the pre-trained YOLOv8-pose model to accelerate training on our more specialized acupoint detection task. In addition, it’s clear that a diverse dataset is crucial for deep learning models to make precise predictions. To enhance the performance of our pose estimation model, we implemented various data augmentation techniques. The augmentations we implemented were horizontal flipping of the images, rotation by varying degrees, mixup which combines samples through linear interpolation, and Mosaic augmentation that stitches together regions from multiple samples. These methods increased the diversity of our training data, which helped the model learn more robust features and improved accuracy.

For implementation, we utilized an Nvidia RTX 4090 GPU with 24GB RAM to efficiently train the acupoint detection model. Table 2 outlines the key training parameters used in the training process.

**Table 2:** Parameter settings for model training.

Parameters^a^	Values
Image size	640 × 640
#Epochs	300
#Batch-size	16
Initial learning rate	0.01
Main optimizer	SGD
Loss	CIoU_loss + DFL + Kobj_BCE + KIoU_loss^b^

a Full list of parameters used provided in Supplementary param.yaml file.

b IoU, intersection over union; CIoU, complete IoU; DFL, distribution focal loss; KBCE, keypoint objectness binary cross entropy; KIoU, Keypoint IoU (keypoint oks loss).

After model training was complete, several validation metrics were used to evaluate the performance of the acupoint detection model, including distance error (*E*), precision, recall, mean average precision (mAP), and object keypoint similarity (OKS), as outlined in Equations 3–7. The error *E* between the predicted acupoint position *P*_pred_ and the annotated ground truth acupoint position *P*_gt_ is defined as the Euclidean distance between them in the image space. The OKS metric specifically measures the similarity between predicted and ground truth keypoints, which is relevant for evaluating acupoint detection performance.

The specific formulas for calculating these metrics are:

(3) $$E=\;∥P_{\mathrm{pred}}-P_{gt}∥$$

(4) $$\mathrm{Precison}=\frac{TP}{TP+FP}\times 100\%$$

(5) $$\mathrm{Recall}=\frac{TP}{TP+FN}\times 100\%$$

(6) $$\mathrm{mAP}=\frac{\sum_{i=1}^{C}AP_{i}}{C}$$

(7) $$OKS=\exp\;\left(-\frac{d_{i}^{2}}{2s^{2}k_{i}^{2}}\right)$$

where: TP = true positives, FP = false positives, FN = false negatives, TN = true negatives, *C* = total number of categories, A*P_i_* = average precision for the *i*th category and mAP was calculated as the mean of average precision scores across all categories, to summarize the model’s overall precision. For each predicted keypoint, the OKS is calculated based on the Euclidean distance between the predicted and ground truth keypoint (*d_i_*), adjusted by the scale (*s*) which normalizes for object size, and a per-keypoint constant (*k*) that controls falloff. In our dataset, we used a constant *k* value of 0.02 for all keypoints. The OKS scores can then be averaged across keypoints and images to evaluate overall localization performance.

These metrics were computed on a validation set to evaluate the performance of the acupoint detection model after training.

## 3 Results

### 3.1 Landmark detection and proportional mapping approach

**Figure 8:**
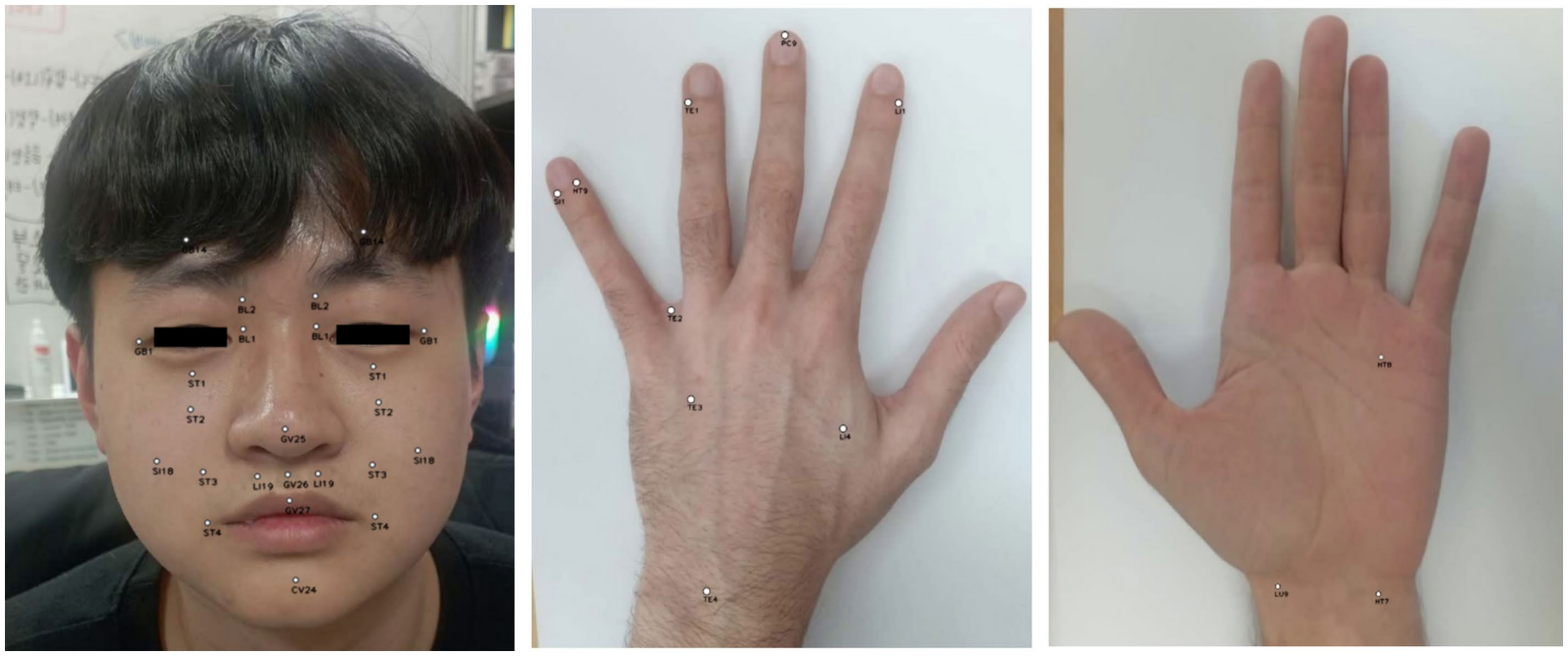
Example result of showing acupoints on the face and hand.

**Figure 9:**
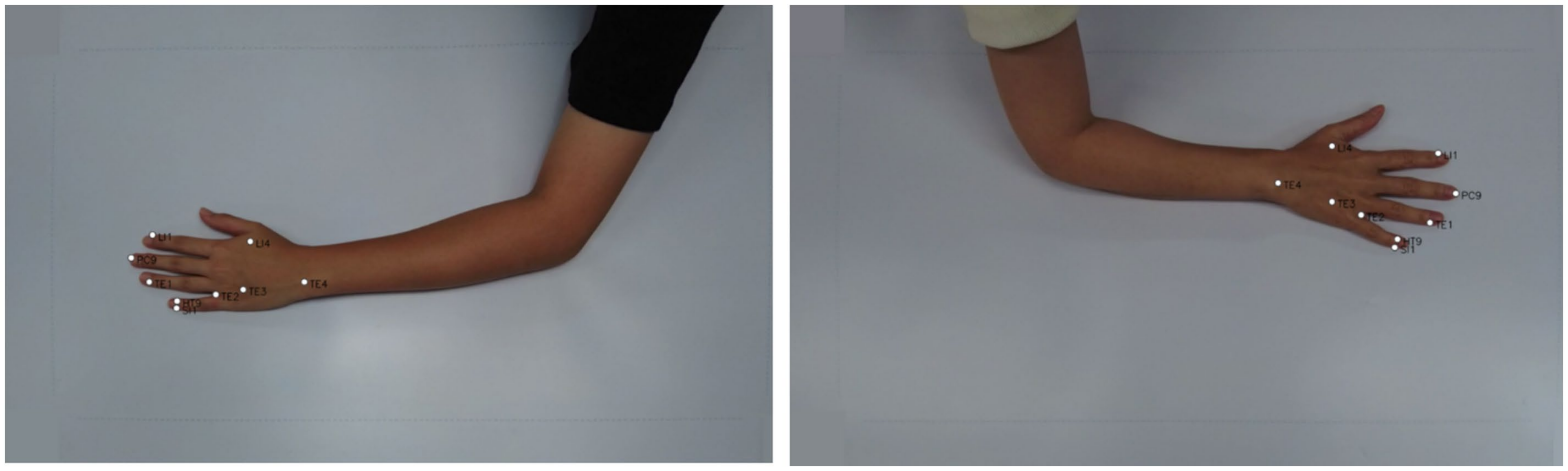
Exemplary images from dataset with landmark-based model outputs depicting acupoint locations on the back side of the hand, including LI4, TE1, TE2, TE3, LI1, PC9, SI1, HT9.

Through integrating principles of oriental medicine, literature references, and the MediaPipe framework, real-time performance in localizing 38 acupoints was accomplished in this study. Figures 8, 9 presents exemplary outcomes, demonstrating the proficiency of the proposed approach in detecting acupoints across various postures. Additionally, Supplementary Videos S1, S2 provide more extensive examples showcasing acupoint detection across a wide range of motions and poses.

We only evaluated the accuracy of our proposed model using a subset of 188 images from the larger dataset mentioned previously, which included 8 acupoints localization. These 188 images contain annotated acupuncture points that serve as ground truth landmarks. The images have annotations for 8 common acupoints on the back of the hand: LI4 (Hegu), TE3 (Zhongzhu), SI1 (Shaoze), HT9 (Shaochong), TE1 (Guanchong), PC9 (Zhongchong), LI1 (Shangyang), and TE2 (Erjian), These acupoints were selected for evaluation because of their frequent utilization in acupuncture therapy.

**Figure 10:**
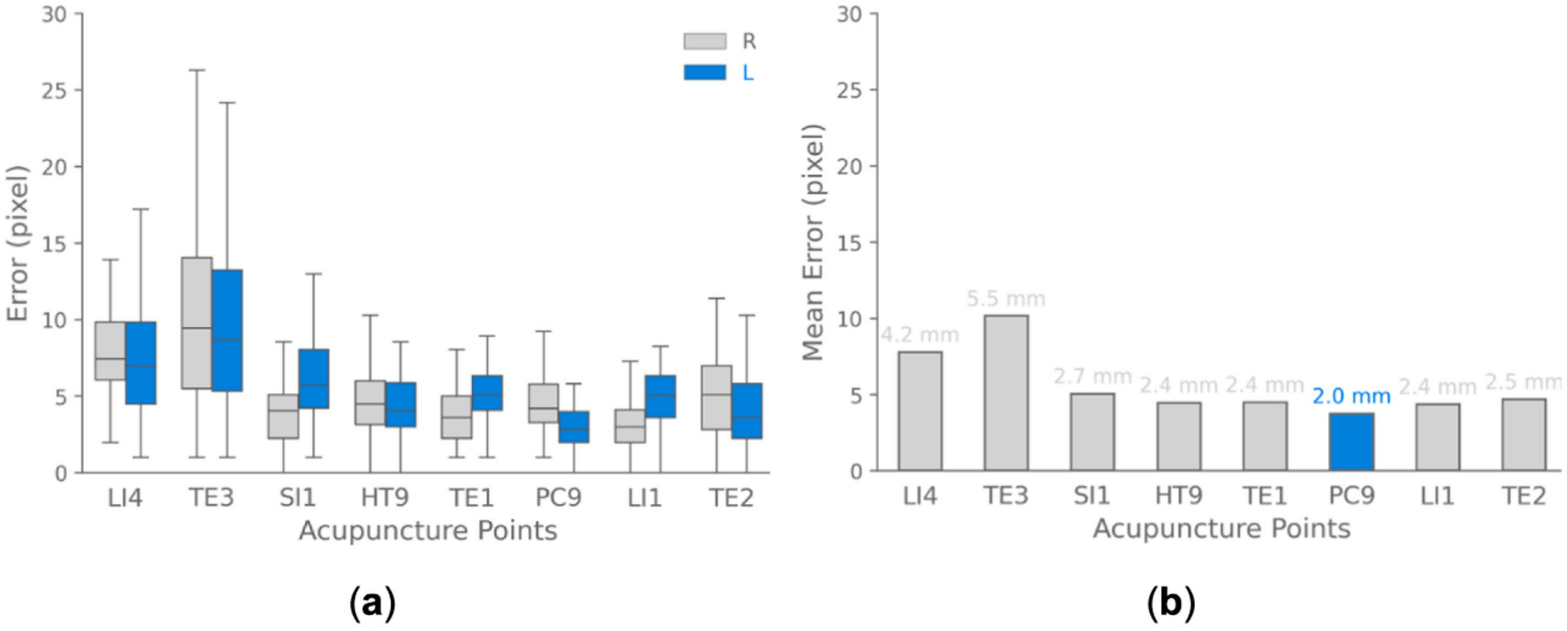
Acupoint localization accuracy landmark-based approach. **(a)** Boxplots depicting the distribution of Euclidean distance between predicted and ground truth acupoint locations for each evaluated acupoint separated for each hand (right and left). **(b)** Bar chart visualizing the mean of localization errors across different acupoints. The results demonstrate that the majority of points are localized with sub-centimeter accuracy.

Quantitative evaluation of model performance utilized the Euclidean distance metric (see Equation 3) to compute error between predicted and ground truth acupoint coordinates across all images of dataset. The average distance error achieved by this method was less than 10 pixels over all annotated landmarks (see Figure 10b). The low average distance error signifies that the predicted acupoint locations from the model closely correspond to the true anatomical locations demarcated by experienced practitioners.

We also analyzed the errors for localizing each individual acupoint location as shown in Figure 10. The box plots summarize the distribution of errors over all test images for each point. The median error varied based on the size and distinguishability of each point, ranging from ~4.0 pixels for the prominent PC9 acupoint to ~9.0 pixels for the TE3 acupoint. These results demonstrate that the model can detect acupoint near fingertips with high accuracy, localizing them within ~10 pixels for the majority of validation cases. These pixel-level errors correspond to approximately sub-centimeter accuracy in real-world coordinates.

To convert pixel errors to real-world coordinates, we used a simple calibration method. The images utilized for validation in this analysis were captured at a resolution of 1,488 × 837 pixels. A sheet with known horizontal length of approximately 80 cm was placed in the scene as a scale reference. This sheet spanned roughly 1,488 pixels horizontally across the image. Using the known real-world length and corresponding pixel length, we estimated a conversion factor of approximately 0.0537 cm per pixel. Utilizing this pixel-to-physical space calibration, the quantified pixel-level errors can be translated to real-world spatial errors with approximately sub-centimeter accuracy. With this calibration, for example, a pixel error of 10 pixels would translate to around 5.37 mm error in real-world coordinates.

To further assess the accuracy of predicted acupoint coordinates, we expanded our evaluation beyond the Euclidean distance metric. This comprehensive approach incorporates multiple statistical measures and visualizations, providing a more understanding of the model’s performance. In addition to the average distance error reported earlier, we calculated confidence intervals, and conducted statistical Kolmogorov–Smirnov tests to examine the significance of differences between predicted and actual coordinates as shown in Table 3. The test is a non-parametric statistical test that compares two distributions to see if they differ significantly. The mean distance between actual and predicted points is 5.58 pixels, with a narrow 95% confidence interval (5.38, 5.78), reflecting high accuracy. The Kolmogorov–Smirnov tests for both *X* and *Y* axes yield statistics of 0.010 and 0.012, respectively, with *p*-values of 1.000, suggesting that the error distributions are well-matched to the expected distributions.

**Table 3:** Summary of landmark-based approach model performance metrics and statistical tests.

Metric/test	Value (pixel)/statistic	95% CI/*p*-value
Mean distance	5.58	5.38–5.78
Kolmogorov–Smirnov test (*X*-axis)	Statistic = 0.010	*p* = 1.000
Kolmogorov–Smirnov test (*Y*-axis)	Statistic = 0.012	*p* = 1.000

**Figure 11:**
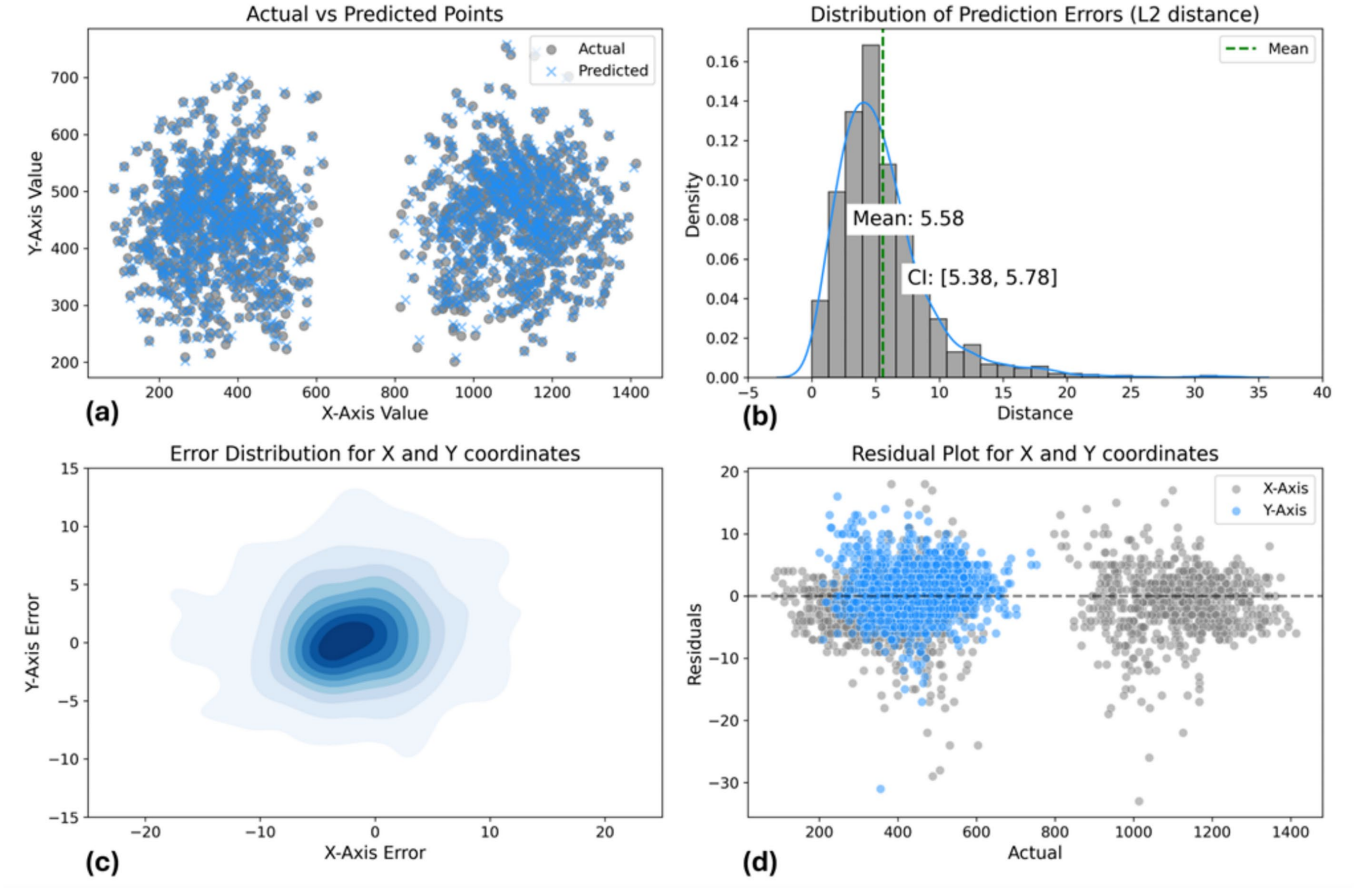
Landmark-based approach model performance evaluation. **(a)** Actual vs. predicted points scatter plot. **(b)** L2 distance error distribution [mean: 6.81 pixels, CI: (6.65, 6.98)]. **(c)** 2D error distribution for *X* and *Y* coordinates. **(d)** Residual plot showing prediction errors across coordinate range.

The model’s performance was assessed using multiple visualizations as shown in Figure 11. The scatter plot of actual vs. predicted points demonstrates a strong overall correspondence, with predicted points (blue) closely overlapping actual points (gray) across the coordinate space. The distribution of prediction errors reveals a mean L2 distance of 5.58 pixels, with a tight 95% confidence interval of (5.38, 5.78), indicating consistent accuracy. The error distribution for *X* and *Y* coordinates, visualized as a 2D density plot, shows a concentrated, symmetric pattern centered around zero, suggesting unbiased predictions. The residual plot further supports this, displaying a relatively even spread of errors around the zero line for both *X* and *Y* axes, with most residuals falling within ±10 pixels. Notably, there’s a clear separation in the residual values for the *X*-axis. This is due to the acupoints being predominantly associated with either the left or right hand, leading to distinct coordinate predictions based on hand position. Overall, these results demonstrate the model’s high precision in predicting spatial coordinates, with a small average error and well-suited error distributions across the prediction space.

### 3.2 Data-driven pose estimation approach

Data-driven pose estimation model achieves good accuracy for acupoint localization given the constraints of this dataset. The results validate the effectiveness of YOLOv8-pose for this medical imaging application and computer vision task.

**Figure 12:**
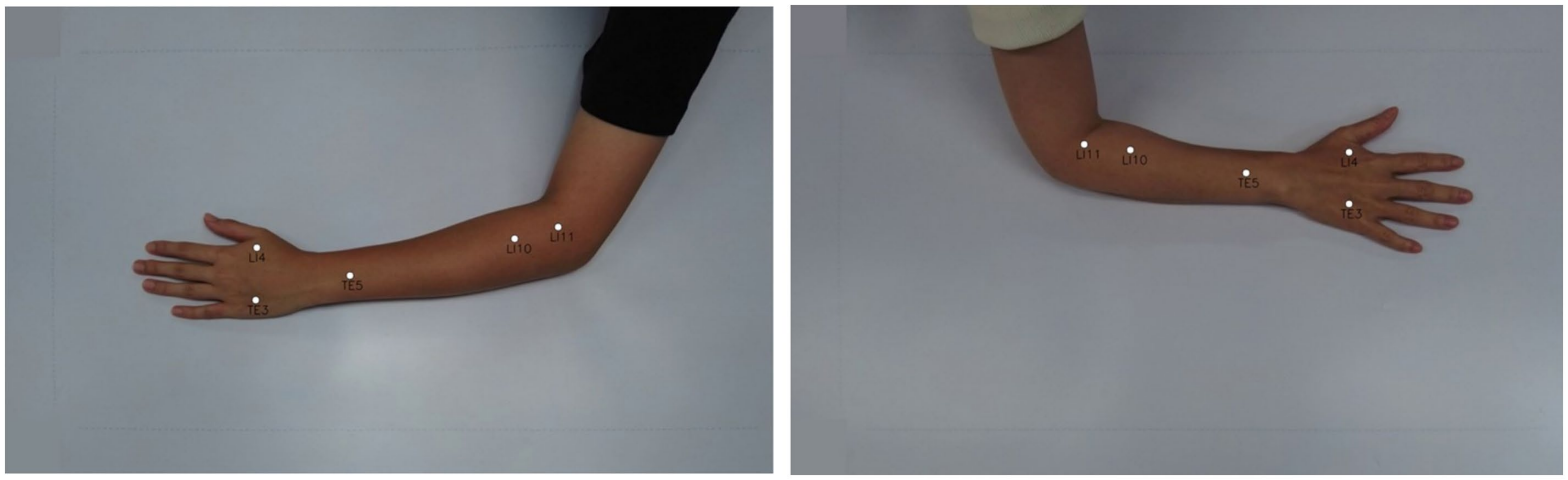
Acupoint localization accuracy of data-driven pose estimation approach. Shows the predicted acupoint locations from YOLOv8-pose.

Figure 12 visualizes two example outputs on the validation set for acupoint localization. More examples of validation batch results are shown in the Supplementary Figures S3, S4. Additionally, videos demonstrating the model’s acupoint localization on full motion sequences are provided in Supplementary Video S3. Qualitatively, YOLOv8 appears able to predict acupoint locations that closely match the ground truth in this controlled dataset. Some slight variations are visible upon close inspection, but overall YOLOv8-pose demonstrates acceptable performance for this acupoint localization task.

Quantitatively, YOLOv8-pose demonstrates high performance on acupoint localization as evidenced by high mean Average Precision (mAP) scores on the validation set. Specifically, it achieves an mAP at OKS 50% of 0.99 and 50–95 of 0.76 pose estimation. The complete quantitative results while training are presented in Supplementary Table S2. These high mAP values indicate that the model is able to accurately localize and identify acupoints in the validation images. Table 4 summarizes the model’s localization accuracy for each acupoint by reporting the Mean distance error in mm between the predicted and true acupoint positions. Note that to convert from pixels to mm, the pixel-to-mm conversion approach outlined in section 3.1 was used.

**Table 4:** Performance of YOLOv8-pose on the custom dataset of arm acupoints after 300 training epochs with an input size of 640 × 640 pixels.

Model	mAP^val^_50_ (pose)	mAP^val^_50–95_ (pose)	LI11 (mm)	LI10 (mm)	TE5 (mm)	LI4 (mm)	TE3 (mm)
YOLOV8l-pose (Pretraind)	0.99	0.76	3.68	3.97	4.14	2.93	3.82
			±(2.44)	±(2.65)	±(2.89)	±(2.59)	±(3.08)

Furthermore, we calculated confidence intervals and conducted statistical tests to evaluate differences between predicted and actual coordinates, as shown in Table 5. The mean distance between actual and predicted points is 6.81 pixels, with a 95% confidence interval of (6.65, 6.98), indicating good accuracy. The Kolmogorov–Smirnov tests for the *X* and *Y* axes yield statistics of 0.010 and 0.015, with *p*-values of 0.997 and 0.906, respectively, suggesting well-matched error distributions.

**Table 5:** Summary of data-driven pose estimation approach model performance metrics and statistical tests.

Metric/test	Value (pixel)/statistic	95% CI/*p*-value
Mean distance	6.81	6.65–6.98
Kolmogorov–Smirnov test (*X*-axis)	Statistic = 0.010	*p* = 0.997
Kolmogorov–Smirnov test (*Y*-axis)	Statistic = 0.015	*p* = 0.906

The results visualized in Supplementary Figure S2 show the loss and accuracy curves for both the training and validation data across training epochs. As demonstrated, the training and validation results showed that the YOLOv8-pose model for acupoint detection exhibited good convergence for this dataset. Specifically, the loss curve declined rapidly then flattened, indicating effective optimization. Meanwhile, the precision, recall, and mAP metrics increased quickly then stabilized, demonstrating model performance on the validation set.

**Figure 13:**
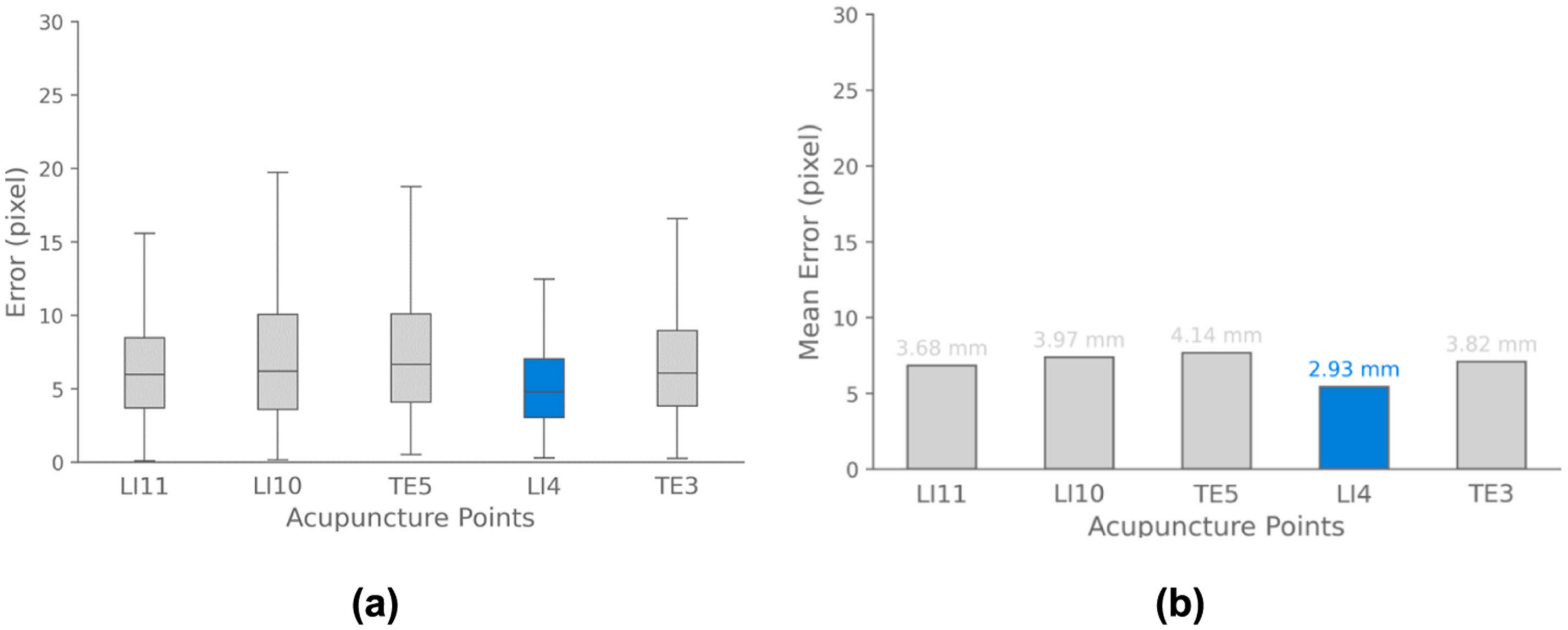
Acupoint localization accuracy for data-driven pose estimation approach. **(a)** Boxplots depicting the distribution of Euclidean distance errors between predicted and ground truth acupoint locations for each evaluated point. **(b)** Bar chart visualizing the mean localization errors across different acupoints. The results demonstrate that the majority of points are localized with sub-centimeter accuracy.

Additionally, Figure 13 illustrates the acupoint localization accuracy achieved by the YOLOv8-pose model. Boxplots in panel (a) show the distribution of Euclidean distance errors between predicted and ground truth locations for each acupoint. The bar chart in panel (b) visualizes the mean of localization errors.

**Figure 14:**
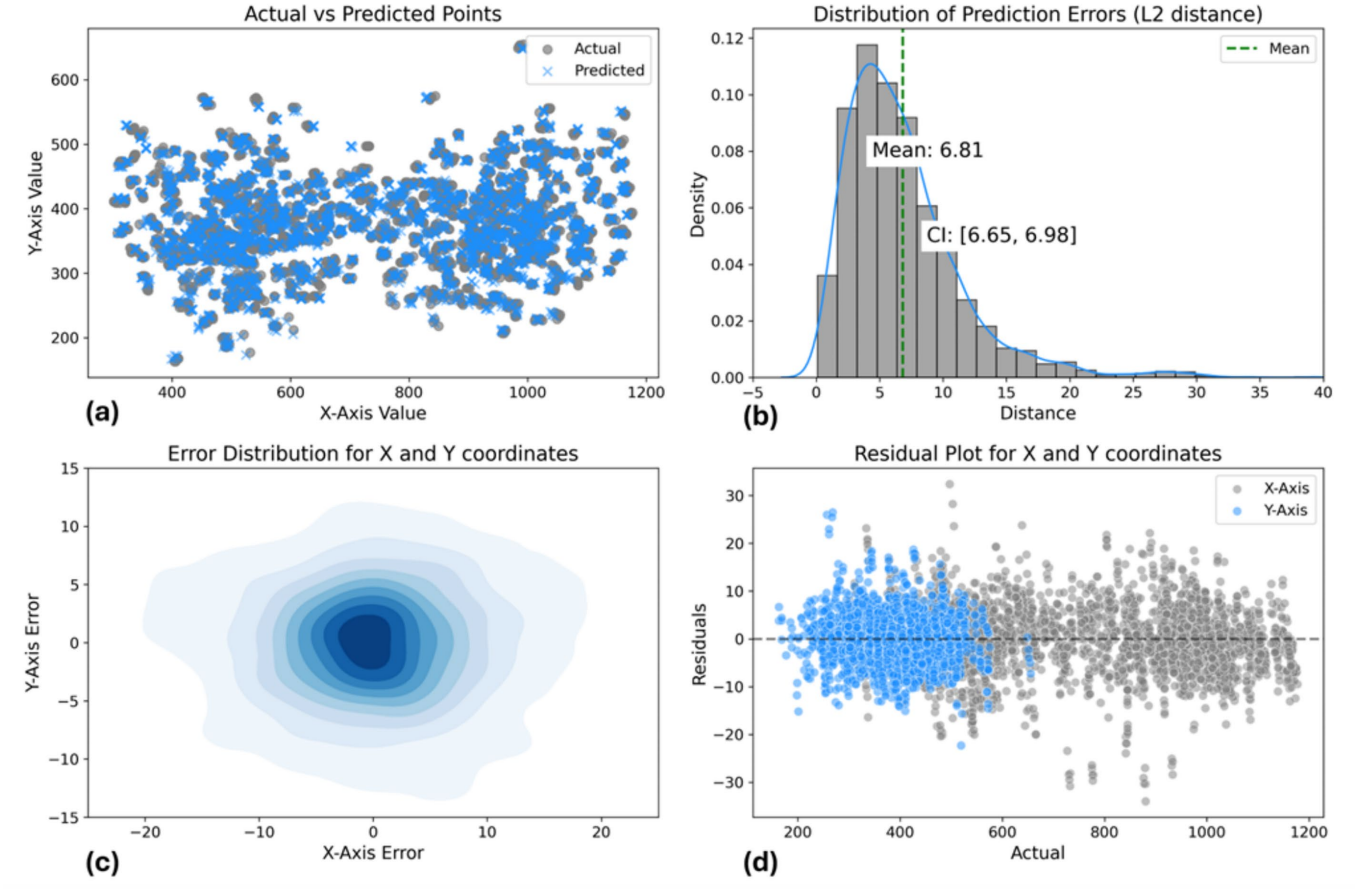
Data-driven pose estimation approach model performance evaluation: **(a)** Actual vs. predicted points scatter plot. **(b)** L2 distance error distribution [mean: 6.81 pixels, CI: (6.65, 6.98)]. **(c)** 2D error distribution for *X* and *Y* coordinates. **(d)** Residual plot showing prediction errors across coordinate range.

Regarding Figure 14, the scatter plot shows strong alignment between actual and predicted points, with a mean L2 error of 6.81 pixels and a 95% confidence interval of (6.65, 6.98), indicating consistent accuracy. The 2D density plot reveals a symmetric error distribution centered around zero, though some variability is observed. The residual plot highlights errors within ±10 pixels. Overall, the model performs well, but improvements could be made in reducing prediction variability and enhancing accuracy for points farther from the center.

### 3.3 Application development

To demonstrate the practical application of these models, a simple desktop application was developed using Tkinter, a Python library for creating graphical user interfaces. The application allows users to use a webcam feed for real-time acupoint localization. Upon camera activation, the landmark detection framework or the fine-tuned pose estimation model processes the input, and the identified acupoints are visualized on the screen.

**Figure 15:**
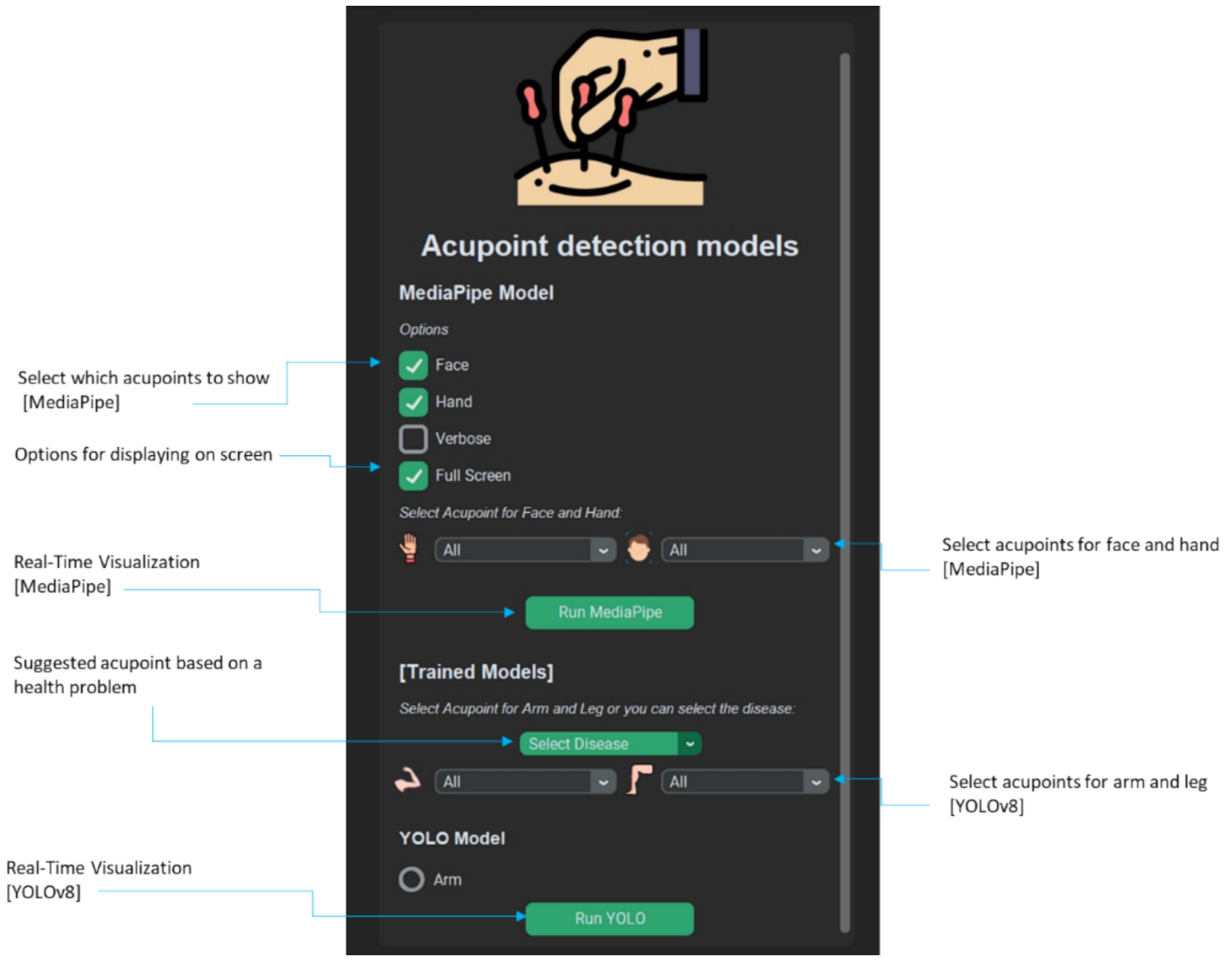
Interactive desktop application for real-time acupoint localization using pose estimation models on webcam feed (Additional usage examples provided in the Supplementary Figure S5 and Supplementary Video S4).

The application provides an intuitive interface for users (Figure 15). Further enhancing user experience, the application allows practitioners to choose specific acupoints of interest. Once the webcam is activated and the desired model and acupoints are selected, the system processes the live video feed. Identified acupoints are then dynamically overlaid onto the displayed image, providing practitioners with precise visual guidance. Looking ahead, we plan to expand the application’s capabilities to include acupoint localization on the legs, which are crucial for treating conditions affecting the lower body regions. Overall, this straightforward and user-friendly application showcases the potential of integrating AI-based acupoint localization into acupuncture treatments.

## 4 Discussion

This study investigated the feasibility of leveraging computer vision techniques to automate the localization of acupuncture points on the face and hands. Our study focused on these areas due to their frequent use in clinical practice and relative accessibility for imaging. These areas offer a high density of commonly used acupoints in a compact region, facilitating efficient data collection and analysis. Specifically, we explored two distinct approaches: Utilizing a real-time landmark detection framework to identify anatomical keypoints and map acupoint locations based on classical proportional measurement methods from acupuncture literature, and; fine-tuning a state-of-the-art pose estimation model on a custom dataset to directly predict acupoint coordinates through data-driven deep learning. Both methodologies demonstrated promising results in accurately identifying and visualizing acupuncture points in real-time settings.

The proposed landmark-based approach effectively detected anatomical keypoints to visually guide acupoint positioning. This could enable innovative acupuncture-assistive tools by providing practitioners with rapid on-screen guidance. A key advantage of our approach is the integration of the efficient framework, enabling real-time landmark detection and proportional mapping of acupoints across various hand and facial poses. As the architecture and training details of the MediaPipe models are proprietary, we cannot replicate the models directly through training our own model from scratch. Therefore, the methodology applies classical formulas to anatomically map and proportionally estimate acupoint locations. It can be easily adapted to different hand and face postures and allows for real-time visualization of acupoints. However, challenges remain in improving resilience to scale and rotation variances and using it for different body parts not accounted for in the original framework models. Qualitative assessment shows that accuracy depends heavily on the ability to reliably detect and track key anatomical landmarks. This can introduce some inaccuracies into the system’s final output. Furthermore, mathematical transformations may lack adaptability across heterogeneous populations. For a solution, transitioning to data-driven machine learning techniques could potentially address these limitations. The qualitative results show that the acupoints on the face have more invariance to transitional and rotational movement, which may be due to the higher number of landmarks in that region (468 keypoints). The quantitative results for hand dataset of 188 validation images reveal that the accuracy is higher for areas around the fingertips. This suggests it is easier for the model to locate these points compared to acupoints in the middle of the back of the hand.

In contrast to classical mapping techniques, data-driven deep learning approaches like the one employed by Sun et al. (2020, 2022) using U-Net and HRNet architectures can learn acupoint features directly from data. Our pose estimation model achieved acceptable acupoint localization accuracy (mAP at OKS 50–95% = 0.76, Mean error less than ~5 mm) in constrained arm dataset images for locating five acupoints. Compared to Sun et al.’s method that detected only 2 acupoints, our model localized 5 hand acupoints with high precision. Nevertheless, from Figure 10 it is evident that that the majority of the predicted acupoint locations for LI4, TE3, and LI11 exhibit high accuracy when approximately converted from pixel coordinates to physical distances based on the defined pixel-to-cm conversion factor. However, acupoints LI10 and TE5 exhibit higher localization errors compared to other acupoints. This suggests these two acupoints are more challenging for the model to precisely predict, perhaps due to greater variability in their location or appearance in the dataset. Further analysis of these acupoints may be needed to improve localization performance. While this model demonstrates acceptable performance for acupoint localization for controlled lab images, it may not generalize as well to more complex real-world hand images with cluttered background compared to MediaPipe that their model for hand landmarks trained on more than 30,000 images of hand. These initial results are encouraging, further evaluation is needed to determine how the model generalizes to real-world settings outside of the lab.

However, a significant limitation shared across studies in this scope, including ours, is the lack of large, publicly available datasets with expert-annotated acupoints. This hinders the ability to benchmark and compare the performance of different computer vision models specifically designed for acupuncture point detection, as we do not have access to comparable datasets or models (Sun et al., 2020; Sun et al., 2022; Chen et al., 2021). Finally, in this study the quantitative evaluation of facial acupoint localization was not addressed due to lack of a dataset of faces with known acupoint locations in this study, presenting an area for future investigation. Combining data-driven techniques with domain expertise in oriental medicine can pave the way for more advanced and integrative acupoint recognition systems.

Looking ahead, integrating the 3D capabilities of real-time landmark detection framework (including depth information) could enable 3D acupoint visualization and localization, a capability not explored in prior works. In contrast, the pose estimation currently lacks support for depth estimation, presenting an area for potential enhancement. Furthermore, investigating few-shot learning or domain adaptation techniques could enhance the generalization of data-driven models to handle real-world diversity beyond limited training data.

## 5 Conclusion

In conclusion, this study explored the potential of leveraging computer vision techniques for automating the localization of acupuncture points on the face and hands. Two distinct approaches were investigated: (1) utilizing a real-time landmark detection framework to map acupoints based on anatomical landmarks and proportional measurements, and (2) fine-tuning a state-of-the-art pose estimation model on a custom dataset for direct acupoint detection. The landmark-based system showed promising real-time acupoint visualization capabilities but had limitations due to potential landmark detection inaccuracies and rigid mapping formulas. The pose estimation model achieved sub-centimeter mean localization accuracy when fine-tuned on a controlled dataset but may face performance degradation in complex, real-world scenarios beyond the training dataset constraints. While both methodologies exhibit encouraging preliminary results, several challenges persist. These include the lack of large, diverse datasets for training and benchmarking acupoint detection models, as well as the need for further generalization and robustness to real-world variations. To address these challenges, our future work plans to curate a comprehensive dataset encompassing acupoints on legs and arms across a wide range of cluttered backgrounds and poses. Ultimately, the successful integration of computer vision and artificial intelligence into acupuncture practice holds immense potential for streamlining treatments, enhancing precision, and providing valuable assistive capabilities to practitioners. This work represents an important step towards realizing automated, technology-aided acupuncture, paving the way for further advancements in modernizing this ancient healing modality.

## Data Availability

The datasets presented in this article are not readily available because current agreements do not allow us to make this data publicly available. Requests to access the datasets should be directed to myunggi@pknu.ac.kr.

## Supplementary material

SUPPLEMENTARY VIDEO S1 Real-time facial acupoint detection using MediaPipe.

SUPPLEMENTARY VIDEO S2 Real-time hand acupoint detection using MediaPipe.

SUPPLEMENTARY VIDEO S3 Real-time hand acupoint detection using YOLOv8 pose estimation model.

SUPPLEMENTARY VIDEO S4 Real-time customized hand acupoint detection using Mediapipe through the GUI.
