# Supplementary material for: Real-time location of acupuncture points based on anatomical landmarks and pose estimation models
Source: Front Neurorobot. 2024 Nov 8;18:1484038. doi: 10.3389/fnbot.2024.1484038 (PMC11609928; doi:10.3389/fnbot.2024.1484038)
Supplement: SUPPLEMENTARY VIDEO S1 — Real-time facial acupoint detection using MediaPipe. [file Data_Sheet_1.ZIP › Supplementary/0-acupoints_data (Table S1).pdf]

TABLE S1

HELP	
b_d	base_distance<br>b_d = ‖p(#5) - p(#17)‖ for hand
f_cun	f_cun= 1/3 * b_d
t_d	Total_distance
p_?	coordinates of acupoint name
p (# $?)	coordinates of keypoint number $? from MediaPipe
p($fraction_{$1,$2})	coordinates for fraction distance between points $1 and $2
p(mid_{$1,$2})	coordinates for middle between points $1 and $2
d⊥(p$1, p$2, p$3, distance)	Perpendicular distance from the point $3 that is between points $1 and $2
‖.‖	the norm (magnitude) of a vector.
*	scalar-vector multiplication.
p(#?)_x	Represents the coordinates along the x-axis only
p(#?)_y	Represents the coordinates along the y-axis only
/	devision

Acupoint	Full Name	Meridian	Location	Clinical Impact	right hand / right side of face	left hand/ left side of face	
**Face**							
**CV-24**	Chengjiang	Conception Vessel	On the face, in the depression in the centreof the mentolabial sulcus.	Facial pain/paralysis - Bell's palsy, stroke affecting the face, mouth and/or tongue.<br>Dental pain - teeth and/or gums.<br>Speech issues - loss of voice, hoarseness, difficulty swallowing.<br>Dry mouth as seen in wasting and thirsting disorders (i.e. diabetes).	#center<br>p(#200)		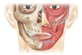
**BL-1**	Jingming	Bladder	On the face, in the depression between thesuperomedial parts of the inner canthus ofthe eye and the medial wall of the orbit.	Eye pain, strain, redness, swelling, itching, twitching, blurry vision, etc.<br>Myopia, color blindness, night blindness.<br>Glaucoma, optic nerve atrophy, cataracts.	p_BL1= p(#413)	p_BL1= p(#189)	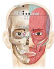
**BL-2**	Cuanzhu	Bladder	On the head, in the depression at the medialend of the eyebrow.	Local point for sinus congestion a/or headache.<br>Red, itchy, watery eyes - allergies.	p_BL2= p(#285)	p_BL12= p(#55)	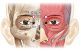
**GB-1**	Tongziliao	Gallbladder	On the head, in the depression, 0.5 B-cunlateral to the outer canthus of the eye.	Rising effecting the head - headache, eye problems, similar to Taiyang which is used more.<br>Local point for manifestations of heat - pain, inflammation, etc	p_GB1= p(p(#466)_x, p(#359)_y)	p_GB1= p(p(#226)_x, p(#130)_y)	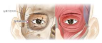
**GB-2**	Tinghui	Gallbladder	On the face, in the depression between theintertragic notch and the condylar processof the mandible	Ear issues, tinnius, otitis media, deafness.<br>Eliminate wind locally - TMJ disorder, facial paralysis, trigeminal neuralgia, toothache	p_GB2= p(#323)	p_GB2= p(#93)	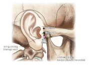
**GB-14**	Yangbai	Gallbladder	On the head, 1 B-cun superior to the eyebrow, directly superior to the centre of thepupil.	Frontal/temporal (ST/TH area) headaches.<br>Eye issues (direct needle towards eye) - redness, swelling, itching, twitching, etc	p_GB14= p(#299)	p_GB14= p(#69)	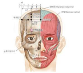
**GV-25**	Suliao	Governing Vessel	On the face, at the tip of the nose	Important point to restore or help maintain consciousness, for shock, loss of consciousness, to revive from drowning.<br>Rosacea, circulation issues in nose.<br>Nasal issues - rhinitis, polyps, discharge, inability or changes in olfactory sensations (smell).<br>May reduce alcohol intoxication (deadman).	#center<br>p_GV25= p(#4)		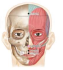
**GV-26**	Shuigou	Governing Vessel	On the face, at the midpoint of the philtrummidline.	Main point for acute low back sprain, helps relieve pain and restore motion, moves stagnation at the opposite end of the channel. After obtaining the Qi, have pateint bend then rotate the waist.<br>Nosebleed, nasal discharge issues, problems with olfactory sensations (smell).<br>Manic-depression and other strong shen disturbances, epilepsy (seizures).	#center<br>p_GV26= p(mid{p_LI19-right, p_LI19-left})		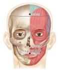
**GV-27**	Duiduan	Governing Vessel	On the face, at the midpoint of the tubercleof the upper lip.	Problems of the mouth and sinuses - tongue ulcers, bad breath (halitosis), pain a/or swelling of the gums, nosebleed, nasal congestion.<br>Psychological/Shen issues - manic depression, epilepsy, etc.	#center<br>p_GV27= p(#0)		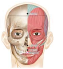
**LI-19**	Kouheliao	Large Intestine	On the face, at the same level as the midpoint of the philtrum, inferior to the lateralmargin of the nostril.	Sinus congestion.<br>Nasal polyps, sores.<br>Nosebleed	t_d = ‖p(#393) - p(#167)‖<br>p_LI19= p(#167) +(p(#393) - p(#167)) * (6/5*t_d)	t_d = ‖p(#393) - p(#167)‖<br>p_LI19= p(#393) +(p(#167) - p(#393)) * (6/5*t_d)	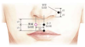
**LI-20**	Yingxiang	Large Intestine	On the face, in the nasolabial sulcus, at thesame level as the midpoint of lateral borderof the ala of the nose.	Loss of smell or taste, nasal discharge, any nose a/or sinus issues, nasal polyps, rhinitis, sinusitis, allergies.<br>Tong Ren/Tam Healing System: Sinus issues	p_LI20= p(#358)	p_LI20= p(#129)	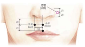
**SI-18**	Quanliao	Small Intestine	On the face, inferior to the zygomatic bone,in the depression directly inferior to theouter canthus of the eye.	Local point for facial disorders, Bell's palsy, trigeminal neuralgia, spasm, twitching of the eyelids, facial muscles, etc..<br>Upper jaw tootache.<br>Yellowing of the sclera.	t_d = ‖p(#411) - p(#425)‖<br>p_LI19= p(#411) +(p(#425) - p(#411)) * (1/3*t_d)	t_d = ‖p(#205) - p(#187)‖<br>p_LI19= p(#187) +(p(#205) - p(#187)) * (1/3*t_d)	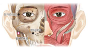
**ST-1**	Chengqi	Stomach	On the face, between the eyeball and theinfraorbital margin, directly inferior to thepupil.	Any eye issue, although ST 2 is a good alternative and arguably safer to needle.<br>Excessive lacrimation, redness, dryness, painful a/or itchy eyes/eyelids, twitching of the eyelids.<br>Visual disturbances, night blindness.	p_ST1= p(#450)	p_ST1= p(#230)	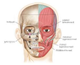
**ST-2**	Sibai	Stomach	On the face, in the infraorbital foramen.Below eye, lateral to ST-1	Any eye problem - red, painful and/or itchy eyes, excessive lacrimation, twitching of the eyelids.<br>Facial paralysis.<br>Good alternative to ST 1 and safer to needle for those not properly trained.	p_ST2= p(#330)	p_ST2= p(#101)	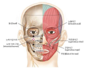
**ST-3**	Juliao	Stomach	On the face, directly inferior to the pupil, atthe same level as the inferior border of theala of the nose. Below eye, lateral to ST-2	Local point - Twitching eyelids, pain a/or swelling of the cheek, trigeminal neuralgia, toothache.<br>Deviation from stroke, bell's palsy, facial paralysis.<br>Swelling of the knee (Deadman text).	p_ST3= p(#426)	p_ST3= p(#206)	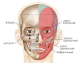
**ST-4**	Dicang	Stomach	On the face, in the depression between thesuperomedial parts of the inner canthus ofthe eye and the medial wall of the orbit	Facial pain - Bell's palsy, facial paralysis, trigeminal neuralgia, deviation of mouth from stroke, toothache.<br>Inability to close the eye, eye twitching, itching, night blindness.<br>Inability to eat.<br>May be helpful for atrophy and/or movement issues within the legs.	p_ST4= p(#287)	p_ST4= p(#57)	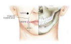
**ST-5**	Daying	Stomach	On the face, anterior to the angle of themandible, in the depression anterior to themasseter attachment, over the facial artery.	Local point - Pain a/or swelling of the cheek/jaw, neck pain, lockjaw, toothache.<br>Stiff tongue leading to speech problems, frequent yawning.<br>Inability to close eye.	t_d = ‖p(#365) - p(#367)‖<br>p_ST5= p(#365) +(p(#365) - p(#367)) * (1/2*t_d)	t_d = ‖p(#136) - p(#138)‖<br>p_ST5= p(#136) +(p(#138) - p(#136)) * (1/2*t_d)	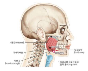
**ST-6**	Jiache	Stomach	On the face, one fingerbreadth (middlefinger) anterosuperior to the angle of themandible	Eliminate wind from the face - TMJ, toothache, Bell's palsy, twitching, facial pain/paralysis.<br>Loss of voice, deviation of mouth/face, lockjaw.	p_ST6= p(#288)	p_ST6= p(#58)	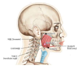
**ST-7**	Xiaguan	Stomach	On the face, in the depression between themidpoint of the inferior border of the zygomatic arch and the mandibular notch	TMJ, facial pain, lower jaw toothache.<br>Hearing issues, deafness, tinnitus, ear pain, ear discharge/infections.	t_d = ‖p(#366) - p(#454)‖<br>p_ST7= p(#366) +(p(#454) - p(#366)) * (1/3*t_d)	t_d = ‖p(#137) - p(#254)‖<br>p_ST7= p(#137) +(p(#254) - p(#137)) * (1/3*t_d)	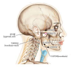
**TE-23**	Sizhukong	Triple Energizer	On the head, in the depression at the lateralend of the eyebrow.	Eye pain, redness, swelling, twitching, drooping eyelid.<br>Liver Wind or Liver Fire headache, combine with local points	p_TE23= p(#300)	p_TE23= p(#70)	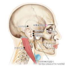
**Hand**							
**HT-7**	Shenmen	Heart	On the anteromedial aspect of the wrist,radial to the flexor carpi ulnaris tendon, onthe palmar wrist crease.Note: In the depression radial to the proximal border of the pisiform bone, on thepalmar wrist crease.	Tonify deficiencies of the HT Qi, Blood, Yin and Yang.<br>Emotional issues, especially those with related sleep or thinking manifestations - insomnia, muddled thinking.<br>Heart and Phlegm fire leading to insomnia, anxiety, mania.	HT7=d⊥(p(#0), p(#1), p(#0), -1/3*base_distance)	HT7=d⊥(p(#0), p(#1), p(#0), 1/3*base_distance)	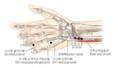

**HT-8**	Shaofu	Heart	On the palm of the hand, in the depressionbetween the fourth and fifth metacarpalbones, proximal to the fifth metacarpophalangeal joint.	Channel issues, sore throat, tongue stiffness, pain along the ulnar aspect of the arms.<br>Heart Qi Deficiency - palpitations, fear, hot palms.<br>Local point for arm pain, elbow pain, and/or pain in the pinky finger.	t_d = ‖p(mid_#13, #17) - p(#0)‖<br>**p_HT8**= p(mid_{#13, #17} +(p(#0) - p(mid_{#13, #17}) * 1/5*(b_d/t_d)	t_d = ‖p(mid_#13, #17) - p(#0)‖<br>**p_HT8**= p(mid_{#13, #17} +(p(#0) - p(mid_{#13, #17}) * 1/5*(b_d/t_d)	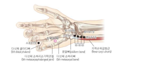
**HT-9**	Shaochong	Heart	On the little finger, radial to the distalphalanx, 0.1 f-cun proximal-lateral to theradial corner of the little fingernail, at theintersection of the vertical line of the radialborder of the nail and horizontal line of thebase of the little fingernail.	Restore consciousness - heart attack, stroke.<br>Emergency heart attack point - strong stimulation.<br>Fullness below the heart - stagnation, pain a/or distention in the HT area.	p_HT9=d⊥(p(#19), p(#20), p(2/3_{#19,#20}), -1/5 *f_cun)	p_HT9= d⊥(p(#19), p(#20), p(2/3_{#19,#20}), 1/5 *f_cun)	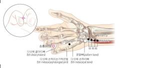
**LI-1**	Shangyang	Large Intestine	On the index finger, radial to the distalphalanx, 0.1 f-cun proximal-lateral to theradial corner of the index fingernail, at theintersection of the vertical line of the radialborder of the fingernail and the horizontalline of the base of the index fingernail.	pain, stagnation, especially at the opposite end of the channel, for eye and ear pain and inflammation, toothache.<br>Bleed for high fever, coma	p_LI1= d⊥(p(#7), p(#8), p(2/3_{#7,#8}), -1/4 *f_cun)	p_LI1= d⊥(p(#7), p(#8), p(2/3_{#7,#8}), 1/4 *f_cun)	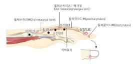
**LI-2**	Erjian	Large Intestine	On the index finger, in the depression distalto the radial side of the second metacarpophalangeal joint, at the border between thered and white flesh.	Shown to increase saliva production and to effect the areas of the brain involved in saliva production. Useful for dry mouth (xerostomia).<br>Sedation Point and Ying Spring Point, treats excess and clears heat from opposite end of the channel, for toothache, eye redness and pain, gum inflammation, sore throat.	p_LI2=p(1/6_{#5,#6})	p_LI2=p(1/6_{#5,#6})	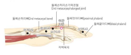
**LI-3**	Sanjian	Large Intestine	On the dorsum of the hand, in the depression radial and proximal to the secondmetacarpophalangeal joint.	Expel wind and heat particularly from the throat, teeth, mouth, face and eyes.<br>Diarrhea.	p_LI3=p(7/6_{#0,#5})	p_LI3=p(7/6_{#0,#5})	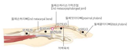
**LI-4**	Hegu	Large Intestine	On the dorsum of the hand, radial to themidpoint of the second metacarpal bone	Releases the exterior for wind-cold or wind-heat syndromes<br>Strengthens the wei qi, improves immunity<br>Headache, especially frontal a/or sinus (yangming area).<br>Chronic Pain	p_LI4=p(1/3_{p(3/5_{#1,#2}), #5})	p_LI4=p(1/3_{p(3/5_{#1,#2}), #5})	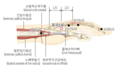
**LU-11**	Shaoshang	Lung	On the thumb, radial to the distal phalanx,0.1 f-cun proximal-lateral to the radialcorner of the thumb nail, at the intersectionof the vertical line of the radial border andthe horizontal line of the base of the thumbnail.	Extreme sore throat (may bleed point to reduce heat).<br>Revival point especially from wind-stroke.	t_d = ‖p(#3) - p(#4)‖<br>p_LU11= d⊥(p(#3), p(#4), p(2/3_{#3,#4}), 1/3*t_d)	t_d = ‖p(#3) - p(#4)‖<br>p_LU11= d⊥(p(#3), p(#4), p(2/3_{#3,#4}), 1/3*t_d)	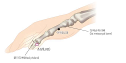
**LU-9**	Taiyuan	Lung	On the anterolateral aspect of the wrist,between the radial styloid process and thescaphoid bone, in the depression ulnar tothe abductor pollicis longus tendon.	Palpitations.<br>Wrist pain, arm pain along meridian.<br>Hui Meeting Point of the Vessls - circulation issues, clarifying an indescernible pulse (for diagnosis).<br>Main treatment point of Lung Sho pattern (with SP 3)	p_LU9= d⊥(p(#0), p(#1), p(3/5_{#1,#0}), 1/5*b_d)	p_LU9= d⊥(p(#0), p(#1), p(3/5_{#1,#0}), -1/5*b_d)	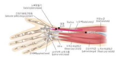
**PC-9**	Zhongchong	Pericardium	On the middle finger, at the centre of the tipof the middle finger.	Jing Well Point for clearing heat in the Heart.<br>Stiff tongue, speech disorders, especially those arising after a stroke.<br>Restoring consciousness.	t_d = ‖p(#11) - p(#12)‖<br>p_PC9= p(#11) +(p(#12) - p(#11)) * (5/4*t_d)	t_d = ‖p(#11) - p(#12)‖<br>p_PC9= p(#11) +(p(#12) - p(#11)) * (5/4*t_d)	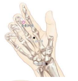
**SI-1**	Shaoze	Small Intestine	On the little finger, ulnar to the distalphalanx, 0.1 f-cun proximal-medial to theulnar corner of the little fingernail, at theintersection of the vertical line of ulnarborder of the nail and horizontal line of thebase of the little fingernail.	Breast disorders of any etiology, insufficient lactation, mastitis, breast abscess, cysts.<br>Local point for problems of the little finger and the course of the channel - pain and/or weakness of the arm and/or shoulder.	p_SI1=d⊥(p(#19), p(#20), p(2/3_{#19,#20}), 1/5 *f_cun)	p_SI1= d⊥(p(#19), p(#20), p(2/3_{#19,#20}), -1/5 *f_cun)	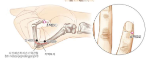
**SI-2**	Qiangu	Small Intestine	On the little finger, in the depression distalto the ulnar side of the fifth metacarpophalangeal joint, at the border between the redand white flesh.	Local point for pain, swelling, numbness of the fingers. As the water point it will remove heat - febrile disease, hot palms, darker urine.<br>Clear heat from the opposite end of the channel, tinnitus, headache, sore throat, neck, ear and/or eye pain and/or swelling.	p_SI2= p(1/5_{#17,#18})	p_SI2= p(1/5_{#17,#18})	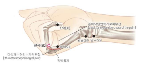
**SI-3**	Houxi	Small Intestine	On the dorsum of the hand, in the depression proximal to the ulnar side of the fifthmetacarpophalangeal joint, at the borderbetween the red and white flesh.	Clears heat and excess from the head, ears and eyes, eye redness, inflammation, visual dizziness, earache, tinnitus, sore throat.<br>Calms the spirit, anxiety, manic depression.	p_SI3=p(6/7_{#0,#17})	p_SI3=p(6/7_{#0,#17})	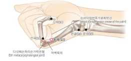
**SI-4**	Wangu	Small Intestine	On the posteromedial aspect of the wrist, inthe depression between the base of the fifthmetacarpal bone and the triquetrum bone,at the border between the red and whiteflesh.	Local point for shoulder, arm, hand and wrist problems particularly along the course of the meridian.<br>Useful point to treat problems with all fingers - contracture, pain, swelling, stiffness.<br>Headache, neck pain.<br>Jaundice.	p_SI4=p(1/15_{#0,#17})	p_SI4=p(1/15_{#0,#17})	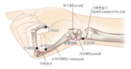
**TE-1**	Guanchong	Triple Energizer	On the ring finger, ulnar to the distalphalanx, 0.1 f-cun proximal to the ulnarcorner of the fingernail, at the intersectionof the vertical line of the ulnar side of thenail and the horizontal line of the base ofthe fingernail.	Ear Problems of any etiology such as excess tinnitus<br>Distal point for temporal headaches, shoulder a/or upper back pain, pain a/or inflammation in the throat.	p_TE1= d⊥(p(#15), p(#16), p(2/3_{#15,#16}), -1/4 *f_cun)	p_TE1= d⊥(p(#15), p(#16), p(2/3_{#15,#16}), -1/4 *f_cun)	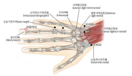
**TE-2**	Yemen	Triple Energizer	On the dorsum of the hand, in the depression superior to the web margin betweenthe ring and little fingers, at the borderbetween the red and white flesh.	Ear Problems of any etiology such as excess tinnitus<br>Distal point for temporal headaches, shoulder a/or upper back pain, pain a/or inflammation in the throat.	p_TE2= p(mid_{p(1/3_{#13, #14}), p(1/3_{#17,#18})	p_TE2= p(mid_{p(1/3_{#13, #14}), p(1/3_{#17,#18})	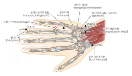
**TE-3**	Zhongzhu	Triple Energizer	On the dorsum of the hand, between the fourth and fifth metacarpal bones, in the depression proximal to the fourth metacarpophalangeal joint.	Ear Problems of any etiology such as excess tinnitus<br>Distal point for temporal headaches, shoulder a/or upper back pain, pain a/or inflammation in the throat.	t_d = ‖p(mid_#13, #17) - p(#0)‖<br>**p_TE3**= p(mid_{#13, #17} +(p(#0) - p(mid_{#13, #17}) * 1/3*(b_d/t_d)	t_d = ‖p(mid_#13, #17) - p(#0)‖<br>**p_TE3**= p(mid_{#13, #17} +(p(#0) - p(mid_{#13, #17}) * 1/3*(b_d/t_d)	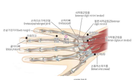
**TE-4**	Yangchi	Triple Energizer	On the posterior aspect of the wrist, in the depression ulnar to the extensor digitorum tendon, on the dorsal wrist crease.	Dorsal wrist issues such as strains, sprains or subluxations.<br>Wasting & thirsting disorder, regulates water metabolism, alleviates dry mouth a/or thirst.<br>Moxa to tonify the Yang of the body	p_TE4=p(5/4_{#1,#0})	p_TE4=p(5/4_{#1,#0})	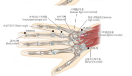

Refrence: Who standard Acupuncture point locations In the western pacific region

Refrence: Who standard Acupuncture point locations In the western pacific region

[illegible] (THE Acupoints of Acupuncture) [illegible] Republic of Korea, 2020

Reference: the Yin Yang House Website

https://yinyanghouse.com/
