# Supplementary material for: Real-time location of acupuncture points based on anatomical landmarks and pose estimation models
Source: Front Neurorobot. 2024 Nov 8;18:1484038. doi: 10.3389/fnbot.2024.1484038 (PMC11609928; doi:10.3389/fnbot.2024.1484038)
Supplement: SUPPLEMENTARY VIDEO S1 — Real-time facial acupoint detection using MediaPipe. [file Data_Sheet_1.ZIP › Supplementary/1-results_yolo (Table S2).pdf]

Table S2

epoch	train/box_loss	train/pose_loss	train/kobj_loss	train/cls_loss	train/dfl_loss	metrics/precision(B)	metrics/recall(B)	metrics/mAP50(B)	metrics/mAP50-95(B)	metrics/precision(P)	metrics/recall(P)	metrics/mAP50(P)	metrics/mAP50-95(P)	val/box_loss	val/pose_loss	val/kobj_loss	val/cls_loss	val/dfl_loss	lr/pg0	lr/pg1	lr/pg2
1	1.4961	10.129	0.65625	1.1285	1.957	0.51177	0.52397	0.37151	0.07673	0	0	0	0	3.292	9.5473	0.48782	1.1288	3.1374	0.0033234	0.0033234	0.0033234
2	1.2484	7.954	0.53113	0.80027	1.6892	0.65329	0.71322	0.67978	0.17017	0.08094	0.08595	0.02995	0.00513	3.0055	7.2806	0.4188	1.1979	3.0487	0.0066566	0.0066566	0.0066566
3	1.2658	7.3682	0.48983	0.85096	1.7158	0.32444	0.32231	0.20775	0.03857	0.17886	0.07934	0.04222	0.00714	3.8528	7.1694	0.40501	1.3804	3.9282	0.009989	0.009989	0.009989
4	1.3092	6.9966	0.44357	0.94789	1.7499	0.60806	0.65785	0.56526	0.13557	0.11874	0.04463	0.01819	0.00443	2.4325	7.2353	0.3769	2.2718	2.4611	0.0099976	0.0099976	0.0099976
5	1.277	6.1334	0.39442	0.93264	1.705	0.33687	0.40331	0.19499	0.03893	0.33058	0.36033	0.20255	0.04511	3.4404	5.2336	0.30543	2.1835	3.3907	0.0099976	0.0099976	0.0099976
6	1.2329	5.6212	0.36499	0.88548	1.6575	0.35702	0.36529	0.21135	0.03989	0.63952	0.65124	0.55181	0.16491	3.704	3.9278	0.28899	1.3189	3.9268	0.0099957	0.0099957	0.0099957
7	1.2096	5.1913	0.34343	0.86355	1.6309	0.5235	0.53223	0.37167	0.07676	0.58334	0.58678	0.40142	0.09469	3.2733	3.8236	0.27246	1.3794	3.5111	0.0099932	0.0099932	0.0099932
8	1.1923	4.8803	0.3256	0.83462	1.6132	0.31039	0.30413	0.18014	0.02863	0.68744	0.6876	0.50437	0.12594	3.8749	3.5754	0.25491	1.6745	4.312	0.0099902	0.0099902	0.0099902
9	1.1754	4.6352	0.31345	0.81995	1.5943	0.5739	0.6	0.52614	0.12677	0.71205	0.7157	0.60611	0.15057	2.7507	4.2677	0.24687	1.5555	3.1851	0.0099867	0.0099867	0.0099867
10	1.1518	4.3499	0.30224	0.78908	1.5711	0.63452	0.67273	0.5969	0.12304	0.71928	0.73058	0.61056	0.15578	3.0766	3.5669	0.23916	1.2311	3.1639	0.0099826	0.0099826	0.0099826
11	1.1428	4.2565	0.29242	0.78615	1.567	0.58977	0.59645	0.45298	0.09587	0.66754	0.66777	0.55047	0.17223	3.1937	3.3498	0.22623	1.061	3.4429	0.009978	0.009978	0.009978
12	1.1362	4.0752	0.27993	0.77439	1.5555	0.79504	0.80786	0.71506	0.21336	0.85366	0.85813	0.80468	0.30635	2.5904	2.7905	0.20175	0.98142	2.7477	0.0099729	0.0099729	0.0099729
13	1.1348	4.0374	0.27266	0.77122	1.5491	0.68411	0.6843	0.56772	0.1144	0.77169	0.7719	0.61712	0.17567	3.0807	3.5336	0.19458	1.1208	3.3243	0.0099672	0.0099672	0.0099672
14	1.1099	3.811	0.26029	0.75472	1.5309	0.69661	0.72868	0.59632	0.14308	0.77616	0.79008	0.67898	0.20847	2.7708	3.3333	0.17817	1.0704	2.8789	0.009961	0.009961	0.009961
15	1.105	3.7224	0.25763	0.73735	1.5264	0.54491	0.55372	0.37343	0.08158	0.87999	0.88099	0.8097	0.26981	3.305	2.7191	0.1794	1.6581	3.6558	0.0099542	0.0099542	0.0099542
16	1.1089	3.6662	0.24823	0.75162	1.5325	0.78199	0.82479	0.73671	0.19569	0.83834	0.85715	0.75153	0.23478	2.5886	3.0222	0.17369	1.0249	2.9916	0.0099469	0.0099469	0.0099469
17	1.1092	3.5556	0.24402	0.74708	1.5272	0.66955	0.6843	0.57215	0.13477	0.85326	0.85785	0.79706	0.25707	2.8862	3.4019	0.16249	1.256	3.1759	0.0099391	0.0099391	0.0099391
18	1.1034	3.5845	0.23803	0.74038	1.5247	0.82798	0.8281	0.75089	0.1742	0.89573	0.89587	0.81705	0.29525	2.7616	2.4126	0.1399	0.89725	2.9201	0.0099307	0.0099307	0.0099307
19	1.084	3.5485	0.23244	0.72443	1.5039	0.76705	0.79461	0.66356	0.15541	0.95584	0.96529	0.9451	0.38541	2.7729	2.2309	0.14914	1.0428	3.0565	0.0099218	0.0099218	0.0099218
20	1.069	3.3813	0.22777	0.71348	1.4897	0.79874	0.84624	0.80579	0.25307	0.83989	0.85455	0.75045	0.28266	2.5506	2.9395	0.13709	1.0547	2.763	0.0099123	0.0099123	0.0099123
21	1.065	3.3848	0.22244	0.70546	1.4914	0.77672	0.77686	0.67917	0.16195	0.92545	0.92562	0.88812	0.33006	2.8202	2.6938	0.12995	0.96021	3.1761	0.0099023	0.0099023	0.0099023
22	1.0575	3.2895	0.21579	0.69626	1.4879	0.77759	0.78678	0.70817	0.1908	0.95639	0.95702	0.93688	0.41516	2.6868	2.2933	0.13416	0.95058	2.9712	0.0098918	0.0098918	0.0098918
23	1.0577	3.2339	0.21408	0.69204	1.4844	0.5437	0.5438	0.37206	0.07196	0.89571	0.89587	0.81266	0.32329	3.2627	2.7979	0.13196	1.4434	3.8482	0.0098808	0.0098808	0.0098808
24	1.0546	3.2009	0.20851	0.69913	1.485	0.62637	0.62645	0.45734	0.0949	0.91724	0.91736	0.87062	0.31015	3.1567	2.4301	0.11746	1.0546	3.8006	0.0098692	0.0098692	0.0098692
25	1.0411	3.1997	0.2093	0.68074	1.4704	0.77082	0.80165	0.70346	0.18784	0.90486	0.92562	0.89045	0.36648	2.6169	2.3701	0.10808	0.94803	2.8704	0.0098571	0.0098571	0.0098571
26	1.0372	3.1226	0.20421	0.66637	1.4648	0.75204	0.75207	0.67894	0.15838	0.95691	0.95702	0.92087	0.37983	2.9059	2.2855	0.11736	0.90133	3.3849	0.0098445	0.0098445	0.0098445
27	1.0233	3.0604	0.19739	0.65223	1.4498	0.6148	0.61488	0.4834	0.12182	0.92054	0.92066	0.8802	0.37876	3.1572	2.2995	0.10355	0.94948	3.7568	0.0098313	0.0098313	0.0098313
28	1.0188	3.014	0.19273	0.65914	1.4522	0.77207	0.80826	0.70656	0.19646	0.93197	0.94876	0.92803	0.39323	2.6181	2.2857	0.11771	0.96675	3.0595	0.0098177	0.0098177	0.0098177
29	1.0184	2.9889	0.19085	0.65169	1.448	0.75574	0.75689	0.65567	0.16534	0.95194	0.95207	0.92154	0.42455	2.8675	2.1053	0.10012	0.90554	3.2778	0.0098035	0.0098035	0.0098035
30	1.0183	2.9848	0.18731	0.65603	1.4524	0.77013	0.78633	0.68955	0.18481	0.93121	0.9405	0.9073	0.37367	2.702	2.2716	0.0996	0.87879	3.1579	0.0097887	0.0097887	0.0097887
31	1.0026	2.8872	0.18544	0.64173	1.4331	0.79021	0.80938	0.7154	0.17552	0.95159	0.95868	0.93393	0.41371	2.7258	2.2261	0.08853	0.9023	3.2822	0.0097735	0.0097735	0.0097735
32	1.0045	2.8617	0.18326	0.64832	1.4405	0.77756	0.80311	0.68071	0.17637	0.94229	0.94545	0.91134	0.40181	2.6798	2.0313	0.09263	0.90018	3.1339	0.0097577	0.0097577	0.0097577
33	1.004	2.8701	0.18076	0.64132	1.4374	0.73687	0.74524	0.61211	0.15605	0.95833	0.95868	0.93304	0.41937	2.7635	2.1551	0.09482	0.94091	3.0715	0.0097415	0.0097415	0.0097415
34	0.99955	2.8143	0.17939	0.63429	1.4298	0.82252	0.85124	0.79403	0.25261	0.93904	0.97025	0.9594	0.48965	2.461	1.9487	0.0829	0.91956	2.7333	0.0097247	0.0097247	0.0097247
35	0.99419	2.806	0.17419	0.63135	1.4261	0.59661	0.60165	0.41503	0.11088	0.96582	0.9719	0.96391	0.47764	3.0129	1.9138	0.08667	1.0363	3.3729	0.0097074	0.0097074	0.0097074
36	0.99007	2.7809	0.17301	0.62381	1.4228	0.78212	0.80992	0.65943	0.16905	0.96995	0.97025	0.95016	0.4666	2.6962	1.8805	0.08437	0.95205	3.2817	0.0096895	0.0096895	0.0096895
37	0.98027	2.7918	0.17024	0.62464	1.4148	0.80606	0.82975	0.74157	0.20348	0.96007	0.96033	0.93876	0.44839	2.6469	2.012	0.10326	0.81095	2.9536	0.0096712	0.0096712	0.0096712
38	0.97844	2.7491	0.16999	0.62462	1.4191	0.75527	0.75537	0.69347	0.20049	0.94532	0.94545	0.91507	0.46037	2.74	1.9681	0.08124	0.8562	3.2655	0.0096524	0.0096524	0.0096524
39	0.9718	2.6894	0.16666	0.61659	1.4114	0.78139	0.80992	0.68255	0.1775	0.93194	0.96033	0.93973	0.43412	2.5238	2.0293	0.08125	0.9299	2.8282	0.0096331	0.0096331	0.0096331
40	0.97715	2.7082	0.16514	0.61851	1.4097	0.76699	0.79437	0.65236	0.17295	0.9538	0.96198	0.94038	0.40732	2.6077	1.9969	0.08192	0.91023	3.1411	0.0096132	0.0096132	0.0096132
41	0.96604	2.6682	0.16426	0.61281	1.4049	0.75192	0.75207	0.62541	0.15522	0.96511	0.96529	0.94317	0.45435	2.8515	1.9756	0.07218	0.90204	3.4332	0.0095929	0.0095929	0.0095929
42	0.97696	2.6978	0.16469	0.6192	1.4174	0.8346	0.83471	0.80103	0.24078	0.97178	0.9719	0.96371	0.4929	2.6576	1.9855	0.07763	0.82632	3.0894	0.0095721	0.0095721	0.0095721
43	0.96702	2.678	0.16175	0.60823	1.4063	0.7593	0.80496	0.65223	0.17842	0.96003	0.9686	0.95285	0.44824	2.561	1.8982	0.06666	0.90878	3.138	0.0095507	0.0095507	0.0095507
44	0.95255	2.6089	0.1616	0.61168	1.3972	0.78743	0.80823	0.73615	0.24366	0.96731	0.97025	0.95722	0.51859	2.5252	1.8356	0.07523	0.86871	3.125	0.0095289	0.0095289	0.0095289
45	0.95886	2.5879	0.15833	0.60059	1.3971	0.76491	0.77444	0.70389	0.21091	0.9734	0.97355	0.96678	0.47171	2.6988	2.0108	0.06658	0.85166	3.0485	0.0095066	0.0095066	0.0095066
46	0.94699	2.5267	0.15482	0.5908	1.3874	0.80361	0.80484	0.71406	0.20874	0.97674	0.97686	0.96445	0.50192	2.5914	1.9596	0.07788	0.8676	3.1936	0.0094838	0.0094838	0.0094838
47	0.95237	2.5524	0.15477	0.59399	1.3917	0.66936	0.66942	0.50509	0.12193	0.98005	0.98017	0.96968	0.49188	2.9354	1.8388	0.07205	0.94276	3.5311	0.0094605	0.0094605	0.0094605
48	0.94371	2.4846	0.15465	0.58602	1.3869	0.80935	0.83802	0.76983	0.25277	0.98286	0.98512	0.97809	0.5315	2.436	1.7663	0.06755	0.91678	2.8736	0.0094367	0.0094367	0.0094367
49	0.94074	2.4794	0.15237	0.58171	1.3837	0.78894	0.79083	0.74787	0.22984	0.98335	0.98347	0.97514	0.53151	2.6951	1.8255	0.06992	0.77833	3.2265	0.0094125	0.0094125	0.0094125
50	0.94127	2.4674	0.15114	0.58608	1.384	0.77015	0.77025	0.71521	0.25593	0.97839	0.97851	0.96357	0.48129	2.5376	1.9895	0.09343	0.8524	3.1129	0.0093877	0.0093877	0.0093877
51	0.94202	2.478	0.14993	0.58297	1.3785	0.79825	0.79835	0.73304	0.20096	0.97675	0.97686	0.96973	0.51475	2.6973	1.7801	0.06824	0.79341	3.3609	0.0093625	0.0093625	0.0093625
52	0.93778	2.4556	0.15037	0.58257	1.3758	0.77135	0.77509	0.63089	0.17154	0.96628	0.9686	0.94681	0.47994	2.6221	1.8335	0.06175	0.87695	3.3306	0.0093368	0.0093368	0.0093368
53	0.9322	2.4331	0.14769	0.58204	1.3745	0.72719	0.72727	0.62191	0.19244	0.98667	0.98678	0.98127	0.51287	2.813	1.7971	0.06227	0.81911	3.4426	0.0093107	0.0093107	0.0093107
54	0.9286	2.4146	0.14529	0.58001	1.3739	0.77839	0.77851	0.68641	0.19937	0.9701	0.97025	0.96324	0.48585	2.7315	1.8818	0.0701	0.86232	3.5337	0.0092841	0.0092841	0.0092841
55	0.92649	2.3812	0.14417	0.57389	1.3722	0.78502	0.78512	0.67989	0.15997	0.98334	0.98347	0.97234	0.51192	2.7927	1.7664	0.06872	0.88664	3.3523	0.009257	0.009257	0.009257
56	0.93067	2.3882	0.14537	0.58033	1.3762	0.74207	0.74215	0.65222	0.18273	0.98667	0.98678	0.98036	0.55542	2.7486	1.6471	0.05574	0.82822	3.3922	0.0092294	0.0092294	0.0092294
57	0.9283	2.3884	0.14389	0.56866	1.3659	0.72389	0.72397	0.61825	0.16714	0.98171	0.98182	0.97555	0.54128	2.9091	1.6749	0.04768	0.89548	3.7014	0.0092014	0.0092014	0.0092014
58	0.93594	2.3686	0.14419	0.58244	1.3727	0.75523	0.75537	0.66287	0.20025	0.97998	0.98017	0.97346	0.54927	2.8179	1.7054	0.05943	0.87807	3.4998	0.009173	0.009173	0.009173
59	0.91995	2.3862	0.14369	0.57063	1.3626	0.71062	0.71074	0.63983	0.19689	0.98495	0.98512	0.9835	0.53198	2.8674	1.8327	0.07344	0.85057	3.8527	0.009144	0.009144	0.009144
60	0.91582	2.371	0.1414	0.56933	1.3631	0.7652	0.76529	0.70059	0.22175	0.98831	0.98843	0.98413	0.55763	2.7849	1.8465	0.07051	0.83153	3.4577	0.0091147	0.0091147	0.0091147
61	0.91868	2.3297	0.14237	0.56346	1.3652	0.78712	0.78839	0.73323	0.22191	0.9817	0.98182	0.97479	0.58058	2.6738	1.6969	0.05785	0.82744	3.4239	0.0090849	0.0090849	0.0090849
62	0.915	2.2795	0.14083	0.56409	1.365	0.77181	0.7719	0.67205	0.19708	0.98997	0.99008	0.98924	0.56023	2.7676	1.7015	0.06903	0.80184	3.5462	0.0090546	0.0090546	0.0090546
63	0.91025	2.3115	0.14004	0.56883	1.3557	0.82469	0.82479	0.75829	0.22916	0.97344	0.97355	0.96371	0.55806	2.5869	1.6677	0.05762	0.82014	3.3086	0.0090239	0.0090239	0.0090239
64	0.91079	2.2652	0.13628	0.56541	1.3562	0.74041	0.7405	0.5973	0.14924	0.98006	0.98017	0.96861	0.52351	2.8365	1.7003	0.06284	0.86616	3.657	0.0089928	0.0089928	0.0089928
65	0.91782	2.3072	0.13913	0.56156	1.3581	0.81809	0.81818	0.786	0.24351	0.98171	0.98182	0.97916	0.55377	2.615	1.7788	0.06631	0.8187	3.325	0.0089613	0.0089613	0.0089613
66	0.91	2.2597	0.1352	0.56079	1.349	0.81146	0.81157	0.7492	0.23164	0.98499	0.98512	0.98107	0.57871	2.7014	1.6797	0.06399	0.73719	3.5325	0.0089293	0.0089293	0.0089293
67	0.91317	2.2196	0.13755	0.55628	1.3588	0.79164	0.79174	0.74286	0.22648	0.97839	0.97851	0.96985	0.56139	2.7999	1.7009	0.06203	0.82018	3.8239	0.0088969	0.0088969	0.0088969
68	0.89593	2.195	0.13318	0.54954	1.3446	0.79824	0.79835	0.75497	0.25603	0.9883	0.98843	0.98517	0.57626	2.6982	1.7158	0.07556	0.82657	3.6082	0.008864	0.008864	0.008864
69	0.91334	2.2561	0.1346	0.55937	1.3577	0.78999	0.79008	0.71687	0.2257	0.9817	0.98182	0.97756	0.53754	2.6945	1.7579	0.07943	0.76376	3.6908	0.0088308	0.0088308	0.0088308
70	0.89973	2.1949	0.13356	0.5537	1.3479	0.83937	0.83967	0.78184	0.22903	0.93521	0.93554	0.9006	0.46348	2.6077	2.532	0.84264	0.78576	3.4588	0.0087971	0.0087971	0.0087971
71	0.89675	2.1983	0.13254	0.55388	1.348	0.81147	0.81157	0.71146	0.19577	0.9817	0.98182	0.96965	0.52494	2.5699	1.6395	0.0477	0.77538	3.2414	0.008763	0.008763	0.008763
72	0.89445	2.1913	0.13286	0.55252	1.3437	0.82305	0.82314	0.7017	0.17622	0.98997	0.99008	0.98806	0.5542	2.5957	1.6343	0.06303	0.82366	3.3466	0.0087286	0.0087286	0.0087286
73	0.89671	2.1775	0.13251	0.54852	1.3442	0.79866	0.79989	0.73282	0.21454	0.99162	0.99174	0.98773	0.57488	2.5653	1.5901	0.04397	0.7844	3.2369	0.0086937	0.0086937	0.0086937
74	0.88669	2.1431	0.13001	0.54404	1.3395	0.74537	0.74545	0.60156	0.15144	0.98832	0.98843	0.98035	0.55611	2.7808	1.6513	0.07384	0.83709	3.7414	0.0086584	0.0086584	0.0086584
75	0.89534	2.1346	0.13114	0.54418	1.3414	0.75693	0.75702	0.62991	0.16266	0.98665	0.98678	0.9805	0.55838	2.8263	1.6401	0.07235	0.79863	3.6575	0.0086227	0.0086227	0.0086227
76	0.88303	2.1307	0.1316	0.53305	1.3333	0.73876	0.73884	0.62675	0.19061	0.98501	0.98512	0.9817	0.5988	2.85	1.6523	0.07351	0.78306	3.769	0.0085866	0.0085866	0.0085866
77	0.87709	2.1137	0.13162	0.53311	1.3287	0.72553	0.72562	0.59213	0.16964	0.98501	0.98512	0.97891	0.56976	2.8022	1.646	0.07157	0.80386	3.6825	0.0085502	0.0085502	0.0085502
78	0.88234	2.0998	0.12729	0.53634	1.3328	0.77555	0.77674	0.68431	0.20325	0.98336	0.98347	0.97295	0.5556	2.7236	1.5827	0.0605	0.76847	3.5618	0.0085133	0.0085133	0.0085133
79	0.88173	2.082	0.12886	0.54179	1.3372	0.83791	0.83802	0.78246	0.23119	0.98666	0.98678	0.9791	0.56893	2.5201	1.577	0.06091	0.76957	3.2387	0.0084761	0.0084761	0.0084761
80	0.88257	2.0881	0.12747	0.5362	1.337	0.80486	0.80496	0.7232	0.20533	0.98666	0.98678	0.97984	0.57914	2.6988	1.584	0.05366	0.76096	3.5833	0.0084385	0.0084385	0.0084385
81	0.88016	2.0598	0.12713	0.5371	1.3375	0.79496	0.79504	0.74119	0.21659	0.98832	0.98843	0.98489	0.59993	2.7771	1.5677	0.05502	0.81247	3.6516	0.0084005	0.0084005	0.0084005
82	0.86172	2.0414	0.12654	0.52842	1.3224	0.79165	0.79174	0.71902	0.23565	0.98997	0.99008	0.98806	0.61321	2.6648	1.5556	0.05224	0.79791	3.5675	0.0083622	0.0083622	0.0083622
83	0.86013	1.998	0.12627	0.5259	1.3183	0.78007	0.78017	0.67029	0.18388	0.98997	0.99008	0.98758	0.56883	2.6802	1.5752	0.05767	0.78372	3.5321	0.0083235	0.0083235	0.0083235
84	0.85858	2.0043	0.12431	0.52757	1.3182	0.79536	0.79661	0.75938	0.25874	0.98997	0.99008	0.9864	0.58416	2.6185	1.6079	0.06806	0.77701	3.416	0.0082844	0.0082844	0.0082844
85	0.86995	1.9804	0.12445	0.53055	1.3253	0.77555	0.77675	0.69993	0.20541	0.98501	0.98512	0.97474	0.58351	2.7164	1.5702	0.06174	0.7997	3.6285	0.008245	0.008245	0.008245
86	0.86406	2.0019	0.12204	0.53156	1.3215	0.80817	0.80826	0.72057	0.20968	0.98997	0.99008	0.98794	0.58773	2.6545	1.5261	0.05239	0.7721	3.5714	0.0082052	0.0082052	0.0082052
87	0.86053	1.9722	0.12357	0.52758	1.3191	0.7805	0.7817	0.71211	0.22158	0.98996	0.99008	0.98853	0.60617	2.6776	1.4538	0.04627	0.78238	3.587	0.0081651	0.0081651	0.0081651
88	0.84832	1.94	0.12115	0.51562	1.307	0.79165	0.79174	0.69214	0.20292	0.99162	0.99174	0.98976	0.60808	2.6661	1.426	0.04225	0.80749	3.6425	0.0081247	0.0081247	0.0081247
89	0.85561	1.9515	0.11988	0.52219	1.3117	0.79826	0.79835	0.6954	0.2054	0.99162	0.99174	0.99039	0.59287	2.5926	1.468	0.04228	0.78991	3.5141	0.0080839	0.0080839	0.0080839
90	0.86191	1.9235	0.12056	0.52324	1.3171	0.75033	0.75041	0.63603	0.19895	0.99162	0.99174	0.99014	0.61147	2.6749	1.4078	0.04295	0.81849	3.5833	0.0080428	0.0080428	0.0080428
91	0.85791	1.9476	0.12341	0.52524	1.3172	0.77181	0.7719	0.67435	0.19773	0.98832	0.98843	0.9824	0.5912	2.6724	1.4476	0.04259	0.81583	3.5982	0.0080013	0.0080013	0.0080013
92	0.86812	1.9382	0.1199	0.52537	1.3216	0.75198	0.75207	0.65162	0.20957	0.98996	0.99008	0.99046	0.62405	2.7589	1.4415	0.05284	0.79631	3.7482	0.0079595	0.0079595	0.0079595
93	0.85809	1.9294	0.12017	0.52079	1.317	0.79	0.79008	0.72223	0.24042	0.99163	0.99174	0.98959	0.62663	2.6908	1.4771	0.04891	0.76931	3.619	0.0079174	0.0079174	0.0079174
94	0.84691	1.9151	0.12069	0.51597	1.3071	0.78669	0.78678	0.69635	0.2246	0.99163	0.99174	0.98936	0.62759	2.6791	1.4671	0.05361	0.78474	3.669	0.007875	0.007875	0.007875
95	0.8437	1.8924	0.12052	0.51001	1.3041	0.78835	0.78843	0.69112	0.21785	0.99328	0.99339	0.99	0.61178	2.6718	1.4733	0.04963	0.81226	3.7317	0.0078323	0.0078323	0.0078323
96	0.84282	1.8686	0.11843	0.51081	1.3043	0.80157	0.80165	0.73419	0.24295	0.99163	0.99174	0.98976	0.62726	2.6494	1.4915	0.04847	0.78939	3.7431	0.0077893	0.0077893	0.0077893
97	0.84051	1.8517	0.11633	0.51266	1.3053	0.79661	0.79669	0.73206	0.24755	0.99163	0.99174	0.98846	0.63579	2.6041	1.4268	0.04687	0.76798	3.6554	0.007746	0.007746	0.007746
98	0.85144	1.8414	0.11899	0.51386	1.3105	0.79	0.79008	0.70522	0.23734	0.99163	0.99174	0.98834	0.63518	2.5979	1.384	0.04349	0.77369	3.6433	0.0077023	0.0077023	0.0077023
99	0.84403	1.8312	0.11738	0.50855	1.3028	0.78173	0.78182	0.71449	0.23685	0.99328	0.99339	0.98956	0.64983	2.6338	1.3476	0.03615	0.79592	3.6455	0.0076584	0.0076584	0.0076584
100	0.84854	1.8521	0.11793	0.51213	1.3088	0.79165	0.79174	0.72004	0.2318	0.99328	0.99339	0.99031	0.62985	2.5821	1.375	0.03566	0.78494	3.6066	0.0076142	0.0076142	0.0076142
101	0.83419	1.8117	0.1146	0.50378	1.2964	0.78008	0.78017	0.71128	0.24632	0.99328	0.99339	0.98998	0.63553	2.5733	1.3919	0.03486	0.7919	3.6264	0.0075698	0.0075698	0.0075698
102	0.82893	1.7849	0.11582	0.50752	1.2931	0.79495	0.79504	0.68954	0.214	0.99328	0.99339	0.99072	0.62522	2.5954	1.3927	0.04302	0.75155	3.6968	0.007525	0.007525	0.007525
103	0.83007	1.7629	0.11355	0.50572	1.2931	0.7933	0.79339	0.66909	0.18961	0.99162	0.99174	0.98882	0.5936	2.5691	1.4374	0.04201	0.76379	3.6013	0.00748	0.00748	0.00748
104	0.83836	1.8169	0.11478	0.50855	1.3023	0.79821	0.79835	0.69154	0.21573	0.98661	0.98678	0.9801	0.56385	2.5902	2.0397	0.05633	0.75193	3.6711	0.0074347	0.0074347	0.0074347
105	0.83555	1.7978	0.1162	0.51233	1.2988	0.78992	0.79008	0.69562	0.21325	0.98988	0.99008	0.98742	0.57312	2.6017	1.7232	0.06106	0.75652	3.6646	0.0073891	0.0073891	0.0073891
106	0.83197	1.7617	0.11408	0.50917	1.2971	0.77347	0.77355	0.65472	0.20034	0.99493	0.99504	0.99059	0.61334	2.5943	1.4699	0.0618	0.77385	3.7282	0.0073433	0.0073433	0.0073433
107	0.82861	1.7713	0.11411	0.50274	1.2936	0.79165	0.79174	0.69874	0.21006	0.98832	0.98843	0.97915	0.62021	2.6472	1.4375	0.05912	0.76947	3.7664	0.0072973	0.0072973	0.0072973
108	0.82544	1.7408	0.11152	0.4987	1.2943	0.78669	0.78678	0.68999	0.22119	0.98667	0.98678	0.98117	0.63312	2.6222	1.4063	0.05445	0.76652	3.6781	0.0072509	0.0072509	0.0072509
109	0.82897	1.7366	0.11165	0.50233	1.2953	0.79165	0.79174	0.71725	0.23425	0.99328	0.99339	0.98998	0.65036	2.6125	1.3679	0.04737	0.75102	3.6384	0.0072044	0.0072044	0.0072044
110	0.82013	1.7214	0.1111	0.49322	1.2841	0.79826	0.79835	0.75531	0.2584	0.99493	0.99504	0.99138	0.65142	2.576	1.3864	0.04988	0.73246	3.5972	0.0071576	0.0071576	0.0071576
111	0.81712	1.7094	0.11228	0.49403	1.2914	0.80157	0.80165	0.74371	0.24763	0.98998	0.99008	0.98788	0.6534	2.5806	1.3558	0.05002	0.73838	3.6298	0.0071106	0.0071106	0.0071106
112	0.81853	1.7207	0.11285	0.49355	1.2851	0.7933	0.79339	0.74261	0.25158	0.99163	0.99174	0.99006	0.66653	2.6148	1.3377	0.04792	0.73481	3.7047	0.0070633	0.0070633	0.0070633
113	0.81184	1.6861	0.11166	0.4906	1.2817	0.78835	0.78843	0.71394	0.25186	0.98998	0.99008	0.98956	0.66597	2.6239	1.326	0.04475	0.73159	3.7067	0.0070159	0.0070159	0.0070159
114	0.8155	1.6736	0.10926	0.49289	1.2866	0.78669	0.78678	0.71776	0.24436	0.99328	0.99339	0.99131	0.67559	2.6309	1.3312	0.04712	0.73098	3.7496	0.0069682	0.0069682	0.0069682
115	0.82093	1.6678	0.11003	0.49685	1.2864	0.79826	0.79835	0.72771	0.24625	0.99328	0.99339	0.9907	0.66395	2.6145	1.3481	0.04843	0.7371	3.7475	0.0069203	0.0069203	0.0069203
116	0.80814	1.6564	0.10899	0.48795	1.2818	0.79991	0.8	0.71976	0.24209	0.99328	0.99339	0.99132	0.66893	2.5885	1.3313	0.0483	0.73952	3.7139	0.0068722	0.0068722	0.0068722
117	0.81228	1.6502	0.10759	0.48897	1.2833	0.79991	0.8	0.7197	0.25117	0.99328	0.99339	0.98964	0.67338	2.5845	1.303	0.04634	0.73678	3.7053	0.0068239	0.0068239	0.0068239
118	0.80543	1.6299	0.10766	0.48398	1.2776	0.79991	0.8	0.72434	0.24816	0.99328	0.99339	0.9903	0.67936	2.5685	1.2866	0.04377	0.75253	3.6483	0.0067754	0.0067754	0.0067754
119	0.80647	1.6075	0.10488	0.49039	1.2799	0.80322	0.80331	0.73564	0.24273	0.99328	0.99339	0.98958	0.68199	2.5838	1.2746	0.0417	0.76987	3.6349	0.0067268	0.0067268	0.0067268
120	0.8081	1.6559	0.10818	0.48851	1.2802	0.79826	0.79835	0.72388	0.24201	0.99328	0.99339	0.98918	0.67447	2.6123	1.2747	0.04048	0.77246	3.6777	0.0066779	0.0066779	0.0066779
121	0.80976	1.5835	0.10471	0.48761	1.2771	0.80157	0.80165	0.72357	0.24258	0.99328	0.99339	0.98865	0.66893	2.6032	1.3239	0.04962	0.75191	3.626	0.0066288	0.0066288	0.0066288
122	0.80539	1.5891	0.10663	0.48151	1.2754	0.79496	0.79504	0.7402	0.25348	0.99659	0.99669	0.99323	0.6808	2.6088	1.3291	0.05278	0.74635	3.6721	0.0065796	0.0065796	0.0065796
123	0.80267	1.5742	0.10382	0.48819	1.2753	0.79661	0.79669	0.75415	0.25792	0.99659	0.99669	0.99288	0.6841	2.5884	1.3241	0.05167	0.74258	3.6894	0.0065303	0.0065303	0.0065303
124	0.79379	1.5637	0.10495	0.47869	1.2678	0.79165	0.79174	0.72944	0.24709	0.99659	0.99669	0.99268	0.67696	2.602	1.2938	0.04512	0.74343	3.7538	0.0064807	0.0064807	0.0064807
125	0.80366	1.561	0.10369	0.48436	1.2747	0.79165	0.79174	0.71848	0.24722	0.99659	0.99669	0.99224	0.67262	2.5766	1.2783	0.04107	0.74777	3.6613	0.006431	0.006431	0.006431
126	0.78906	1.522	0.1039	0.4765	1.2662	0.78835	0.78843	0.73362	0.25259	0.99659	0.99669	0.99223	0.67587	2.5937	1.2557	0.03868	0.7555	3.703	0.0063812	0.0063812	0.0063812
127	0.7905	1.5528	0.10483	0.47891	1.2666	0.79165	0.79174	0.7346	0.25437	0.99328	0.99339	0.98872	0.68232	2.5827	1.2457	0.03829	0.76253	3.6889	0.0063312	0.0063312	0.0063312
128	0.79101	1.5124	0.10186	0.47587	1.2675	0.78834	0.78843	0.7311	0.25159	0.99494	0.99504	0.99079	0.68182	2.5983	1.2597	0.04186	0.74842	3.7078	0.006281	0.006281	0.006281
129	0.79145	1.5259	0.10416	0.47839	1.2672	0.79165	0.79174	0.71494	0.24358	0.99493	0.99504	0.99148	0.68621	2.5754	1.2702	0.04379	0.73748	3.681	0.0062307	0.0062307	0.0062307
130	0.79023	1.505	0.10232	0.47886	1.2672	0.79165	0.79174	0.72135	0.24417	0.99493	0.99504	0.99039	0.69302	2.5779	1.2502	0.04353	0.73325	3.7115	0.0061803	0.0061803	0.0061803
131	0.79256	1.4971	0.10307	0.47895	1.2703	0.7933	0.79339	0.73303	0.25119	0.99494	0.99504	0.99083	0.69322	2.5605	1.2207	0.03753	0.74694	3.7015	0.0061298	0.0061298	0.0061298
132	0.78713	1.5032	0.10162	0.47693	1.2632	0.79165	0.79174	0.73243	0.25473	0.99494	0.99504	0.99138	0.70613	2.5627	1.2125	0.03913	0.74876	3.7184	0.0060792	0.0060792	0.0060792
133	0.78502	1.4807	0.10055	0.47482	1.2618	0.79496	0.79504	0.7368	0.25695	0.99494	0.99504	0.99143	0.70523	2.5591	1.2081	0.04006	0.74383	3.7136	0.0060284	0.0060284	0.0060284
134	0.77617	1.4687	0.10124	0.4735	1.2546	0.80157	0.80165	0.74647	0.26312	0.99494	0.99504	0.99143	0.70883	2.5488	1.2002	0.03802	0.74368	3.6916	0.0059775	0.0059775	0.0059775
135	0.77409	1.437	0.09932	0.46481	1.2518	0.79165	0.79174	0.7334	0.25863	0.99494	0.99504	0.99138	0.7091	2.5501	1.1866	0.03587	0.74986	3.6678	0.0059266	0.0059266	0.0059266
136	0.78022	1.4677	0.10136	0.47433	1.2591	0.79826	0.79835	0.74317	0.26028	0.99494	0.99504	0.99118	0.70857	2.5507	1.1912	0.03623	0.74732	3.6682	0.0058755	0.0058755	0.0058755
137	0.7682	1.436	0.09956	0.45903	1.2487	0.79496	0.79504	0.74509	0.26467	0.99494	0.99504	0.99123	0.70868	2.5696	1.1999	0.03816	0.74276	3.6991	0.0058244	0.0058244	0.0058244
138	0.77045	1.4134	0.09808	0.46444	1.2527	0.79165	0.79174	0.74567	0.26655	0.99494	0.99504	0.99123	0.71142	2.5682	1.1832	0.03846	0.75098	3.6796	0.0057731	0.0057731	0.0057731
139	0.7704	1.418	0.09913	0.45928	1.2474	0.79496	0.79504	0.74447	0.26878	0.99659	0.99669	0.99258	0.713	2.5607	1.1812	0.03932	0.74953	3.6577	0.0057218	0.0057218	0.0057218
140	0.77512	1.4178	0.09921	0.46436	1.2554	0.79826	0.79835	0.74584	0.26578	0.99659	0.99669	0.99267	0.71673	2.5567	1.1652	0.03698	0.75238	3.665	0.0056704	0.0056704	0.0056704
141	0.77322	1.407	0.09842	0.46092	1.2512	0.79	0.79008	0.73842	0.2634	0.99659	0.99669	0.99249	0.72549	2.5636	1.1624	0.03962	0.75014	3.6943	0.0056189	0.0056189	0.0056189
142	0.77179	1.4276	0.10006	0.46835	1.2501	0.78504	0.78512	0.7351	0.26195	0.99659	0.99669	0.99229	0.7245	2.5655	1.1614	0.04037	0.74628	3.7183	0.0055674	0.0055674	0.0055674
143	0.76823	1.3926	0.09724	0.46269	1.245	0.79165	0.79174	0.73645	0.26272	0.99659	0.99669	0.99249	0.7229	2.5575	1.1513	0.03963	0.74342	3.727	0.0055158	0.0055158	0.0055158
144	0.76362	1.3816	0.09705	0.45567	1.2461	0.7933	0.79339	0.73783	0.26171	0.99659	0.99669	0.99252	0.72359	2.5687	1.1451	0.03884	0.75073	3.7425	0.0054642	0.0054642	0.0054642
145	0.76603	1.3665	0.09659	0.4638	1.2508	0.7867	0.78678	0.7318	0.25915	0.99659	0.99669	0.99267	0.72236	2.5727	1.1388	0.03746	0.75431	3.7613	0.0054125	0.0054125	0.0054125
146	0.7606	1.3527	0.09682	0.45817	1.2423	0.78835	0.78843	0.735	0.25736	0.99659	0.99669	0.99265	0.72301	2.5764	1.1346	0.0365	0.75884	3.7725	0.0053608	0.0053608	0.0053608
147	0.7567	1.358	0.0972	0.45466	1.24	0.78504	0.78512	0.72795	0.25515	0.99659	0.99669	0.99262	0.72638	2.5827	1.1274	0.03613	0.76472	3.8049	0.0053091	0.0053091	0.0053091
148	0.75052	1.3264	0.09604	0.45657	1.2381	0.78504	0.78512	0.72975	0.25553	0.99659	0.99669	0.9926	0.73036	2.5836	1.1199	0.03447	0.76988	3.8324	0.0052573	0.0052573	0.0052573
149	0.75326	1.3196	0.09404	0.45649	1.2384	0.78008	0.78017	0.72598	0.25342	0.99659	0.99669	0.99255	0.73165	2.5874	1.1071	0.0329	0.78334	3.8414	0.0052055	0.0052055	0.0052055
150	0.74982	1.3298	0.09496	0.45315	1.2357	0.78174	0.78182	0.72861	0.25361	0.99659	0.99669	0.99232	0.72875	2.5813	1.0993	0.03231	0.78431	3.8255	0.0051537	0.0051537	0.0051537
151	0.75052	1.3048	0.09557	0.45228	1.2353	0.78669	0.78678	0.73495	0.25672	0.99659	0.99669	0.99261	0.73286	2.5765	1.096	0.03253	0.77562	3.8167	0.0051018	0.0051018	0.0051018
152	0.75039	1.3199	0.09521	0.45379	1.2367	0.78669	0.78678	0.73597	0.25602	0.99824	0.99835	0.99437	0.73625	2.5822	1.0927	0.03411	0.76585	3.8354	0.00505	0.00505	0.00505
153	0.74638	1.3064	0.09474	0.44723	1.2318	0.78835	0.78843	0.73483	0.25564	0.99824	0.99835	0.99409	0.74052	2.586	1.0897	0.03521	0.76193	3.857	0.0049982	0.0049982	0.0049982
154	0.74333	1.2871	0.09421	0.44689	1.2289	0.79165	0.79174	0.7439	0.25851	0.99824	0.99835	0.99394	0.7407	2.581	1.0937	0.03642	0.75811	3.8508	0.0049463	0.0049463	0.0049463
155	0.74893	1.2653	0.09281	0.44731	1.2314	0.79331	0.79339	0.74247	0.25936	0.99824	0.99835	0.99389	0.73754	2.5811	1.0905	0.0366	0.75616	3.8467	0.0048945	0.0048945	0.0048945
156	0.74522	1.2671	0.09371	0.44461	1.2329	0.79826	0.79835	0.74969	0.26062	0.99824	0.99835	0.99442	0.73986	2.5721	1.097	0.03736	0.74998	3.8339	0.0048427	0.0048427	0.0048427
157	0.74384	1.2494	0.09075	0.44469	1.2316	0.79826	0.79835	0.74855	0.25991	0.99824	0.99835	0.99447	0.74403	2.5672	1.0916	0.03718	0.74809	3.8348	0.0047909	0.0047909	0.0047909
158	0.73461	1.2497	0.09005	0.44534	1.2258	0.79826	0.79835	0.74412	0.25758	0.99825	0.99835	0.99445	0.74689	2.5744	1.0887	0.03796	0.75146	3.8618	0.0047392	0.0047392	0.0047392
159	0.73533	1.2198	0.09009	0.44034	1.2266	0.80157	0.80165	0.74782	0.26242	0.99824	0.99835	0.99445	0.75331	2.5734	1.0718	0.03594	0.75724	3.8574	0.0046875	0.0046875	0.0046875
160	0.73664	1.262	0.09145	0.43988	1.2289	0.79661	0.79669	0.73976	0.25949	0.99824	0.99835	0.99411	0.75033	2.5713	1.0658	0.0352	0.76772	3.8453	0.0046358	0.0046358	0.0046358
161	0.73967	1.2473	0.09109	0.45	1.23	0.79827	0.79835	0.74048	0.25907	0.99825	0.99835	0.99442	0.75172	2.5756	1.0672	0.03601	0.76335	3.8631	0.0045842	0.0045842	0.0045842
162	0.73926	1.2133	0.09132	0.4449	1.2309	0.79992	0.8	0.74158	0.26029	0.99825	0.99835	0.99436	0.75083	2.573	1.0667	0.03653	0.7618	3.8698	0.0045326	0.0045326	0.0045326
163	0.72524	1.2133	0.09043	0.43115	1.2165	0.79992	0.8	0.74143	0.26147	0.99825	0.99835	0.99426	0.75415	2.5739	1.0654	0.03661	0.76263	3.8857	0.0044811	0.0044811	0.0044811
164	0.73306	1.2201	0.08941	0.43933	1.2272	0.79661	0.79669	0.7294	0.26048	0.99824	0.99835	0.99427	0.75451	2.5729	1.0684	0.0374	0.75859	3.8803	0.0044296	0.0044296	0.0044296
165	0.73851	1.2111	0.09043	0.443	1.2271	0.79661	0.79669	0.72983	0.25924	0.99824	0.99835	0.99427	0.75374	2.5719	1.0736	0.03901	0.75591	3.8845	0.0043782	0.0043782	0.0043782
166	0.7346	1.2012	0.08756	0.43966	1.2248	0.79496	0.79504	0.727	0.25943	0.99824	0.99835	0.99431	0.75501	2.5729	1.0722	0.03966	0.75481	3.8924	0.0043269	0.0043269	0.0043269
167	0.72941	1.1767	0.08817	0.43557	1.2222	0.79331	0.79339	0.72644	0.25999	0.99824	0.99835	0.99432	0.75497	2.5727	1.0683	0.04005	0.75407	3.889	0.0042756	0.0042756	0.0042756
168	0.73068	1.2031	0.09059	0.43685	1.2225	0.79661	0.79669	0.73224	0.26153	0.99824	0.99835	0.99452	0.7589	2.5734	1.0679	0.0411	0.75088	3.8843	0.0042245	0.0042245	0.0042245
169	0.7297	1.178	0.08696	0.43962	1.2224	0.79827	0.79835	0.73541	0.26075	0.99825	0.99835	0.99452	0.76074	2.573	1.0666	0.04142	0.7495	3.8876	0.0041734	0.0041734	0.0041734

170	0.72002	1.1528	0.08808	0.43241	1.2136	0.79992	0.8	0.73055	0.26054	0.99824	0.99835	0.99452	0.76254	2.5638	1.0597	0.04041	0.75151	3.8742	0.0041225	0.0041225	0.0041225
171	0.72385	1.1635	0.08892	0.43639	1.2165	0.79827	0.79835	0.72817	0.26004	0.99824	0.99835	0.99452	0.76311	2.5592	1.0566	0.04035	0.75201	3.875	0.0040716	0.0040716	0.0040716
172	0.72518	1.1584	0.0864	0.43767	1.2197	0.79992	0.8	0.72988	0.26046	0.99824	0.99835	0.99454	0.76453	2.5636	1.0569	0.04075	0.74718	3.8865	0.0040208	0.0040208	0.0040208
173	0.71502	1.1492	0.08734	0.4278	1.2116	0.80157	0.80165	0.72947	0.25974	0.99824	0.99835	0.99437	0.76533	2.5596	1.0522	0.03992	0.74782	3.8801	0.0039702	0.0039702	0.0039702
174	0.71395	1.1305	0.0865	0.42335	1.2108	0.80322	0.80331	0.72928	0.25787	0.9999	1	0.995	0.76599	2.5626	1.0525	0.04036	0.74629	3.894	0.0039197	0.0039197	0.0039197
175	0.71685	1.1429	0.08525	0.42959	1.2141	0.80322	0.80331	0.72984	0.2596	0.9999	1	0.995	0.76631	2.5605	1.0428	0.03903	0.7518	3.8966	0.0038693	0.0038693	0.0038693
176	0.71326	1.1265	0.08492	0.42574	1.2101	0.80157	0.80165	0.73273	0.26068	0.9999	1	0.995	0.76633	2.562	1.037	0.03822	0.75375	3.9033	0.003819	0.003819	0.003819
177	0.7164	1.1108	0.08555	0.42854	1.2112	0.79992	0.8	0.73413	0.26191	0.9999	1	0.995	0.76911	2.5621	1.0387	0.03903	0.75385	3.9052	0.0037688	0.0037688	0.0037688
178	0.70903	1.0843	0.08463	0.42323	1.2055	0.79992	0.8	0.73945	0.26462	0.9999	1	0.995	0.7703	2.562	1.0428	0.03963	0.75203	3.9078	0.0037188	0.0037188	0.0037188
179	0.71258	1.1152	0.08616	0.43094	1.2126	0.79992	0.8	0.73662	0.26387	0.9999	1	0.995	0.76886	2.5597	1.0358	0.03922	0.75294	3.9058	0.003669	0.003669	0.003669
180	0.70259	1.0937	0.08628	0.41835	1.2033	0.79992	0.8	0.73561	0.26227	0.9999	1	0.995	0.76656	2.5581	1.0318	0.03881	0.75396	3.903	0.0036193	0.0036193	0.0036193
181	0.70552	1.0964	0.08378	0.42159	1.2064	0.80157	0.80165	0.73689	0.26312	0.9999	1	0.995	0.76684	2.5549	1.0293	0.03873	0.75344	3.8993	0.0035697	0.0035697	0.0035697
182	0.70048	1.0867	0.08427	0.41847	1.2033	0.80157	0.80165	0.7386	0.26598	0.9999	1	0.995	0.76677	2.5537	1.028	0.03847	0.7551	3.9036	0.0035204	0.0035204	0.0035204
183	0.69456	1.0603	0.08375	0.41741	1.2001	0.80322	0.80331	0.74176	0.26635	0.9999	1	0.995	0.76735	2.5543	1.0308	0.03932	0.75448	3.908	0.0034712	0.0034712	0.0034712
184	0.69321	1.046	0.0823	0.41704	1.1977	0.80488	0.80496	0.74527	0.26533	0.9999	1	0.995	0.7676	2.552	1.0273	0.03853	0.75621	3.906	0.0034221	0.0034221	0.0034221
185	0.69539	1.0359	0.08095	0.41435	1.1996	0.80322	0.80331	0.74258	0.26402	0.9999	1	0.995	0.76553	2.55	1.0213	0.03807	0.75646	3.9051	0.0033732	0.0033732	0.0033732
186	0.69816	1.0347	0.07991	0.42099	1.2033	0.80322	0.80331	0.74009	0.26476	0.9999	1	0.995	0.76594	2.5507	1.0178	0.0378	0.75504	3.9057	0.0033246	0.0033246	0.0033246
187	0.69878	1.027	0.07967	0.41892	1.2005	0.80157	0.80165	0.73478	0.26622	0.9999	1	0.995	0.76696	2.5526	1.0108	0.03677	0.75335	3.9072	0.0032761	0.0032761	0.0032761
188	0.69157	1.0139	0.07997	0.41086	1.1944	0.80157	0.80165	0.73596	0.26779	0.9999	1	0.995	0.76832	2.5518	1.0095	0.03657	0.7524	3.9068	0.0032278	0.0032278	0.0032278
189	0.69651	1.0284	0.08106	0.41621	1.1992	0.80157	0.80165	0.73417	0.26403	0.9999	1	0.995	0.76862	2.5503	1.0078	0.03651	0.75253	3.9086	0.0031797	0.0031797	0.0031797
190	0.69336	1.0158	0.0812	0.41722	1.1975	0.80157	0.80165	0.73482	0.26335	0.9999	1	0.995	0.77021	2.5504	1.0071	0.03671	0.75395	3.909	0.0031318	0.0031318	0.0031318
191	0.69055	1.0091	0.07877	0.41584	1.1946	0.80157	0.80165	0.73505	0.26306	0.9999	1	0.995	0.77043	2.5501	1.0051	0.0365	0.75466	3.9108	0.0030841	0.0030841	0.0030841
192	0.68882	1.0248	0.08001	0.41375	1.196	0.80322	0.80331	0.73708	0.26429	0.9999	1	0.995	0.77092	2.549	1.0025	0.03649	0.75491	3.912	0.0030367	0.0030367	0.0030367
193	0.67851	0.98006	0.07933	0.40781	1.1877	0.80157	0.80165	0.73695	0.26154	0.9999	1	0.995	0.77092	2.5496	1.001	0.03681	0.75528	3.9151	0.0029894	0.0029894	0.0029894
194	0.68156	0.98312	0.07754	0.40908	1.1909	0.80322	0.80331	0.73864	0.26309	0.9999	1	0.995	0.76989	2.5493	0.99966	0.03686	0.75412	3.916	0.0029424	0.0029424	0.0029424
195	0.68324	0.97661	0.08037	0.40652	1.1922	0.80157	0.80165	0.73801	0.26468	0.9999	1	0.995	0.76922	2.5464	0.99484	0.03617	0.75676	3.9084	0.0028956	0.0028956	0.0028956
196	0.67892	0.9694	0.07788	0.41197	1.1916	0.79992	0.8	0.74071	0.26541	0.9999	1	0.995	0.76992	2.547	0.9948	0.03631	0.75594	3.9088	0.0028491	0.0028491	0.0028491
197	0.68578	0.98066	0.07963	0.40742	1.1912	0.79992	0.8	0.74291	0.26625	0.9999	1	0.995	0.76871	2.5441	0.99387	0.0363	0.75877	3.906	0.0028027	0.0028027	0.0028027
198	0.67453	0.94885	0.07864	0.40375	1.1853	0.79992	0.8	0.74304	0.26517	0.9999	1	0.995	0.76951	2.5425	0.99171	0.03609	0.76152	3.9029	0.0027567	0.0027567	0.0027567
199	0.68139	0.95635	0.07669	0.40645	1.1901	0.79992	0.8	0.74311	0.26532	0.99825	0.99835	0.99393	0.76901	2.5425	0.99127	0.03628	0.76283	3.9042	0.0027109	0.0027109	0.0027109
200	0.67266	0.93822	0.07788	0.40274	1.1848	0.79992	0.8	0.74355	0.26496	0.99825	0.99835	0.99394	0.77099	2.5433	0.9891	0.03665	0.76148	3.9072	0.0026653	0.0026653	0.0026653
201	0.6745	0.94242	0.07504	0.40674	1.1842	0.79992	0.8	0.74462	0.26632	0.9999	1	0.995	0.77179	2.5442	0.98875	0.03621	0.76208	3.9103	0.00262	0.00262	0.00262
202	0.66964	0.93761	0.0763	0.39887	1.1804	0.79992	0.8	0.74286	0.26551	0.9999	1	0.995	0.7757	2.5444	0.98725	0.03554	0.76168	3.9133	0.002575	0.002575	0.002575
203	0.6694	0.93893	0.07505	0.40137	1.1811	0.79992	0.8	0.74299	0.26598	0.99824	0.99835	0.99429	0.77714	2.5449	0.98704	0.03578	0.76168	3.9153	0.0025302	0.0025302	0.0025302
204	0.66906	0.9302	0.07665	0.39977	1.1819	0.79992	0.8	0.74154	0.26546	0.99824	0.99835	0.99432	0.77688	2.545	0.98834	0.03627	0.76138	3.9194	0.0024858	0.0024858	0.0024858
205	0.66483	0.9121	0.07599	0.3987	1.1751	0.79827	0.79835	0.73788	0.2656	0.99824	0.99835	0.99432	0.77884	2.544	0.98708	0.0364	0.76324	3.921	0.0024416	0.0024416	0.0024416
206	0.66292	0.88698	0.07359	0.39771	1.1811	0.79827	0.79835	0.73777	0.26624	0.9999	1	0.995	0.7798	2.5431	0.98585	0.03626	0.76294	3.9191	0.0023977	0.0023977	0.0023977
207	0.66097	0.90016	0.0723	0.39439	1.1753	0.79827	0.79835	0.73621	0.26635	0.9999	1	0.995	0.77943	2.5413	0.98328	0.03622	0.76553	3.919	0.002354	0.002354	0.002354
208	0.65598	0.88028	0.07451	0.39379	1.1735	0.79992	0.8	0.73745	0.26606	0.9999	1	0.995	0.77997	2.5411	0.98376	0.0365	0.7663	3.9187	0.0023107	0.0023107	0.0023107
209	0.67185	0.89191	0.07398	0.40225	1.1833	0.79827	0.79835	0.73425	0.26607	0.9999	1	0.995	0.7814	2.5414	0.98201	0.03646	0.7657	3.9185	0.0022677	0.0022677	0.0022677
210	0.65391	0.88872	0.07338	0.39082	1.1721	0.79992	0.8	0.73624	0.26599	0.9999	1	0.995	0.78143	2.5418	0.98123	0.03616	0.76609	3.9198	0.002225	0.002225	0.002225
211	0.65477	0.89203	0.07208	0.39296	1.1705	0.79992	0.8	0.73471	0.26574	0.9999	1	0.995	0.78242	2.543	0.98062	0.03653	0.76461	3.9234	0.0021826	0.0021826	0.0021826
212	0.66024	0.88953	0.07332	0.39323	1.1738	0.79992	0.8	0.73262	0.26447	0.9999	1	0.995	0.78289	2.5438	0.97886	0.03641	0.76571	3.9262	0.0021405	0.0021405	0.0021405
213	0.6559	0.8742	0.07364	0.39297	1.1717	0.79992	0.8	0.73306	0.26505	0.9999	1	0.995	0.78375	2.5437	0.97885	0.0368	0.7658	3.929	0.0020987	0.0020987	0.0020987
214	0.65709	0.85742	0.07128	0.39307	1.1724	0.79992	0.8	0.73269	0.26483	0.9999	1	0.995	0.78264	2.5437	0.97736	0.03708	0.76453	3.931	0.0020572	0.0020572	0.0020572
215	0.65049	0.85131	0.07155	0.38859	1.1687	0.79992	0.8	0.73349	0.26345	0.9999	1	0.995	0.78254	2.5443	0.97688	0.03713	0.76337	3.9336	0.0020161	0.0020161	0.0020161
216	0.64646	0.84634	0.0719	0.38697	1.1694	0.79992	0.8	0.73306	0.26458	0.9999	1	0.995	0.78306	2.5441	0.97586	0.03744	0.76283	3.936	0.0019753	0.0019753	0.0019753
217	0.65015	0.84723	0.07355	0.38806	1.1681	0.79992	0.8	0.73299	0.26464	0.99825	0.99835	0.99386	0.78302	2.5433	0.97563	0.03775	0.76361	3.9367	0.0019349	0.0019349	0.0019349
218	0.64848	0.83674	0.071	0.38603	1.1649	0.79827	0.79835	0.72992	0.26458	0.9999	1	0.995	0.78236	2.5424	0.97588	0.03799	0.76513	3.9371	0.0018948	0.0018948	0.0018948
219	0.6471	0.84362	0.06924	0.38708	1.1636	0.79827	0.79835	0.72777	0.26454	0.9999	1	0.995	0.78296	2.5415	0.97575	0.03818	0.76653	3.939	0.001855	0.001855	0.001855
220	0.64595	0.82698	0.06961	0.38413	1.1656	0.79661	0.79669	0.72654	0.26454	0.9999	1	0.995	0.78354	2.5413	0.9771	0.03831	0.76699	3.9414	0.0018156	0.0018156	0.0018156
221	0.64801	0.82707	0.0693	0.38728	1.165	0.79992	0.8	0.73104	0.26502	0.9999	1	0.995	0.78407	2.5407	0.97616	0.03833	0.76674	3.9425	0.0017765	0.0017765	0.0017765
222	0.6417	0.81933	0.06873	0.38813	1.1621	0.79661	0.79669	0.72759	0.26461	0.9999	1	0.995	0.78545	2.5413	0.97631	0.03828	0.76641	3.9455	0.0017378	0.0017378	0.0017378
223	0.64275	0.81508	0.07044	0.38547	1.1616	0.79992	0.8	0.73141	0.26484	0.9999	1	0.995	0.78509	2.5412	0.97651	0.03846	0.76685	3.947	0.0016995	0.0016995	0.0016995
224	0.63728	0.80988	0.06729	0.38281	1.1583	0.79992	0.8	0.73162	0.26544	0.9999	1	0.995	0.78397	2.5413	0.97762	0.03901	0.76717	3.9493	0.0016615	0.0016615	0.0016615
225	0.64519	0.80885	0.06952	0.38419	1.1661	0.79827	0.79835	0.73088	0.26513	0.9999	1	0.995	0.78464	2.5397	0.9771	0.03885	0.7692	3.9496	0.0016239	0.0016239	0.0016239
226	0.63731	0.79988	0.06666	0.37961	1.1581	0.79992	0.8	0.7336	0.26526	0.9999	1	0.995	0.78479	2.5383	0.97612	0.03881	0.77019	3.951	0.0015867	0.0015867	0.0015867
227	0.63264	0.78771	0.06666	0.37641	1.154	0.79992	0.8	0.73357	0.26454	0.9999	1	0.995	0.78555	2.5382	0.97553	0.03902	0.77033	3.9535	0.0015498	0.0015498	0.0015498
228	0.62908	0.78124	0.06854	0.37445	1.1518	0.79827	0.79835	0.73164	0.26506	0.9999	1	0.995	0.78483	2.5366	0.9743	0.03895	0.77111	3.9538	0.0015134	0.0015134	0.0015134
229	0.63325	0.79025	0.06899	0.37909	1.1598	0.79827	0.79835	0.73137	0.26459	0.9999	1	0.995	0.78592	2.5361	0.97308	0.03877	0.77179	3.9552	0.0014773	0.0014773	0.0014773
230	0.63446	0.78555	0.06767	0.37771	1.1566	0.79661	0.79669	0.7297	0.26445	0.9999	1	0.995	0.7842	2.537	0.97319	0.03921	0.77069	3.9587	0.0014416	0.0014416	0.0014416
231	0.62954	0.77503	0.0676	0.37619	1.1547	0.79496	0.79504	0.72866	0.26465	0.9999	1	0.995	0.78636	2.5366	0.97487	0.03952	0.77116	3.9604	0.0014063	0.0014063	0.0014063
232	0.62365	0.77077	0.06675	0.37564	1.1526	0.79661	0.79669	0.73047	0.26487	0.9999	1	0.995	0.78589	2.5359	0.97407	0.03934	0.77104	3.9609	0.0013714	0.0013714	0.0013714
233	0.63079	0.76957	0.06585	0.37796	1.1553	0.79661	0.79669	0.73084	0.26527	0.9999	1	0.995	0.78617	2.5358	0.97348	0.03959	0.77024	3.9607	0.001337	0.001337	0.001337
234	0.62651	0.75934	0.06389	0.37582	1.153	0.79331	0.79339	0.72676	0.26445	0.9999	1	0.995	0.78635	2.5356	0.97441	0.03981	0.77054	3.9633	0.0013029	0.0013029	0.0013029
235	0.62045	0.75088	0.06551	0.37365	1.1463	0.79	0.79008	0.72181	0.26397	0.9999	1	0.995	0.7865	2.5355	0.9751	0.03991	0.77089	3.9653	0.0012692	0.0012692	0.0012692
236	0.61998	0.76987	0.06601	0.36857	1.1472	0.79	0.79008	0.7211	0.26358	0.9999	1	0.995	0.78651	2.5345	0.97386	0.0398	0.77207	3.9649	0.001236	0.001236	0.001236
237	0.61782	0.75183	0.06513	0.36767	1.1477	0.79166	0.79174	0.72262	0.26379	0.9999	1	0.995	0.78597	2.5341	0.97362	0.03976	0.77181	3.9656	0.0012031	0.0012031	0.0012031
238	0.61613	0.74842	0.06616	0.36929	1.1451	0.79	0.79008	0.7218	0.26358	0.9999	1	0.995	0.78617	2.5338	0.97252	0.03972	0.77187	3.9673	0.0011707	0.0011707	0.0011707
239	0.61405	0.73691	0.06469	0.36895	1.1445	0.79	0.79008	0.72306	0.26431	0.9999	1	0.995	0.78602	2.5328	0.97295	0.03963	0.77266	3.968	0.0011387	0.0011387	0.0011387
240	0.61611	0.73674	0.06409	0.36695	1.1497	0.79166	0.79174	0.72597	0.26466	0.9999	1	0.995	0.7866	2.5329	0.97361	0.03964	0.7722	3.9705	0.0011072	0.0011072	0.0011072
241	0.61652	0.74893	0.06369	0.36889	1.1457	0.79166	0.79174	0.72722	0.26575	0.9999	1	0.995	0.78416	2.533	0.97468	0.03954	0.77252	3.9726	0.0010761	0.0010761	0.0010761
242	0.61869	0.7086	0.0634	0.36924	1.1486	0.79166	0.79174	0.72941	0.26599	0.9999	1	0.995	0.78482	2.5326	0.97649	0.03967	0.77256	3.9744	0.0010454	0.0010454	0.0010454
243	0.61253	0.72861	0.06419	0.36749	1.1462	0.79166	0.79174	0.72804	0.26551	0.9999	1	0.995	0.78497	2.5325	0.97599	0.03963	0.77335	3.9769	0.0010151	0.0010151	0.0010151
244	0.6103	0.71403	0.06347	0.36714	1.1456	0.79166	0.79174	0.72725	0.26548	0.9999	1	0.995	0.78572	2.5327	0.97532	0.03945	0.77253	3.9793	0.00098531	0.00098531	0.00098531
245	0.60921	0.72398	0.0661	0.36444	1.1414	0.79166	0.79174	0.72624	0.26535	0.9999	1	0.995	0.78584	2.5318	0.97573	0.03952	0.77342	3.9797	0.00095595	0.00095595	0.00095595
246	0.60927	0.7048	0.0625	0.36222	1.1405	0.79166	0.79174	0.72506	0.26505	0.9999	1	0.995	0.78616	2.531	0.97539	0.03942	0.77399	3.9808	0.00092704	0.00092704	0.00092704
247	0.60793	0.70261	0.06282	0.36087	1.1422	0.79166	0.79174	0.72458	0.26478	0.9999	1	0.995	0.78591	2.5308	0.97625	0.03964	0.77381	3.9827	0.00089858	0.00089858	0.00089858
248	0.59678	0.69151	0.06233	0.35589	1.1346	0.79331	0.79339	0.72685	0.26476	0.9999	1	0.995	0.78492	2.5302	0.97634	0.03943	0.77607	3.9852	0.00087058	0.00087058	0.00087058
249	0.60702	0.70764	0.06223	0.36465	1.1435	0.79496	0.79504	0.72812	0.2651	0.9999	1	0.995	0.7856	2.5303	0.97596	0.03941	0.77622	3.988	0.00084303	0.00084303	0.00084303
250	0.5995	0.67718	0.06199	0.35728	1.1348	0.79496	0.79504	0.72685	0.26436	0.9999	1	0.995	0.7852	2.5319	0.98012	0.04034	0.77484	3.9939	0.00081595	0.00081595	0.00081595
251	0.60652	0.70048	0.06349	0.36201	1.1392	0.79661	0.79669	0.72971	0.26432	0.9999	1	0.995	0.78412	2.5317	0.97961	0.03992	0.77637	3.9961	0.00078933	0.00078933	0.00078933
252	0.60333	0.68805	0.06275	0.36158	1.1357	0.79661	0.79669	0.72962	0.26402	0.9999	1	0.995	0.78335	2.5339	0.98317	0.04039	0.77456	4.0035	0.00076317	0.00076317	0.00076317
253	0.60547	0.69953	0.06196	0.36582	1.1423	0.79827	0.79835	0.73102	0.26383	0.9999	1	0.995	0.78341	2.5341	0.9842	0.04064	0.77531	4.0069	0.00073749	0.00073749	0.00073749
254	0.59635	0.68683	0.06122	0.35655	1.1334	0.79827	0.79835	0.7293	0.2636	0.9999	1	0.995	0.78303	2.5338	0.98574	0.0407	0.77582	4.0091	0.00071228	0.00071228	0.00071228
255	0.59859	0.68835	0.06214	0.35755	1.1355	0.79661	0.79669	0.72805	0.26343	0.9999	1	0.995	0.78211	2.5331	0.98543	0.04054	0.7761	4.0099	0.00068755	0.00068755	0.00068755
256	0.59434	0.6753	0.06024	0.35523	1.1323	0.79661	0.79669	0.72793	0.26335	0.9999	1	0.995	0.78193	2.5322	0.98586	0.04049	0.77672	4.0109	0.00066329	0.00066329	0.00066329
257	0.59297	0.64901	0.05983	0.35374	1.129	0.79661	0.79669	0.72859	0.26364	0.9999	1	0.995	0.78187	2.5323	0.98622	0.04044	0.77629	4.0132	0.00063952	0.00063952	0.00063952
258	0.59505	0.66457	0.05959	0.35483	1.1341	0.79827	0.79835	0.73168	0.26378	0.9999	1	0.995	0.78024	2.533	0.98808	0.04061	0.77593	4.0186	0.00061623	0.00061623	0.00061623
259	0.59491	0.66174	0.05996	0.35444	1.1346	0.79661	0.79669	0.73136	0.26438	0.9999	1	0.995	0.77986	2.5328	0.98869	0.04059	0.7771	4.0213	0.00059342	0.00059342	0.00059342
260	0.59523	0.67748	0.06241	0.3532	1.1327	0.79661	0.79669	0.7318	0.26472	0.9999	1	0.995	0.77952	2.5325	0.98935	0.04053	0.77782	4.0243	0.00057111	0.00057111	0.00057111
261	0.59689	0.6531	0.05967	0.35366	1.1352	0.79827	0.79835	0.73475	0.26528	0.9999	1	0.995	0.77836	2.5323	0.98925	0.04052	0.77799	4.0261	0.00054928	0.00054928	0.00054928
262	0.59322	0.66788	0.05921	0.35578	1.1295	0.79992	0.8	0.73686	0.26489	0.9999	1	0.995	0.77862	2.5325	0.99156	0.04083	0.77729	4.029	0.00052795	0.00052795	0.00052795
263	0.59355	0.64058	0.05755	0.35156	1.1282	0.79827	0.79835	0.73572	0.2651	0.9999	1	0.995	0.778	2.5319	0.99293	0.04067	0.77781	4.0306	0.00050711	0.00050711	0.00050711
264	0.59131	0.64418	0.05888	0.35446	1.1309	0.79827	0.79835	0.73586	0.26487	0.9999	1	0.995	0.77622	2.5316	0.99314	0.04075	0.7784	4.0329	0.00048678	0.00048678	0.00048678
265	0.58796	0.64768	0.0596	0.35334	1.1322	0.79992	0.8	0.73853	0.26495	0.9999	1	0.995	0.77698	2.5314	0.99357	0.0408	0.7784	4.0346	0.00046694	0.00046694	0.00046694
266	0.58827	0.65697	0.05865	0.35081	1.1279	0.79827	0.79835	0.73736	0.2651	0.9999	1	0.995	0.77729	2.5306	0.99473	0.04067	0.77954	4.0369	0.00044761	0.00044761	0.00044761
267	0.58296	0.65353	0.05862	0.35027	1.1241	0.79992	0.8	0.73942	0.2651	0.9999	1	0.995	0.77703	2.5304	0.99798	0.04085	0.77909	4.039	0.00042878	0.00042878	0.00042878
268	0.58421	0.65582	0.05925	0.34807	1.1264	0.79992	0.8	0.73948	0.26499	0.9999	1	0.995	0.77617	2.5298	0.99852	0.04085	0.77932	4.0404	0.00041045	0.00041045	0.00041045
269	0.58374	0.64718	0.06046	0.34774	1.1241	0.79827	0.79835	0.73839	0.26501	0.9999	1	0.995	0.77546	2.5302	1.0002	0.04084	0.7786	4.0432	0.00039264	0.00039264	0.00039264
270	0.58495	0.63541	0.05884	0.34821	1.1252	0.79992	0.8	0.74062	0.26582	0.9999	1	0.995	0.77547	2.5298	1.0009	0.04078	0.77894	4.0449	0.00037534	0.00037534	0.00037534
271	0.58636	0.63493	0.0596	0.3499	1.1262	0.79992	0.8	0.74016	0.26525	0.9999	1	0.995	0.77451	2.5295	1.0016	0.04048	0.78017	4.0471	0.00035855	0.00035855	0.00035855
272	0.58902	0.65348	0.06005	0.34649	1.1279	0.79992	0.8	0.73982	0.26526	0.9999	1	0.995	0.77384	2.529	1.003	0.04051	0.78012	4.0484	0.00034227	0.00034227	0.00034227
273	0.58074	0.638	0.05871	0.3497	1.1278	0.79992	0.8	0.74138	0.26511	0.9999	1	0.995	0.77297	2.5301	1.0057	0.04072	0.77989	4.0529	0.00032651	0.00032651	0.00032651
274	0.58524	0.623	0.05882	0.34965	1.1272	0.79992	0.8	0.74156	0.26547	0.9999	1	0.995	0.77321	2.5304	1.0076	0.04074	0.78069	4.0559	0.00031127	0.00031127	0.00031127
275	0.57608	0.63467	0.05929	0.34718	1.1237	0.79992	0.8	0.74144	0.26541	0.9999	1	0.995	0.77266	2.5307	1.012	0.04135	0.78068	4.0596	0.00029655	0.00029655	0.00029655
276	0.57678	0.6157	0.0571	0.34516	1.1207	0.79992	0.8	0.74177	0.26558	0.9999	1	0.995	0.77213	2.5307	1.0135	0.04142	0.78063	4.0627	0.00028235	0.00028235	0.00028235
277	0.57675	0.62498	0.05751	0.3428	1.1178	0.79992	0.8	0.74153	0.26533	0.9999	1	0.995	0.77195	2.5315	1.0154	0.04154	0.78126	4.0673	0.00026867	0.00026867	0.00026867
278	0.57587	0.62312	0.05854	0.34512	1.1222	0.79992	0.8	0.74269	0.26567	0.9999	1	0.995	0.77232	2.5311	1.0181	0.04159	0.78203	4.0707	0.00025551	0.00025551	0.00025551
279	0.57555	0.60928	0.05657	0.34384	1.1206	0.79992	0.8	0.74256	0.2658	0.9999	1	0.995	0.77164	2.5312	1.0204	0.04123	0.78255	4.0743	0.00024289	0.00024289	0.00024289
280	0.57229	0.61216	0.05714	0.34192	1.1202	0.79992	0.8	0.74241	0.26579	0.9999	1	0.995	0.7713	2.5314	1.024	0.04184	0.78277	4.0778	0.00023078	0.00023078	0.00023078
281	0.579	0.62396	0.05679	0.34357	1.1215	0.79992	0.8	0.74253	0.26598	0.9999	1	0.995	0.77038	2.5309	1.0258	0.04182	0.78304	4.0795	0.00021921	0.00021921	0.00021921
282	0.57749	0.62422	0.05864	0.34765	1.1239	0.79992	0.8	0.74216	0.26614	0.9999	1	0.995	0.77049	2.5308	1.0279	0.04185	0.78297	4.0819	0.00020817	0.00020817	0.00020817
283	0.57494	0.61336	0.05701	0.34307	1.1215	0.79992	0.8	0.7415	0.2659	0.9999	1	0.995	0.76861	2.5307	1.0308	0.04177	0.78375	4.0846	0.00019766	0.00019766	0.00019766
284	0.57376	0.60866	0.0564	0.34221	1.119	0.79992	0.8	0.74217	0.2662	0.9999	1	0.995	0.76958	2.5304	1.0333	0.04169	0.7847	4.0868	0.00018768	0.00018768	0.00018768
285	0.56823	0.61233	0.0592	0.34045	1.1127	0.79992	0.8	0.74138	0.26584	0.9999	1	0.995	0.76743	2.5305	1.0358	0.04172	0.78476	4.089	0.00017823	0.00017823	0.00017823
286	0.57352	0.61854	0.05783	0.34011	1.1207	0.79992	0.8	0.7417	0.26616	0.9999	1	0.995	0.76644	2.5308	1.0384	0.04172	0.78552	4.0924	0.00016932	0.00016932	0.00016932
287	0.57378	0.60328	0.057	0.34291	1.1203	0.79992	0.8	0.74092	0.2658	0.9999	1	0.995	0.76625	2.531	1.0405	0.04171	0.78624	4.0963	0.00016094	0.00016094	0.00016094
288	0.56981	0.61129	0.0558	0.3418	1.1197	0.79992	0.8	0.74157	0.26619	0.9999	1	0.995	0.76547	2.5311	1.0437	0.04182	0.78608	4.0993	0.0001531	0.0001531	0.0001531
289	0.57568	0.61018	0.0573	0.34663	1.1203	0.79992	0.8	0.74134	0.26636	0.9999	1	0.995	0.76572	2.5313	1.0459	0.04183	0.78687	4.1021	0.0001458	0.0001458	0.0001458
290	0.57527	0.59926	0.05632	0.34454	1.121	0.79992	0.8	0.74139	0.26599	0.9999	1	0.995	0.76376	2.5314	1.0484	0.04183	0.78734	4.1054	0.00013903	0.00013903	0.00013903
291	0.33347	0.22742	0.0111	0.18761	0.96122	0.79992	0.8	0.74157	0.26613	0.9999	1	0.995	0.76502	2.5312	1.0503	0.042	0.78584	4.1105	0.0001328	0.0001328	0.0001328
292	0.32265	0.21598	0.01046	0.16332	0.95268	0.79992	0.8	0.7411	0.26596	0.9999	1	0.995	0.76392	2.5305	1.0516	0.042	0.78527	4.1139	0.00012712	0.00012712	0.00012712
293	0.31863	0.21088	0.00987	0.16065	0.94961	0.79992	0.8	0.74095	0.26631	0.9999	1	0.995	0.76391	2.5299	1.0536	0.0421	0.78573	4.1168	0.00012197	0.00012197	0.00012197
294	0.31089	0.20431	0.00987	0.15751	0.94826	0.79992	0.8	0.74079	0.26679	0.9999	1	0.995	0.76329	2.5293	1.0547	0.04207	0.7858	4.1207	0.00011736	0.00011736	0.00011736
295	0.30985	0.20565	0.0095	0.15678	0.94771	0.79992	0.8	0.74039	0.26653	0.9999	1	0.995	0.76261	2.5287	1.057	0.04198	0.78553	4.1241	0.00011329	0.00011329	0.00011329
296	0.30901	0.20492	0.01001	0.15532	0.94668	0.79992	0.8	0.74051	0.26706	0.9999	1	0.995	0.76103	2.5278	1.0579	0.04194	0.78592	4.1264	0.00010977	0.00010977	0.00010977
297	0.30823	0.20065	0.00911	0.15467	0.94378	0.79992	0.8	0.74025	0.26703	0.9999	1	0.995	0.75967	2.5273	1.0604	0.04186	0.78555	4.129	0.00010678	0.00010678	0.00010678
298	0.30265	0.19818	0.00952	0.1528	0.93965	0.79992	0.8	0.74021	0.26687	0.9999	1	0.995	0.75978	2.5268	1.0617	0.04185	0.78606	4.1328	0.00010434	0.00010434	0.00010434
299	0.30386	0.19804	0.00951	0.15257	0.94248	0.79992	0.8	0.74141	0.26676	0.9999	1	0.995	0.75923	2.5264	1.0622	0.0418	0.78638	4.1361	0.00010244	0.00010244	0.00010244
300	0.30152	0.19786	0.00963	0.15138	0.93808	0.79992	0.8	0.74031	0.26648	0.9999	1	0.995	0.75912	2.525	1.0635	0.04174	0.78715	4.1378	0.00010109	0.00010109	0.00010109
