# Supplementary material for: Real-time location of acupuncture points based on anatomical landmarks and pose estimation models
Source: Front Neurorobot. 2024 Nov 8;18:1484038. doi: 10.3389/fnbot.2024.1484038 (PMC11609928; doi:10.3389/fnbot.2024.1484038)
Supplement: SUPPLEMENTARY VIDEO S1 — Real-time facial acupoint detection using MediaPipe. [file Data_Sheet_1.ZIP › Supplementary/Supplementary_Material.docx]

Supplementary Material

# Participant Inclusion and Data Collection

This study involved 194 participants recruited from Pukyong National University (n=94) and Dongshin University (n=100) in South Korea. Participants were adults aged 18 years or older, with no visible skin conditions or injuries on the arms that could interfere with acupoint identification. All participants were willing and able to provide informed consent.

No personal identifiers or additional health information beyond age and sex were collected or stored with the images. All data were anonymized and stored securely in compliance with data protection regulations.

Participants' involvement was limited to a single session where arm images were captured. No follow-up or additional participation was required. This study did not involve any medical interventions or treatments. The images and annotations were used solely for the purpose of developing and training an acupoint detection model.

# Supplementary Data

Further data is available in a zip file containing:

- acupoints_data.xlsx (**Table S1)** - Excel spreadsheet with a detailed table of acupoint locations and measurement details used for pose estimation with MediaPipe.
- training_results.csv (**Table S2)** - Contains detailed metrics on loss, accuracy, recall and mAP, for the YOLOv8-pose model during training and validation.

args.yaml - YAML configuration files were used to specify parameters like the hyperparameters for training the YOLOv8-pose model.

# Supplementary Figures and Tables

## Supplementary Figures


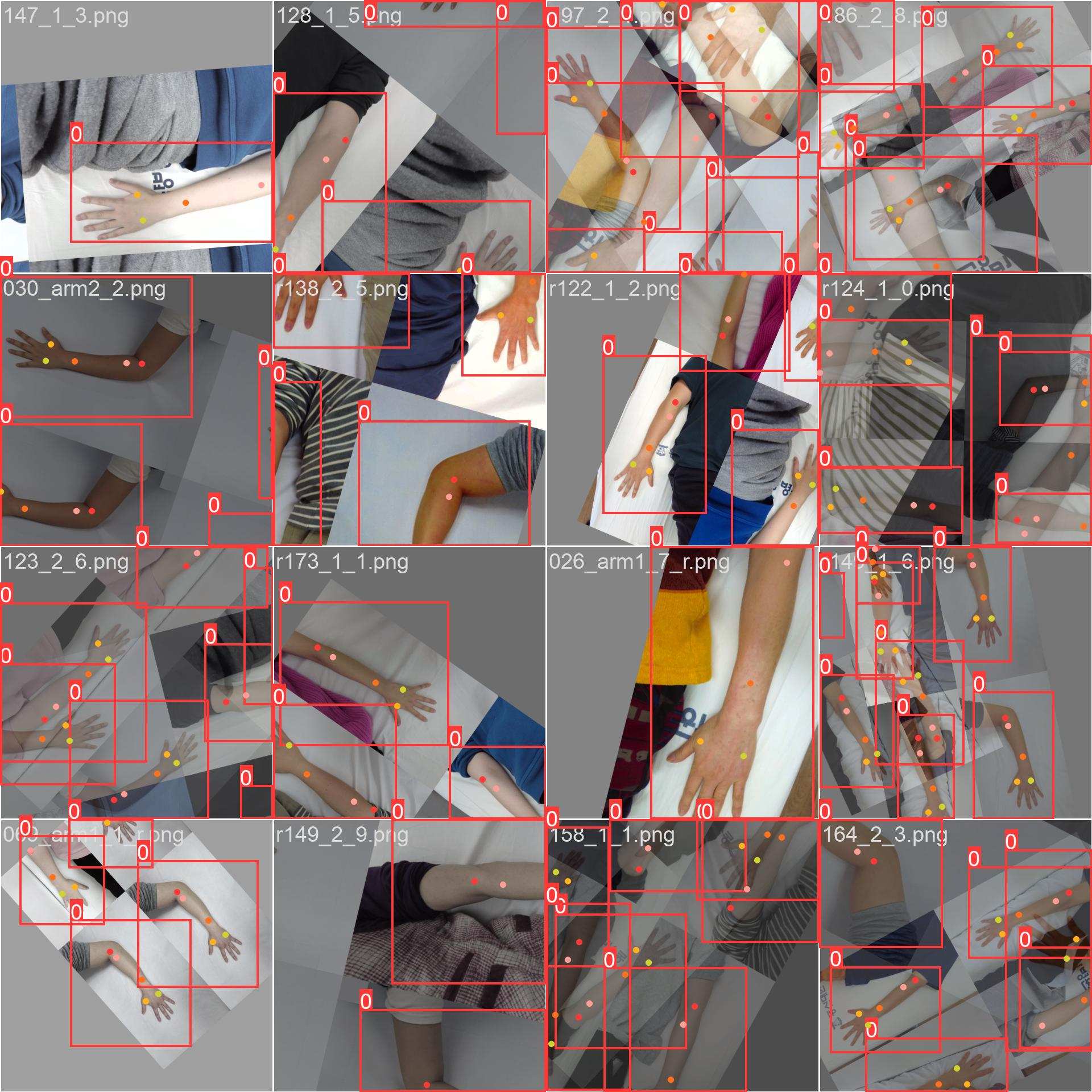


Figure S1. Example augmented input images used for training the model. Techniques such as rotation, scaling, mosaic, mix-up, and cropping were applied to the original training images to increase diversity.


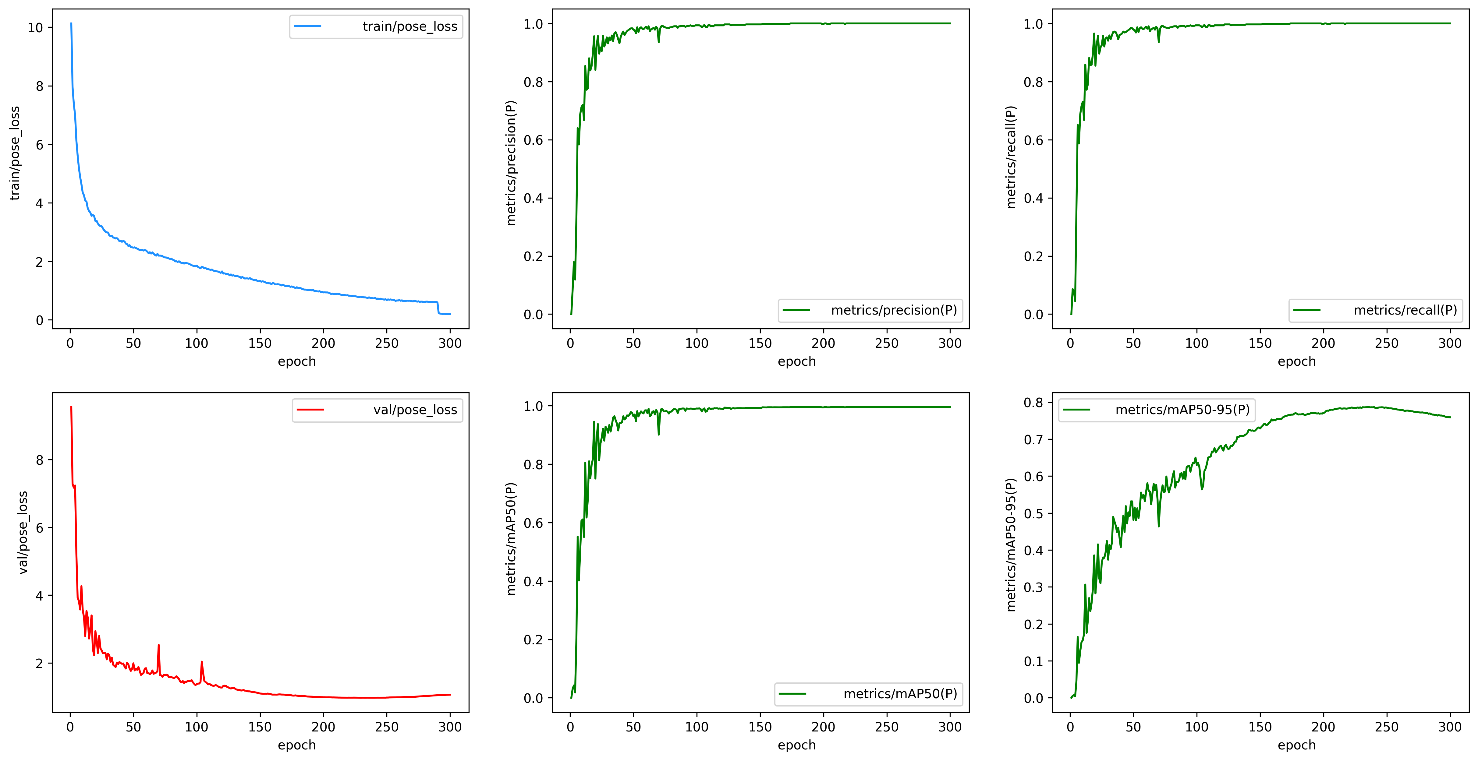


Figure S2. Training and validation metrics during model optimization. The similar trajectories between the training and validation results demonstrate good convergence of the YOLOv8l-pose model for acupoint detection.

(a)


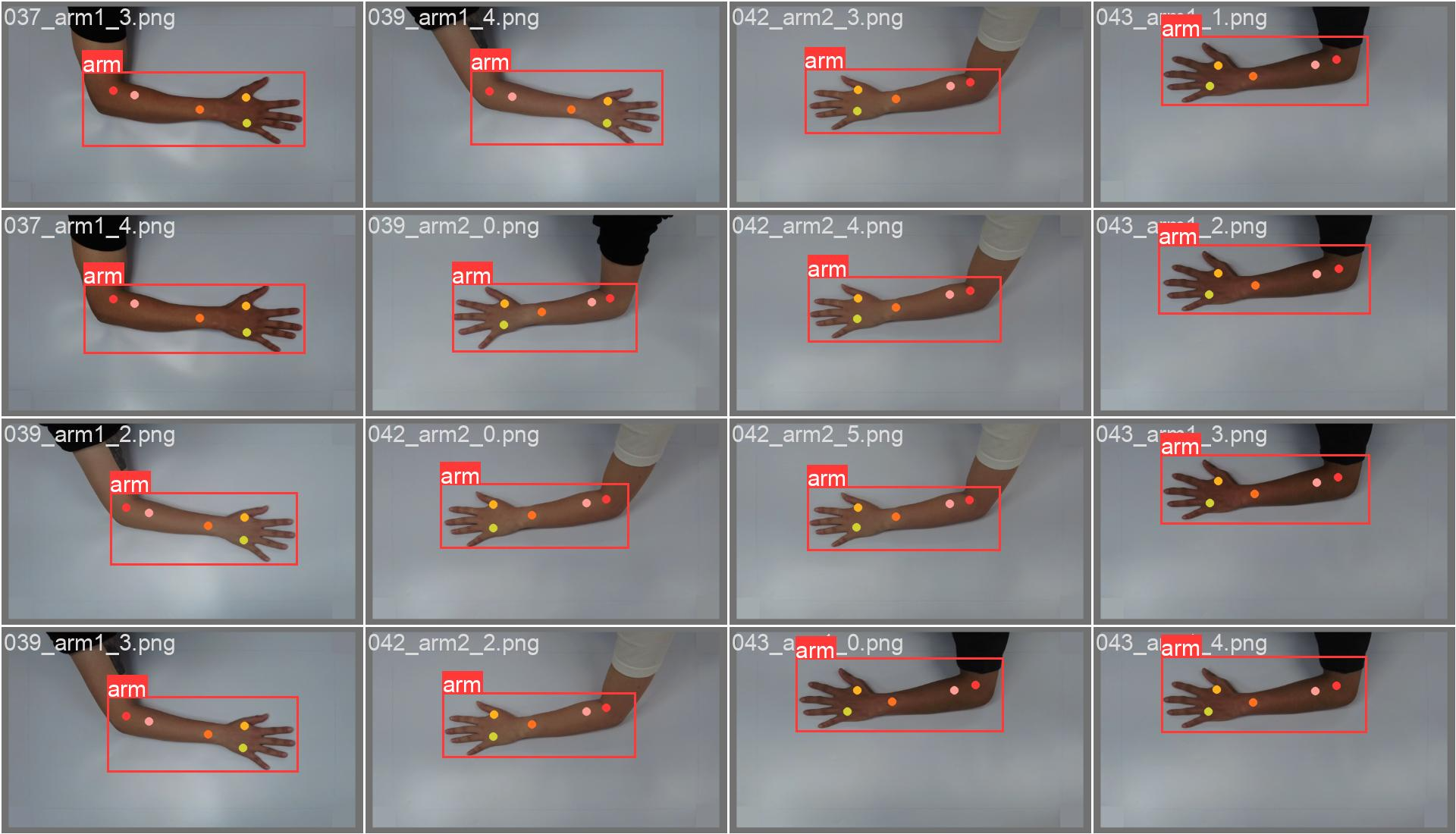

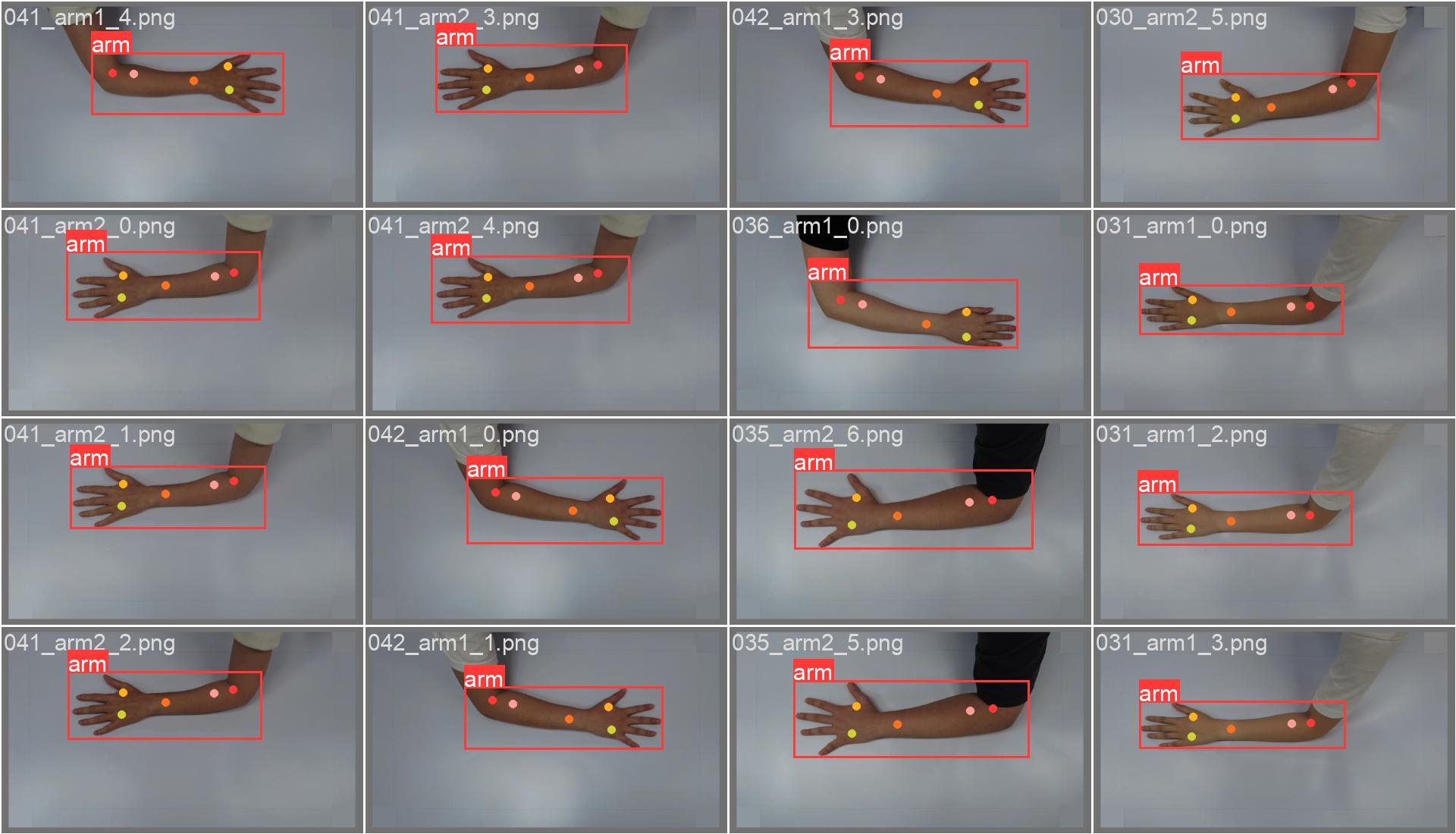


(b)


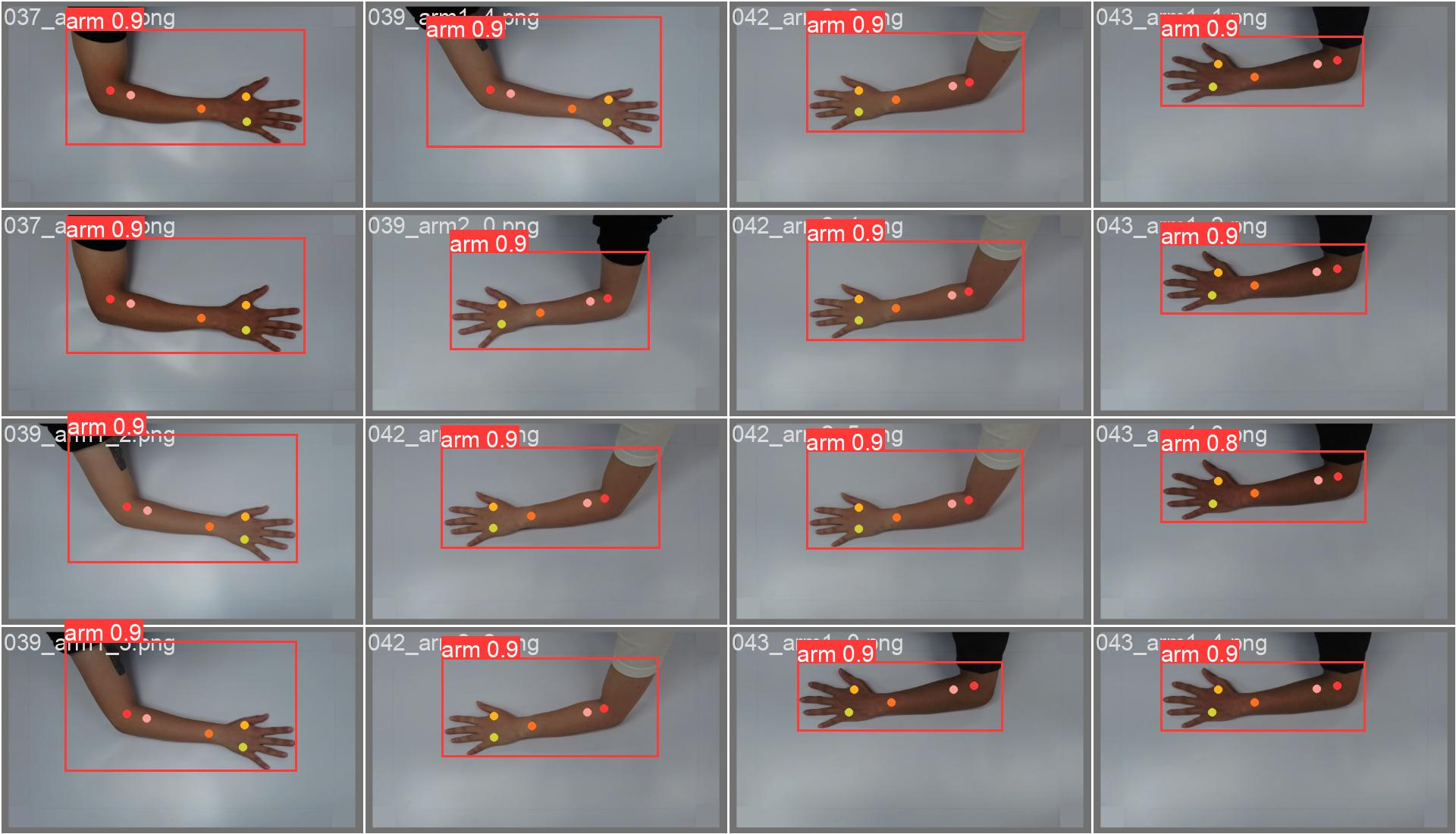

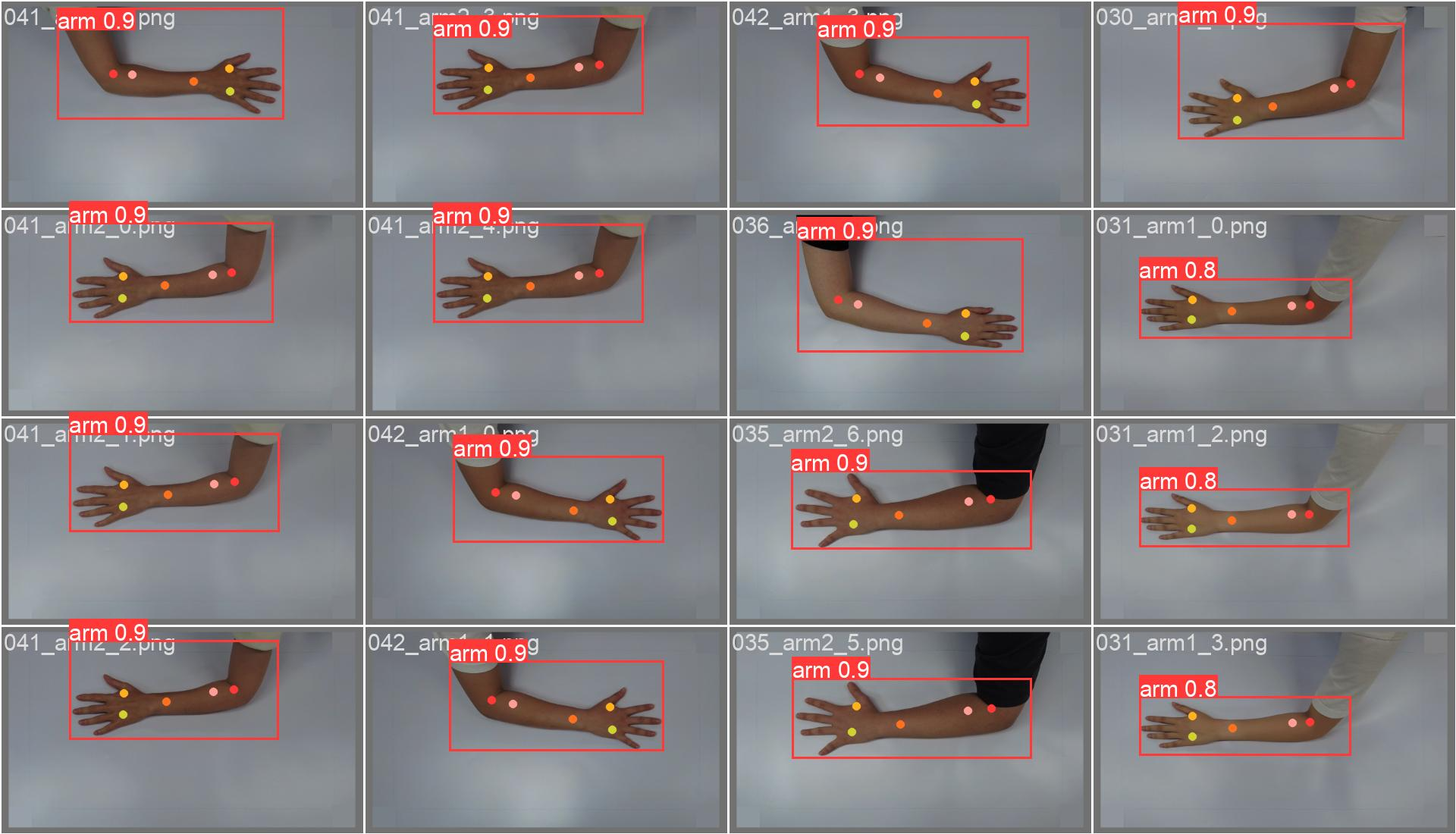


Figure S3. Example outputs of YOLOv8-pose model on the validation set for acupoint localization. The images show the model's detected acupoint locations (marked by dots) on two different validation batch samples. (a) Ground truth, (b) Prediction.


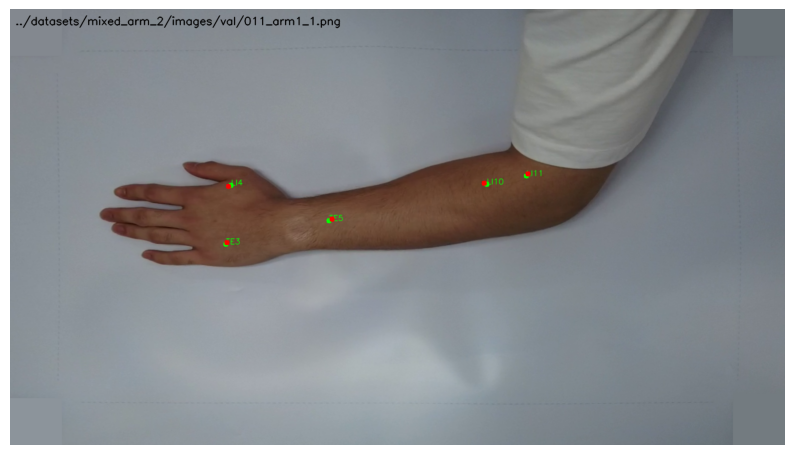

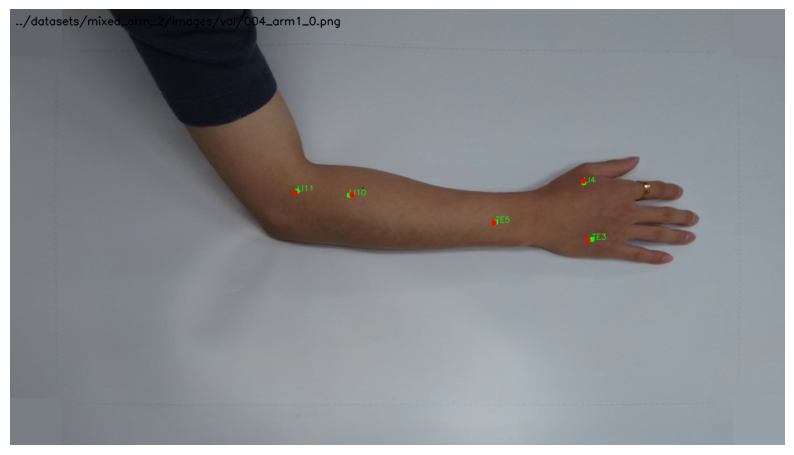


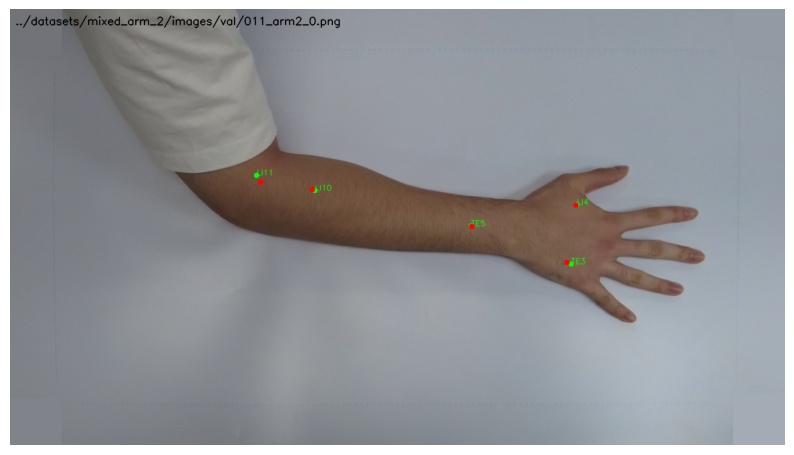

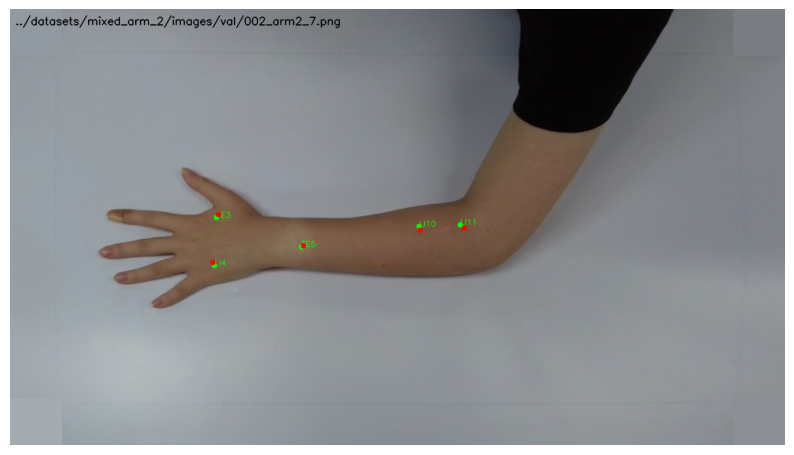


Figure S4. Visual comparison of detected and ground truth acupoint locations. The images show the acupoint locations predicted by the model (red dots) overlaid on the input images. The ground truth acupoint locations are indicated by green dots. The close alignment between the predicted and true acupoint positions demonstrates the accuracy of the model's localization capabilities. However, minor differences between some predicted and ground truth pairs can be detected. Overall, the results validate the potential of the model for precise acupoint localization in medical setting images.


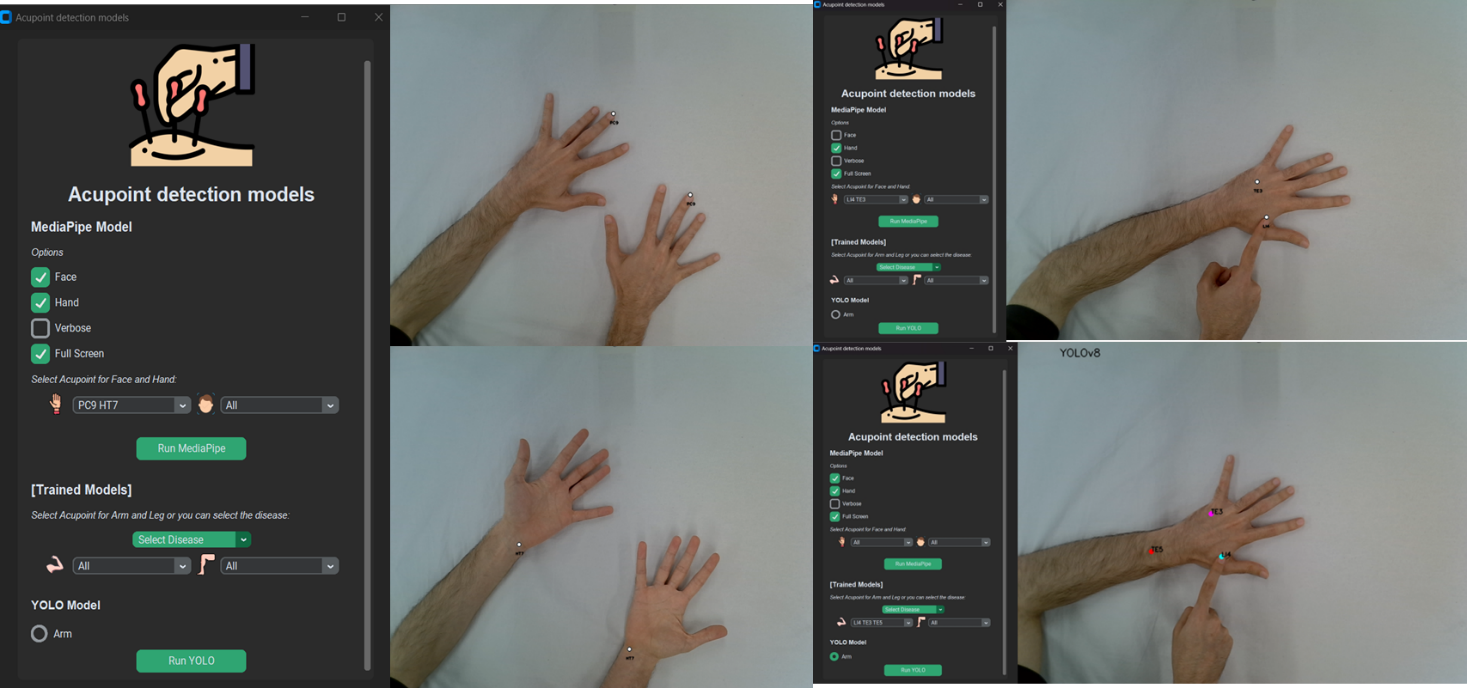


Figure S5. Acupoint Detection Software Interface and Results. The left panels showcase the user interface, featuring options for MediaPipe detection models and selectable body parts (face, hand). The right panels present two sample images of hands and forearms, each with small markers indicating detected acupoints. These sample images demonstrate the software's capability to identify and highlight specific points on the hand and arm. The interface and results together exemplify the integration of modern computer vision technology with traditional medical practices.
