# Supplementary figures and images for: Real-time location of acupuncture points based on anatomical landmarks and pose estimation models
Source: Front Neurorobot. 2024 Nov 8;18:1484038. doi: 10.3389/fnbot.2024.1484038 (PMC11609928; doi:10.3389/fnbot.2024.1484038)

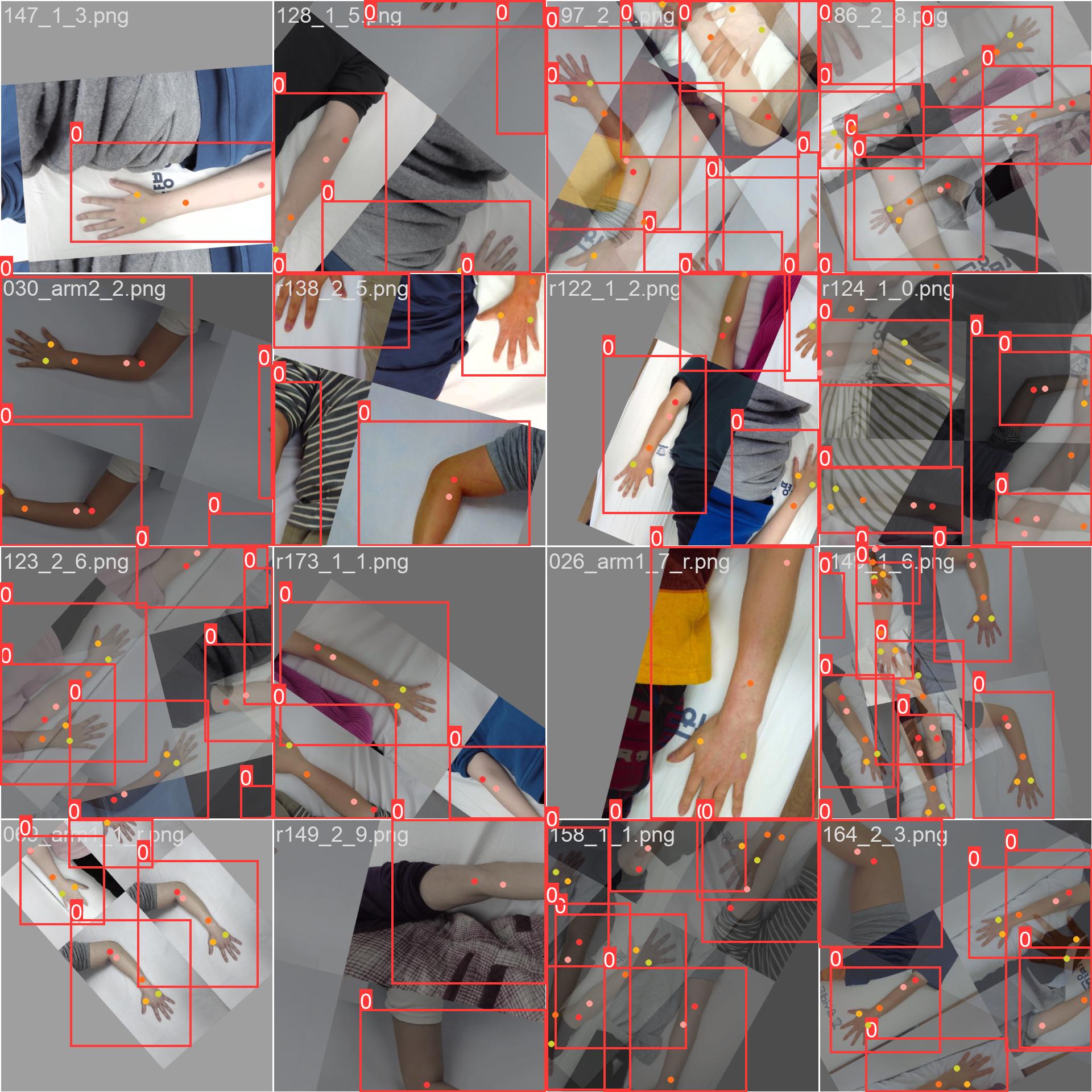

Supplement: SUPPLEMENTARY VIDEO S1 — Real-time facial acupoint detection using MediaPipe. [file Data_Sheet_1.ZIP › Supplementary/figS1_input.jpg]

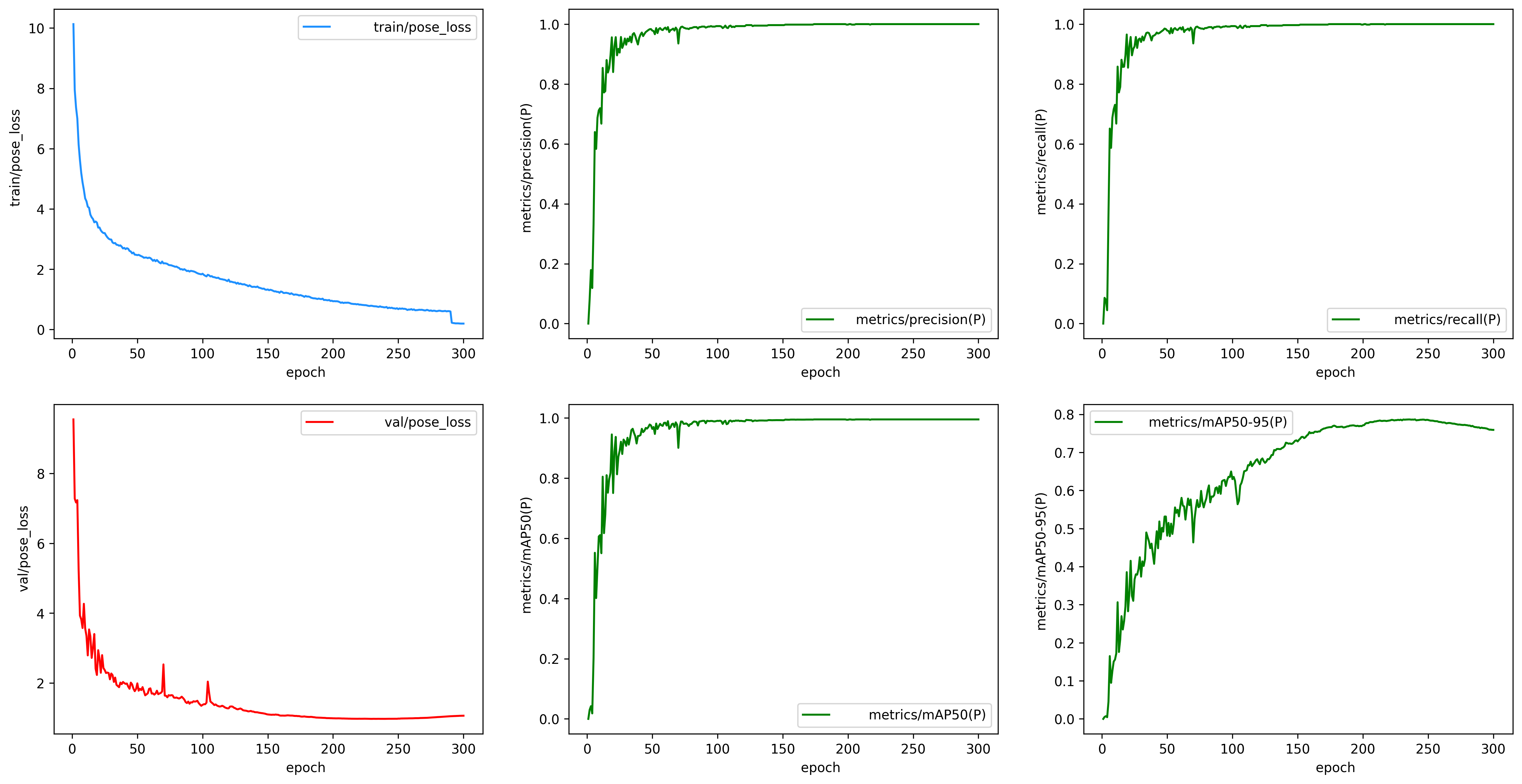

Supplement: SUPPLEMENTARY VIDEO S1 — Real-time facial acupoint detection using MediaPipe. [file Data_Sheet_1.ZIP › Supplementary/figS2_result.png]

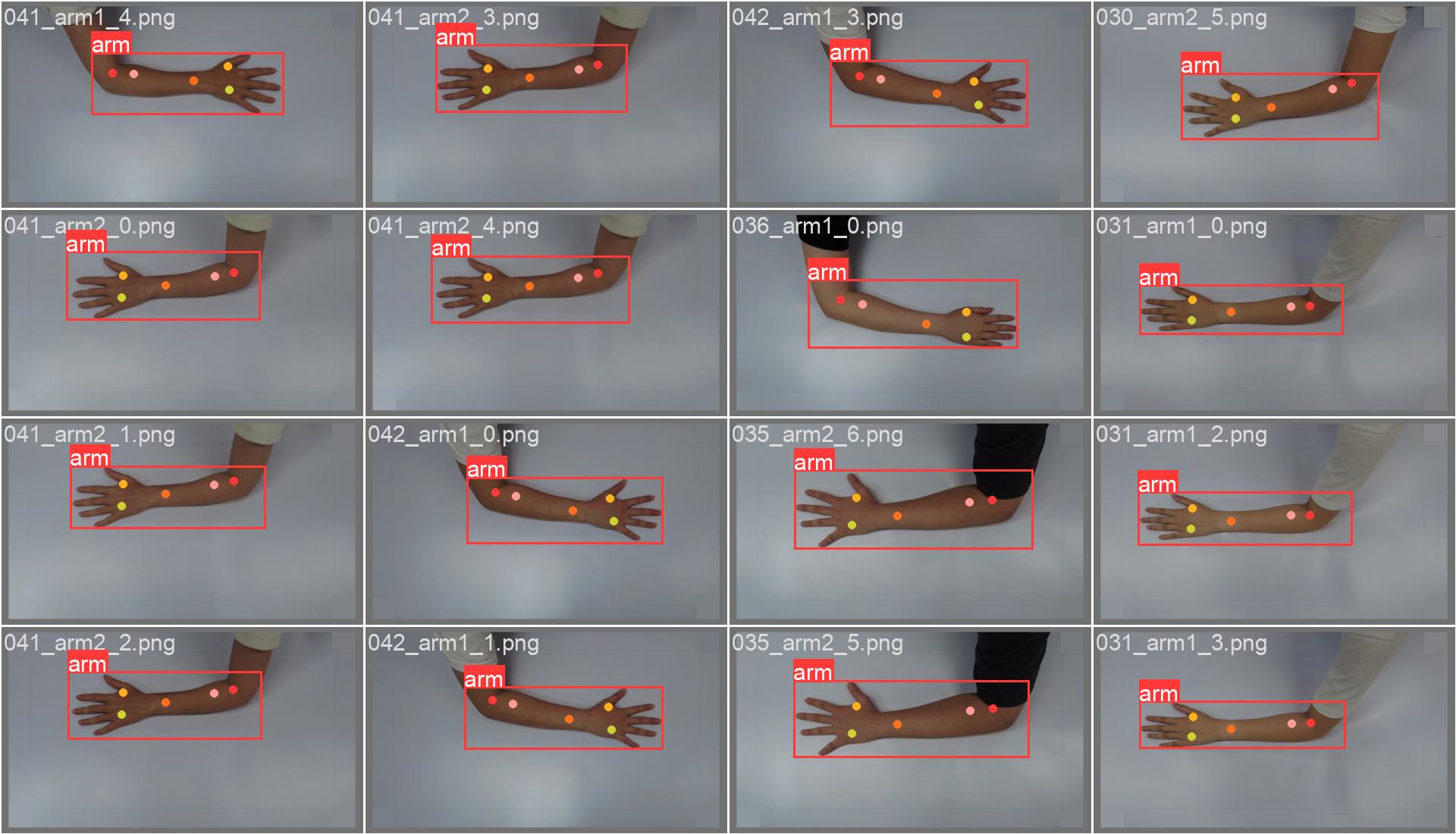

Supplement: SUPPLEMENTARY VIDEO S1 — Real-time facial acupoint detection using MediaPipe. [file Data_Sheet_1.ZIP › Supplementary/figS3 (1).jpg]

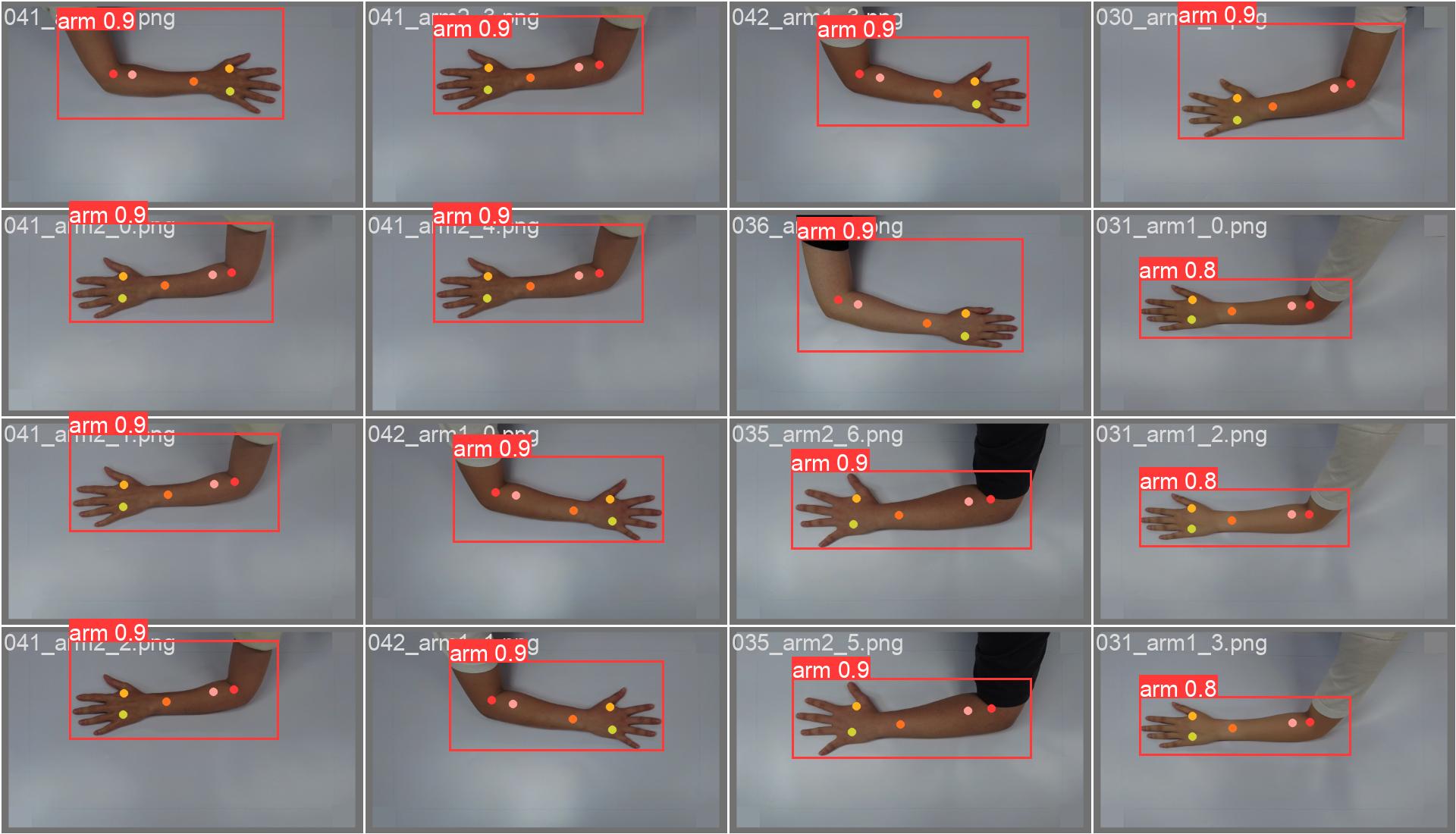

Supplement: SUPPLEMENTARY VIDEO S1 — Real-time facial acupoint detection using MediaPipe. [file Data_Sheet_1.ZIP › Supplementary/figS3 (2).jpg]

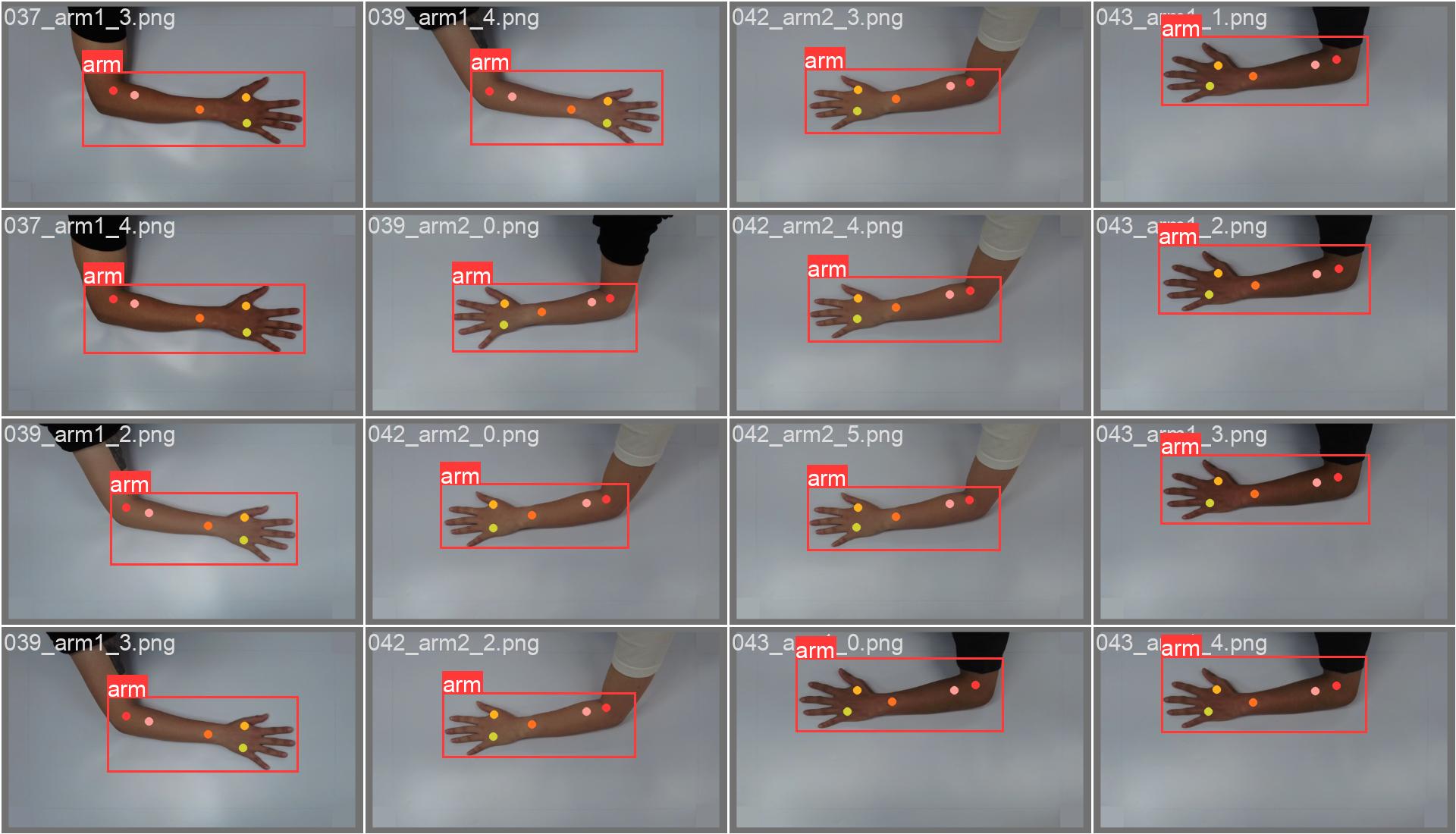

Supplement: SUPPLEMENTARY VIDEO S1 — Real-time facial acupoint detection using MediaPipe. [file Data_Sheet_1.ZIP › Supplementary/figS3 (3).jpg]

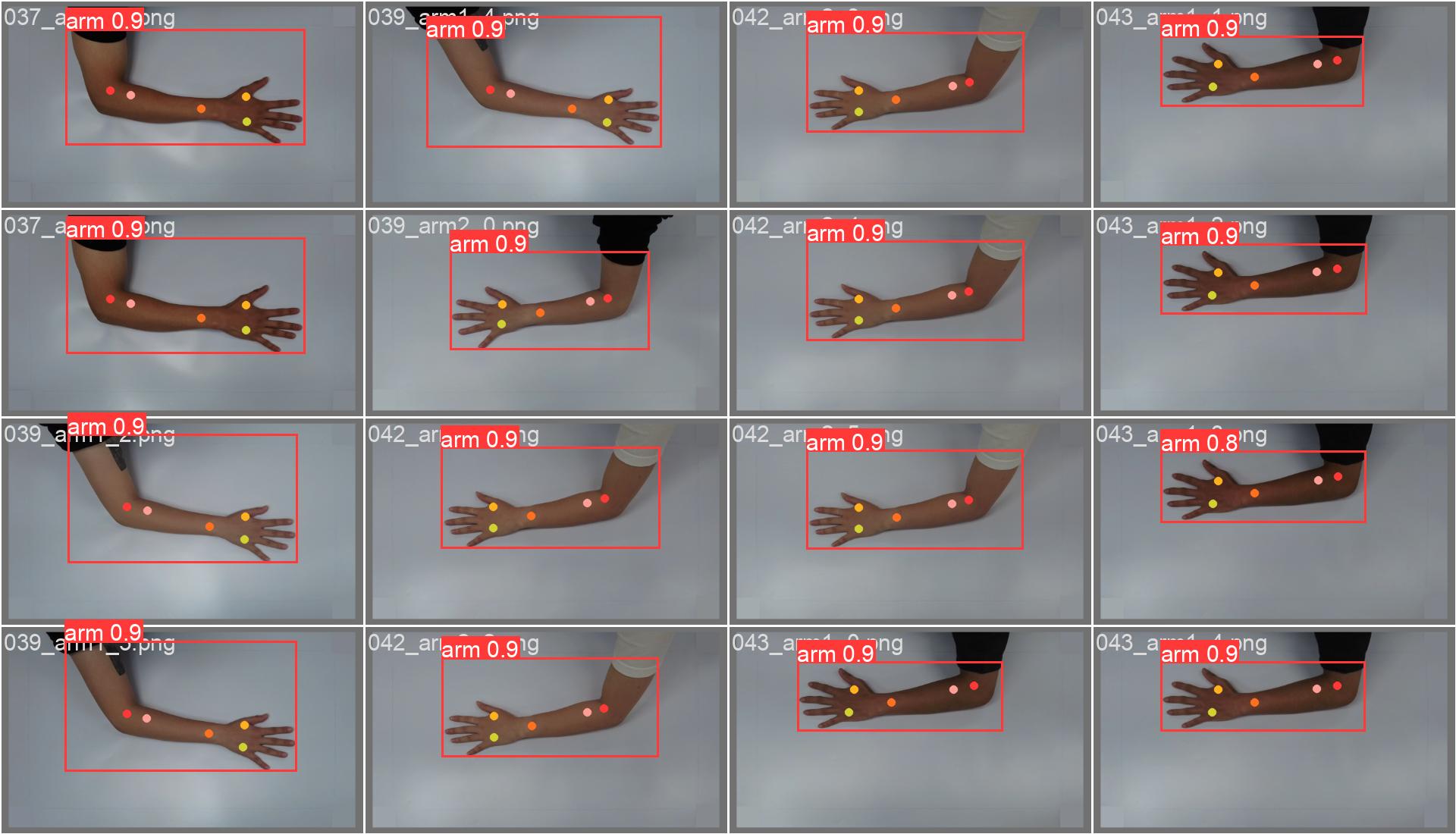

Supplement: SUPPLEMENTARY VIDEO S1 — Real-time facial acupoint detection using MediaPipe. [file Data_Sheet_1.ZIP › Supplementary/figS3 (4).jpg]
